# Free-Energy Landscape and Rate Estimation of the Aromatic Ring Flips in Basic Pancreatic Trypsin Inhibitors Using Metadynamics

**DOI:** 10.1021/acs.jctc.3c00460

**Published:** 2023-09-12

**Authors:** Mandar Kulkarni, Pär Söderhjelm

**Affiliations:** Division of Biophysical Chemistry, Lund University, Chemical Center, 22100 Lund, Sweden

## Abstract

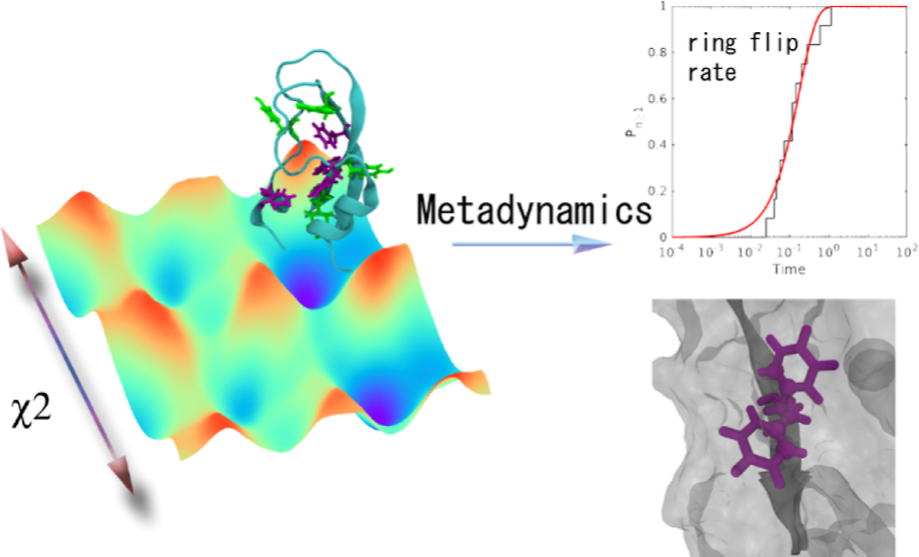

Aromatic side chains (phenylalanine and tyrosine) of a protein flip by 180° around the *C*_β_–*C*_γ_ axis (χ_2_ dihedral of the side chain), producing two symmetry-equivalent states. The study of ring flip dynamics with nuclear magnetic resonance (NMR) experiments helps to understand local conformational fluctuations. Ring flips are categorized as slow (milliseconds and onward) or fast (nanoseconds to near milliseconds) based on timescales accessible to NMR experiments. In this study, we investigated the ability of the infrequent metadynamics approach to estimate the flip rate and discriminate between slow and fast ring flips for eight individual aromatic side chains (F4, Y10, Y21, F22, Y23, F33, Y35, and F45) of the basic pancreatic trypsin inhibitor. Well-tempered metadynamics simulations were performed to estimate the ring-flipping free-energy surfaces for all eight aromatic residues. The results indicate that χ_2_ as a standalone collective variable (CV) is not sufficient to obtain computationally consistent results. Inclusion of a complementary CV, such as χ_1_(C_α_–C_β_), solved the problem for most residues and enabled us to classify fast and slow ring flips. This indicates the importance of librational motions in ring flips. Multiple pathways and mechanisms were observed for residues F4, Y10, and F22. Recrossing events were observed for residues F22 and F33, indicating a possible role of friction effects in ring flipping. The results demonstrate the successful application of infrequent metadynamics to estimate ring flip rates and identify certain limitations of the approach.

## Introduction

**Figure 1:**
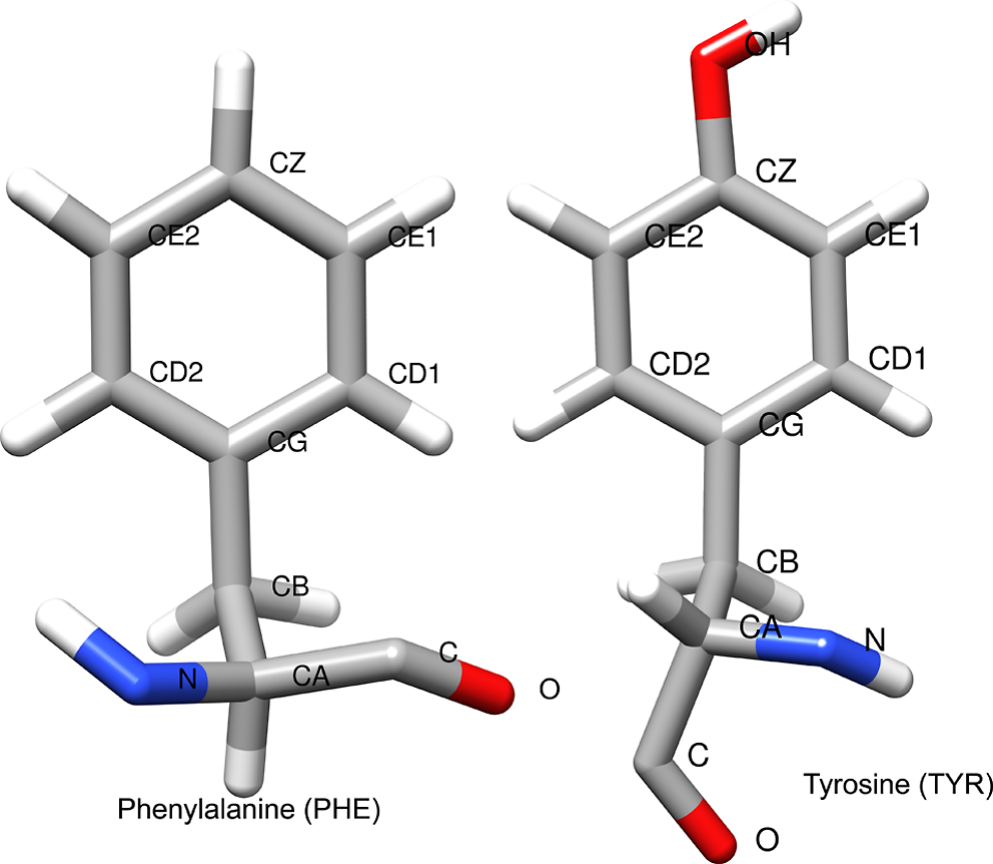
Atom names of tyrosine and phenylalanine indicate torsion χ_2_ (CA–CB–CG–CD1) and χ_1_ (N–CA–CB–CG).

The fluctuations of protein residues in their native state produce an ensemble of interconvertible substates.^1^ These dynamic protein fluctuations occur over a wide range of timescales. For example, the ring flip motions (180° rotation around *C*_β_–*C*_γ_ axis) of aromatic side chains in globular proteins occur from nanoseconds^2^ to seconds.^3^ The presence of different timescales for aromatic ring flipping in proteins was initially observed in seminal nuclear magnetic resonance (NMR) studies by Wüthrich and co-workers^4,5^ while studying the internal dynamics of basic pancreatic trypsin inhibitor (BPTI). The value of the side chain torsion angle χ_2_ (C_α_–C_β_–C_γ_–C_δ1_/CA–CB–CG–CD1 in Figure 1) acts as an indicator of aromatic ring flipping. The ring flipping process occasionally involves librational motions relative to backbone atoms, identified using the χ_1_ (N–C_α_–C_β_–C_γ_/N–CA–*CB*–CG) torsion. The aromatic side chains are often utilized as internal NMR probes to understand the protein interior dynamics surrounding these side chains.^6−9^

Aromatic ring flips are not directly associated with biological functions^10^ but rely on transient packing defects around the aromatic ring, which might be relevant to small-molecule binding or internal protein hydration. Ring flipping events in the protein interior are associated with breathing motion, a transient cavity expansion exposing the protein interior. Such breathing motions provide a transient formation of the necessary volume (activation volume^9,11^) required for the aromatic ring flip. This is a well-established model often referred to as the “cavity model”.^12^ Earlier ring flipping studies proposed a “diffusion model”^13,14^ based on Kramer’s theory, assuming that ring flipping is a diffusion-limited process originating from the frictional effects exerted by surrounding atoms.

In this study, we investigated four tyrosine (Y10, Y21, Y23, and Y35) and four phenylalanine (F4, F22, F33, and F45) residues of BPTI. These aromatic side chains contribute significantly to the stability of BPTI, and mutations^15−17^ at these sites with alanine or leucine destabilize the protein at room temperature. The NMR investigation of the globular protein BPTI by Wagner et al.^5^ was the first study to distinguish slow- and fast-flipping rings. In general, the aromatic ring flips are classified^5,18^ as slow flips (*k*_flip_ ≤ 10^3^ s^–1^, i.e., *t*_flip_ ≥ 10^–3^ s) and fast flips (*k*_flip_ > 10^3^ s^–1^, i.e., *t*_flip_ < 10^–3^ s) based on the flipping time (*t*_flip_) accessible to NMR methods. Experimentally, only slow ring flips can be studied using ^13^C CPMG relaxation experiments^3,11^ by observing signal splitting of symmetrically located δ or ε nuclei on the ring^4^ (CD1, CD2, CE1, CE2 positions in Figure 1). Experimental NMR studies of BPTI using ^13^C relaxation dispersion^3^ observed that the activation enthalpy of slow-flipping rings Y23, Y35, and F45 in BPTI is around 20 kcal mol^–1^. The abundance of experimental observations related to all eight aromatic residues in BPTI makes it a suitable model system for understanding the thermodynamics and kinetics of the ring flipping mechanism via molecular dynamics (MD) simulations.

It is possible to perform MD simulations up to a few microseconds within a reasonable time due to advances in computing power and GPU-based MD codes. In a 1 ms-long BPTI simulation (employing the Amber99SB-I protein force field and the TIP4P-Ew water model) by D.E. Shaw Research (DESRES), ring flipping was observed for seven of the eight aromatic side chains.^19^ The sparsity of ring flip events in the DESRES simulation suggests that the ring flipping of aromatic side chains in BPTI is a rare event. The sampling problem of rare events is generally resolved using enhanced sampling techniques such as metadynamics.^20,21^ In this study, well-tempered metadynamics^20^ (WTMetaD) simulations are performed to understand the free-energy landscape of ring flipping for all eight aromatic side chains of BPTI.

The successful application of metadynamics provides thermodynamic and structural details of the stable states. However, it is not trivial to evaluate the kinetics of the process under study using such a nonequilibrium approach. The acceleration of rare events by applying an external biasing potential corrupts the actual dynamics of the process. The infrequent metadynamics^22^ method (InMetaD) was recently proposed to recover accurate transition times from slowly (infrequently) biased metadynamics simulations. The InMetaD approach to obtaining kinetic information is still in its infancy. Previous studies^23−25^ have demonstrated the InMetaD method’s efficiency in studying millisecond timescale dynamics in protein–ligand interactions. We here perform InMetaD^22^ simulations to obtain the first passage times for transitions between symmetry-equivalent flipped configurations for all eight aromatic rings in BPTI. Analyses of the simulations allow us to propose a ring flipping mechanism for each ring, identify intermediates, and calculate ring flip rates.

## Methodology

### Simulation Setup

The initial coordinates for the BPTI were obtained from PDB ID 5PTI, and deuterium atoms and water molecules were removed. The ionizable residues were modeled based on their state at pH 7. The system was built using the tleap module of AmberTools18^26^ and then converted to GROMACS format using the ACPYPE Python script.^27^ The protein molecule was modeled using the AMBER ff14SB^28^ force field. The protein was first placed in a truncated octahedral box and was solvated by 4302 four-point TIP4P-Ew^29^ water molecules. Then, six Cl^–^ ions were added to neutralize the system. The Joung–Cheatham parameters^30^ for chloride ions were used. The distance between walls and protein was 10 Å. All simulations were performed with a time step of 2 fs, and all covalent bonds were constrained using the LINCS algorithm. A cut-off distance of 10 Å was used for short-range Coulomb and Lennard–Jones interactions. A long-range dispersion correction to the energy and pressure was applied. The long-range electrostatic interactions were treated with PME and a Fourier grid spacing of 1.2 Å with fourth-order spline interpolation. Periodic boundary conditions were applied, and the system was equilibrated first using the isothermal-isobaric (*NPT*) ensemble for 1.2 ns. During equilibration, the Berendsen^31^ thermostat and barostat were used to maintain 300 K temperature and 1 bar pressure. A restraining force of 25 kcal mol^–1^ Å^–2^ was used on heavy protein atoms during this stage. Then the structure was minimized using the steepest-descent method. The restraining force was reduced to 5 kcal mol^–1^ Å^–2^, and *NPT* equilibration was performed for 1 ns, followed by system minimization. After this stage, a 10 ns *NPT* equilibration run was performed using the Parrinello-Rahman barostat^32^ (coupling constant 1.0 ps) and Bussi-Donadio-Parrinello’s V-rescale^33^ thermostat (coupling constant 0.5 ps). The compressibility was set to 4.5 × 10^–5^ bar^–1^. The structure was minimized, and 100 ns of unbiased simulation was performed (*NVT* ensemble). A preliminary analysis was performed on this simulation to understand local fluctuations in BPTI. No aromatic ring flipping was observed during this simulation. The last structure of this simulation was utilized further for metadynamics simulations as well as for all InMetaD simulations. All metadynamics simulations were performed in the *NVT* ensemble (*T* = 300 K) using GROMACS 2018.3^34,35^ and PLUMED 2.5.^36,37^

We have also tested the effect of the force field on ring flipping for residue F22 using (i) CHARMM36m^38^ and charmm TIP3P water model (referred to as c36m) and (ii) CHARMM36^39^ and standard TIP3P water model (referred to as c36). In the c36m and c36 simulations, all covalent bonds involving hydrogen were constrained using LINCS. The long-range dispersion correction to energy and pressure was absent for CHARMM c36 and c36m simulations. For CHARMM simulations, the PME method was used to calculate long-range electrostatic interactions with a 12 Å cut-off. The Lennard–Jones interactions were smoothly switched to zero between 10 and 12 Å.

### Metadynamics Simulations

Even the “fast” flipping residues exhibit microsecond to near millisecond timescales,^3^ which are still difficult to access by standard MD simulations. We performed WTMetaD simulations to understand the aromatic ring flipping mechanism and to observe possible intermediate states along the flipping pathway. The details of the metadynamics simulations are reported in Table 1. The selection of collective variables (CVs) is critical to correctly capturing the underlying free-energy landscape and all possible metastable states.

**Table 1:** List of CVs and Details of Metadynamics Parameters; Hill Width (σ; Radians, Except for CMAP Which Has Arbitrary Units) Hill Height (*w*; kJ mol^–1^), Bias Factor (γ), Frequency of Hill Addition (ν, ps). For WTMetaD, ν Is Always 1 ps

			WTMetaD		InMetaD		
residue	CV	σ	γ	*w*	γ	*w*	ν^a^
F4	χ_2_, χ_1_	0.07, 0.07	12	1.2	12	0.6	12
Y10	χ_2_, χ_1_	0.07,0.07	12	1.2	12	0.3	10
Y21	χ_2_	0.07	10	0.6	10	0.6	18
F22	χ_2_, χ_1_	0.07,0.07	12	1.2	8	1.2	12
F22 (c36)^a^	χ_2_, χ_1_	0.07,0.07	12	1.2	8	0.3	12
F22 (c36m)^b^	χ_2_, χ_1_	0.07,0.055	12	1.2	n.a^d^	n.a.	n.a.
Y23	χ_2_, CMAP	0.07,0.30	12	1.2	10	0.6	8
F33	χ_2_, χ_1_	0.07,0.07	8	1.2	10	0.6	12
Y35^c^	χ_2_, χ_1_	0.07,0.07	12	1.2	12	0.6	12
F45	χ_2_, χ_1_	0.07,0.07	12	1.2	10	0.6	8
F45 (c36)	χ_2_, χ_1_	0.07,0.07	n.a.	n.a.	12	0.6	8

a Charmm36/standard TIP3P simulations.

b Charmm36m/charmm TIP3P.

c Non-converged free-energy surface.

d n.a.: simulations not performed in this case.

In the present study, the χ_2_ dihedral angle was the obvious choice to promote ring flipping. In addition, most WTMetaD simulations needed the χ_1_ torsion to observe convergence, as librational motions relative to the backbone are needed for ring flipping.

For residue Y23, a contact-map-based CV (CMAP) was used along with χ_2_. This variable is defined using the following function
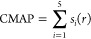
1where *s*_*i*_(*r*) is a switching function for each considered atom pair, given by
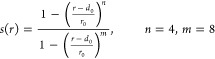
2

The values of *r*_0_ and *d*_0_ are mentioned in Table S1 in the Supporting Information and are based on equilibrium distances (obtained from unbiased simulation) between *C*_α_ atoms of five residue pairs, viz. A25-G28, G28-G56, C55-A25, C55-C5, and A25-C5. We note that CMAP is heuristic, and it is probably possible to define a better CV. In all metadynamics simulations, the hill width was around one-half of the standard deviation of the CV in a free simulation run.

In some additional InMetaD simulations, a repulsive harmonic wall potential (*V*_b_) was imposed to restrict sampling to specific ranges of either χ_2_ or χ_1_ values
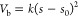
3where *k* is the force constant in kJ mol^–1^ rad^–2^, *s* is either χ_2_ or χ_1_, and *s*_0_ is the limiting value of *s*. The UPPER_WALLS and LOWER_WALLS keywords in PLUMED were used to apply the wall potential above and below *s*_0_, respectively. The details of the parameters *k* and *s*_0_ are provided in Table S5 in the Supporting Information.

A time-dependent metadynamics bias *V*(*s*,*t*) corrected with time-dependent bias offset^40^*c*(*t*) was used for reweighting.^36^ The expression of *c*(*t*) is shown below
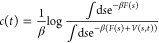
4where β is the thermodynamic beta and F(s) is the true free-energy surface. The evolution of *c*(*t*) with simulation time is shown in Figure S1 of the Supporting Information to understand the quasi-equilibrated region in WTMetaD simulations. We also tested the variational approach to conformational dynamics^41^ (VAC-MetaD) approach to avoid misleading results due to suboptimal CVs (see Appendix I in the Supporting Information for further details). The quasi-equilibrated part was utilized in the case of residue F22 and F33 to build CVs using VAC-MetaD.^41^

### Estimation of the Flipping Rate from Metadynamics Simulation

The reliability of InMetaD depends on two major factors, viz. (i) that chosen CVs should discriminate between metastable states and (ii) that no bias should be deposited in the transition state (TS) region. A converged free-energy surface in WTMetaD indicates that the same set of CVs can be utilized for the InMetaD simulations. In addition, we performed an additional set of InMetaD simulations only biasing the χ_2_ torsion, allowing us to understand the efficiency of χ_2_ alone to capture the ring flipping dynamics. The criterion to avoid bias addition at the TS region can be achieved by a slow (infrequent) addition of Gaussian bias during metadynamics. For each residue, *N* independent simulations were performed to obtain transition times, where *N* = 40 if not otherwise stated. For each *i*th individual run, the acceleration factor^22^ (α_*i*_) arising due to the slow addition of time-dependent bias along CV *s* is given as
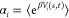
5

The exponentiated bias is averaged until the time of transition to another basin, i.e., the actual simulation time (*t*_*i*_^MD^). Then, the transition time (τ_*i*_) of this run is obtained as
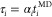
6

The average transition time (μ) is obtained as
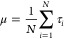
7

The transition times obtained from InMetaD should follow an exponential distribution according to the law of rare events. Salvalaglio et al.^42^ proposed a two-sample Kolmogorov–Smirnov (KS) test to test the Poisson characteristics of the InMetaD-derived transition times. In the KS test, the characteristic ring flip time (*t*_flip_) is obtained by fitting the empirical cumulative distribution function to the theoretical cumulative distribution function (TCDF)
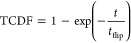
8This goodness of fit test helps to understand the deviation of simulated transition times from Poisson characteristics by observing the ratio of the average transition time (μ) to the median of transition times τ_*i*_ (*t*_m_) and the *p*-value (significance level 0.05). If the τ_*i*_ values follow an exponential distribution; the ratio μ ln 2/*t*_m_ should equal 1. This ratio is sensitive to the deviation from the exponential distribution and is reported in the present study along with *p*-values. We also report a 95% confidence interval for *t*_flip_ values (Table S2 in the Supporting Information) using a similar approach as proposed by Kříž et al.,^43^ i.e., assuming that the transition times are drawn from an exponential distribution so that their mean value follows a gamma distribution with a shape factor equal to the number of samples (although we used the fitted *t*_flip_ value as it has been observed to converge faster than the average (μ) in a previous study).^42^

The inverse of the fitted *t*_flip_ value is considered as the flipping rate (*k*_flip_)
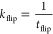
9

The InMetaD simulations were stopped when the trajectory crossed the barrier and transited to the stable state of interest (i.e., either the 180° flipped state or an intermediate state) by implementing boundary conditions based on biased CV values, monitored every 10 or 50 ps using the COMMITTOR keyword in PLUMED. The boundary conditions were defined closer to the product instead of precisely at the speculated TS CV values. This protocol assures a complete transition to a flipped state and avoids misinterpretation due to recrossing events. In the case of recrossing events, the trajectory does not continue to the flipped state after crossing a barrier but rather transits back to the starting state.

### Analysis of C14–C38 Disulfide Bridge Conformations

It has been observed in NMR experiments^44,45^ that residue C14 χ_1_ motions are 30-fold faster than residue C38 χ_1_, leading to a low-populated state *m* and a highly populated state *M*. The transition between disulfide bond orientations by rotation of χ_1_ torsion of C14 and C38 residues in BPTI is known as disulfide bond isomerization. The DESRES simulation observed five long-lived BPTI conformational states, each with different C14–C38 disulfide bond conformation. The ring flipping rates differed in each state, suggesting a relation between C14–C38 disulfide bond isomerization and ring flipping in BPTI. We reweighted the histogram of χ_1_(C14)−χ_1_(C38) and χ_2_(C14)−χ_2_(C38) to understand BPTI conformations sampled in converged WTMetaD simulations. WTMetaD simulation of χ_2_ and χ_1_ of Y35, and χ_3_ dihedral of the C14–C38 disulfide bond was also performed to understand its role in Y35 ring flip dynamics.

### Cluster Analysis

Cluster analysis was performed to identify intermediates and probable TS structures along the major ring flip pathway. The structures were clustered using the *gmx cluster* utility of the GROMACS package with the gromos^46^ method (cut-off of 0.1 nm). Please note that for reliable prediction of the TS ensemble, committor analysis^47,48^ and better clustering approaches^49^ are needed, as demonstrated by previous studies.

## Results and Discussion

We characterized aromatic ring flipping in BPTI in two complementary ways. First, we used WTMetaD simulations to calculate the free-energy surface and determine the most relevant intermediates. Second, we used the InMetaD method to calculate flipping rates and distinguish various mechanisms. An important part of the investigation consisted of choosing suitable CVs to be biased in both types of simulations.

The overall results obtained from the InMetaD investigation of each residue are shown in Table 2, together with the corresponding CVs used. The experimental ring flip rates^3^ (*k*_exp_) at 300 K and rates obtained from 1 ms-long DESRES simulation (*k*_des_) are also mentioned for comparison. We used the procedure in ref (3) to calculate *k*_exp_ values.

**Table 2:** InMetaD-Derived Ring Flip Time from Exponential Fit (*t*_flip_) at 300 K, Ring Flip Rate (*k*_flip_), KS-Test *p*-Value, and the Ratio of Average (μ) to a Median of Poisson Distribution from InMetaD Simulations, Experimental Ring Flip Rate (*k*_exp_) at 300 K, and from a 1 ms-Long DESRES Trajectory (*k*_des_)

residue	CVs	*t*_flip_ (s)	*k*_flip_ (s^–1^)	*p*-value	μ ln 2/*t*_m_	*k*_exp_ (s^–1^)	*k*_des_ (s^–1^)
F4	χ_2_, χ_1_	2.80 × 10^–5^	3.57 × 10^4^	0.32	1.79	>10^3^	7.0 × 10^6^
Y10	χ_2_, χ_1_	5.80 × 10^–5^	1.72 × 10^4^	0.80	1.54	>10^3^	1.0 × 10^6^
Y21	χ_2_	5.20 × 10^–3^	1.92 × 10^2^	0.68	1.32	<100^a^	1.0 × 10^3^
F22	χ_2_, χ_1_	2.41 × 10^–2^	4.15 × 10^1^	0.16	3.54	670^b^	7.0 × 10^4^
F22 (c36)^d^	χ_2_, χ_1_	5.45 × 10^–4^	1.83 × 10^3^	0.69	1.24	670^b^	7.0 × 10^4^
Y23	χ_2_, CMAP	9.81 × 10^–1^	1.02	0.09	1.80	140^c^	1.0 × 10^3^
F33	χ_2_, χ_1_	3.50 × 10^–5^	2.86 × 10^4^	0.03	2.47	>10^3^	6.0 × 10^6^
Y35	χ_2_, χ_1_	3.49	2.87 × 10^–1^	0.58	3.22	4^c^	1.0 × 10^4^
F45	χ_2_, χ_1_	3.22	3.11 × 10^–1^	0.95	1.44	220^c^	0
F45 (c36)^e^	χ_2_, χ_1_	1.78 × 10^–1^	5.61	0.97	1.40	220^c^	0

a 308 K.

b 278 K.

c Values estimated using the Arrhenius equation and with data from ref (3). Results from

d 16 simulations and

e 12 simulations.

### Free Energy and Flipping Mechanism

The detailed results for each residue are described in the following sections. For all free-energy profiles, the position of the starting configuration is denoted with state A and intermediates with *I*_*n*_(*n* = 1,2). The corresponding symmetry-equivalent states are indicated with prime notation (A^′^ and *I*_*n*_′, *n* = 1,2). Representative structures of A and *I_n_* states are shown for each residue. The convergence of metadynamics simulations and diffusion along CVs for each simulation are shown in the Supporting Information (Figures S2–S10). The distributions of InMetaD trajectory endpoints immediately after flipping (starting from state A) in each case are shown in Figures S11 and S12 in the Supporting Information.

Following the NMR-motivated “fast” and “slow” classification of aromatic ring flips, the fast-flipping residues (F4, F33, and Y10) are described first, and the remaining slow-flipping residues are described later. However, note that even the fast-flipping rings in BPTI exhibit *t*_flip_ values on the microsecond time scale, making it challenging to obtain statistically significant results. Residues F4 and Y10 (among other residues) could flip via multiple pathways; the *t*_flip_ values and KS-test-derived parameters for each mechanism are mentioned in Table 3. Plausible TS structures based on cluster analysis are presented in Figures S13 and S14 in the Supporting Information.

**Table 3:** Mechanism-Dependent Results of the InMetaD Simulations of Residues F4 and Y10

residue	mechanism	*N*_traj_^a^	*t*_flip_ (s)	*k*_flip_ (s^–1^)	*p*-value	μ ln 2/*t*_m_
F4	all	40	2.80 × 10^–5^	3.57 × 10^4^	0.32	1.79
	A ⇌ I1 ⇌ I1^′^ → A^′^	22	3.02 × 10^–5^	3.31 × 10^4^	0.82	1.48
	A ⇌ I1 → I2^′^	6	2.21 × 10^–5^	4.52 × 10^4^	0.71	0.80
	A → A^′^	12	2.81 × 10^–5^	3.56 × 10^4^	0.13	2.78
Y10	all	40	5.80 × 10^–5^	1.72 × 10^4^	0.80	1.54
	A ⇌ I1 → I1^′^	36	5.79 × 10^–5^	1.73 × 10^4^	0.89	1.54
	A → A^′^	4	3.42 × 10^–5^	2.92 × 10^4^	0.39	1.48

a *N*_traj =_ number of trajectories following a particular mechanism.

### Fast-Flipping Residues

#### Residue F4

**Figure 2:**
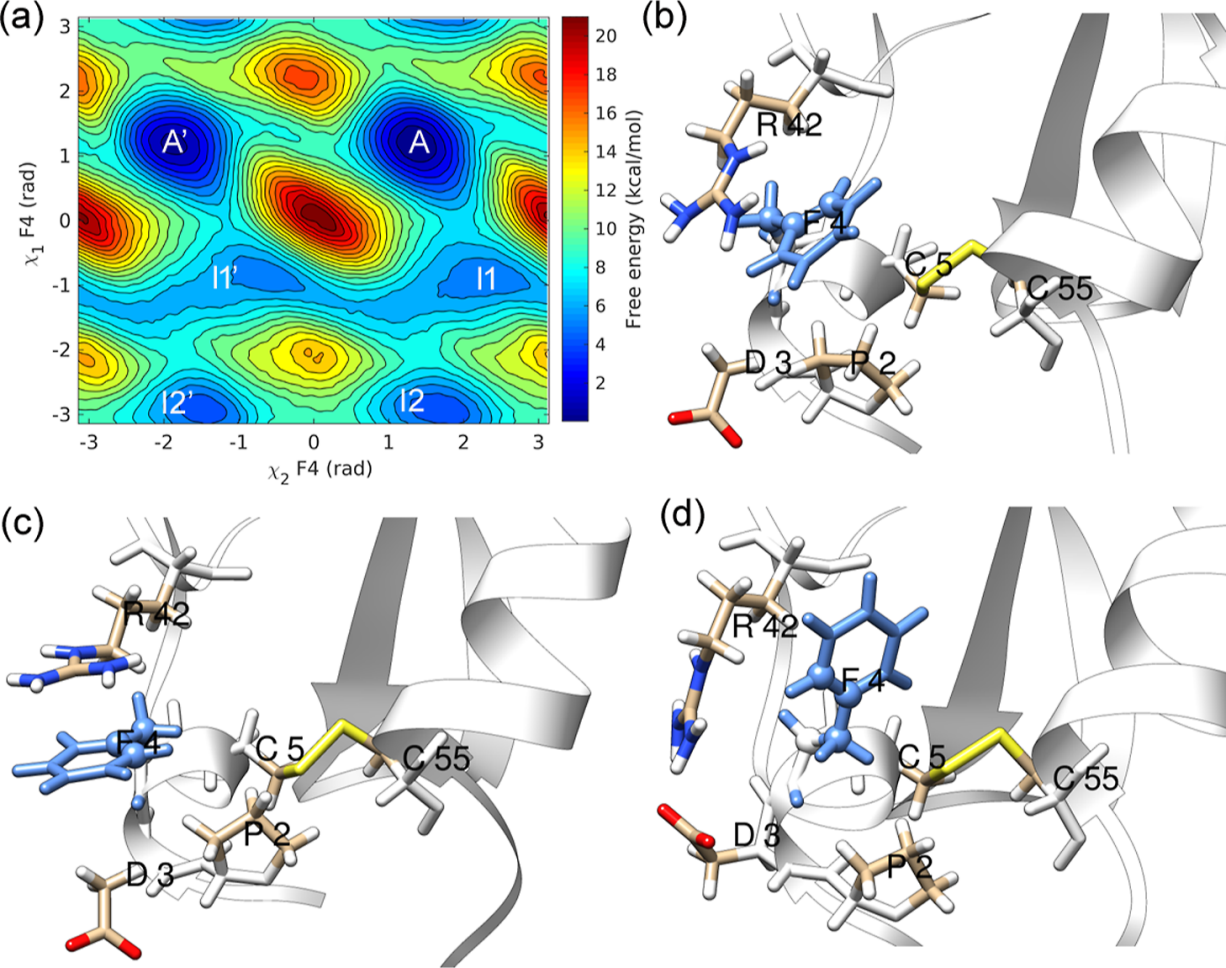
(a) Residue F4 (blue) ring flipping free-energy along χ_2_ and χ_1_ CVs and representative structures from (b) minimum A, (c) minimum I1, and (d) minimum I2.

Residue F4 is located on the solvent-exposed protein exterior and frequently interacts with residue R42. The free-energy surface is shown in Figure 2, together with representative structures. State A^′^ and state I2^′^ are considered “flipped” states, signaling that a transition has occurred, thus providing τ_*i*_(*i* = 1–40) values. The results are shown in Figure S15, and the bootstrap analysis of all 40 τ_*i*_ values and the *p*-value obtained from the KS test are shown in Figure S16 in the Supporting Information. The effective *t*_flip_ considering all 40 simulations is estimated to be 2.80 × 10^–5^ s.

State *I*1 is involved in the ring flipping of residue F4. The cation−π interactions with positively charged residue R42 stabilize the conformations in minimum *I*1. Residue F4 is close to the disulfide bond [5–55] formed by C5 and C55. The disulfide bond [5–55] torsion was not affected during states A to *I*1 transition because the aromatic side chain of F4 moved away from residue C5. State *I*2 has a trans orientation of the bonds N-CA (backbone) and CB–CG (side chain), thus exposing R42 to solvent. Salt bridge interactions were observed between R42 and D3 in state I2.

Three different mechanisms were observed during the InMetaD simulations, as described in Table 3. In the most prominent mechanism, denoted A ⇌ *I*1 ⇌ *I*1^′^ → A^′^, ring flips occur via coupled motions of the χ_2_ and χ_1_ torsions, starting from state *A*, and visiting state *I*1 multiple times before reaching state *I*1^’^ and eventually proceeding to A^′^. A direct ring flip from state A to A^′^ was also observed, but these trajectories exhibited a few *A* to *I*1 reversible transitions before the direct transition. Finally, six trajectories flipped to state *I*2^′^ while starting from state A. These trajectories represent an off-pathway mechanism where trajectories transition to state *I*2^′^ before proceeding to state *I*1^′^. In conclusion, multiple flipping pathways are possible with almost the same *t*_flip_ in the case of residue F4. In all of these mechanisms, reversible transitions between *A* and *I*1 occur due to motions along χ_1_.

#### Residue Y10

**Figure 3:**
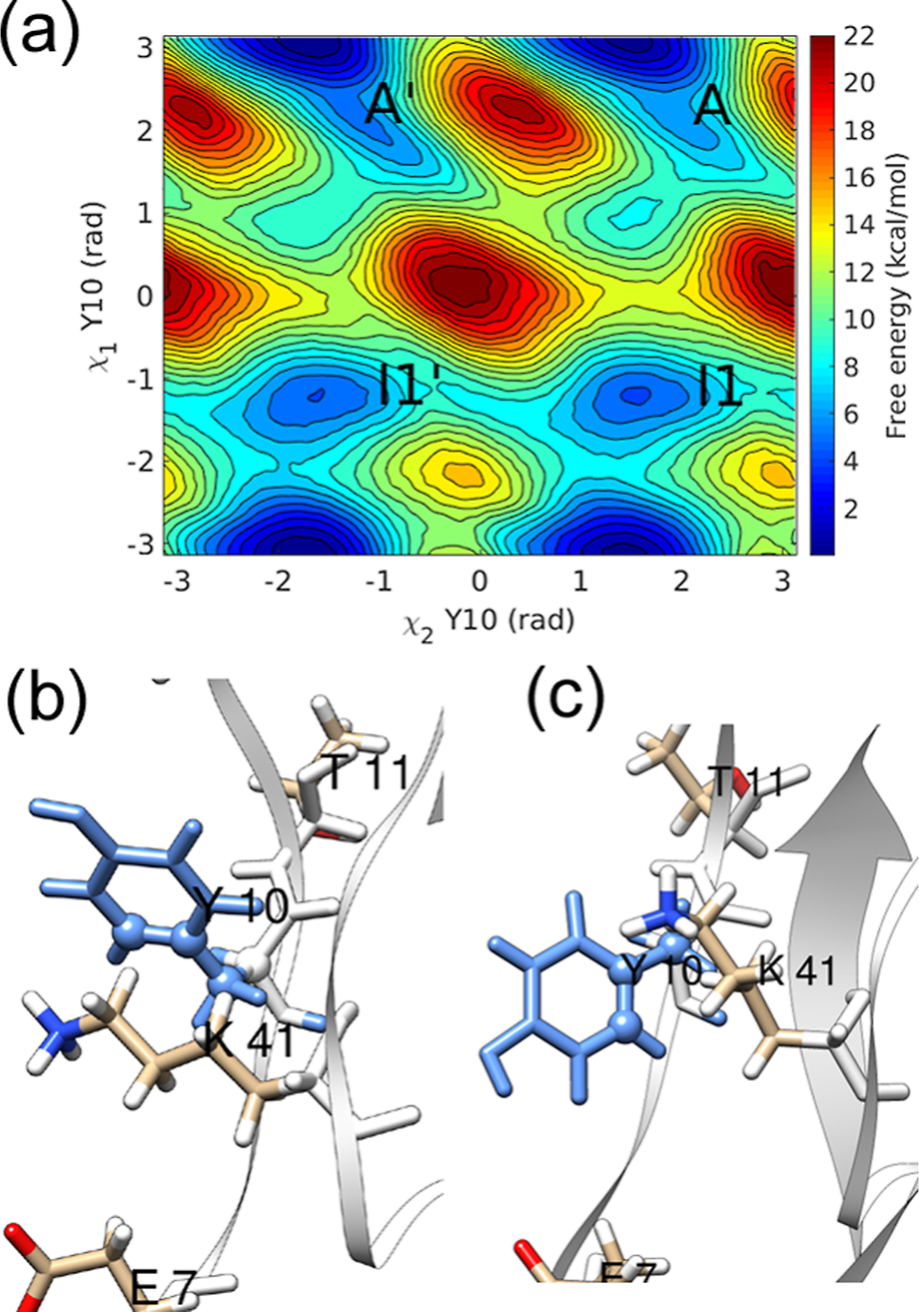
(a) χ_2_–χ_1_ free-energy surface and representative structures in (b) minimum A and (c) minimum I1 of the free-energy landscape of residue Y10 (blue).

We have observed two distinct mechanisms for flipping: a χ_2_–χ_1_ coupled mechanism and a direct ring flip. The prominent mechanism of residue Y10 involves motions along the χ_1_ torsion leading to the metastable state I1 (see Figure 3a).

In that state, residue Y10 is less restricted by interactions with surrounding atoms (see Figure 3c) as the motions along χ_1_ bring the Y10 aromatic side chain out of the core region surrounded by T11, K41, and P13. We have observed transient interactions with K41 but no hydrogen bonding interactions.

The aromatic ring flipping in Y10 illustrates an alternate mechanism to the “breathing cavity” mechanism proposed by earlier studies. This alternate mechanism involves moving the aromatic side chain to a less crowded region before undergoing the flipping process. This mechanism differs from the “cavity model,” where transient cavity formation allows ring flipping.

Most InMetaD trajectories (36 out of 40 simulations) followed the χ_2_–χ_1_ coupled mechanism involving state *I*1. A *t*_flip_ of 5.80 × 10^–5^ s was observed by considering all 40 InMetaD simulations. Figures S17 and S18 in the Supporting Information report the KS-test results and associated errors.

Four trajectories have transitioned to the flipped state *A*′. However, there were a few (one or two) *A*–*I*1 jumps that occurred before proceeding to state *A*′. Thus, state *I*1 is an important intermediate that could be an active or off-pathway state, depending on the transition path.

#### Residue F33

**Figure 4:**
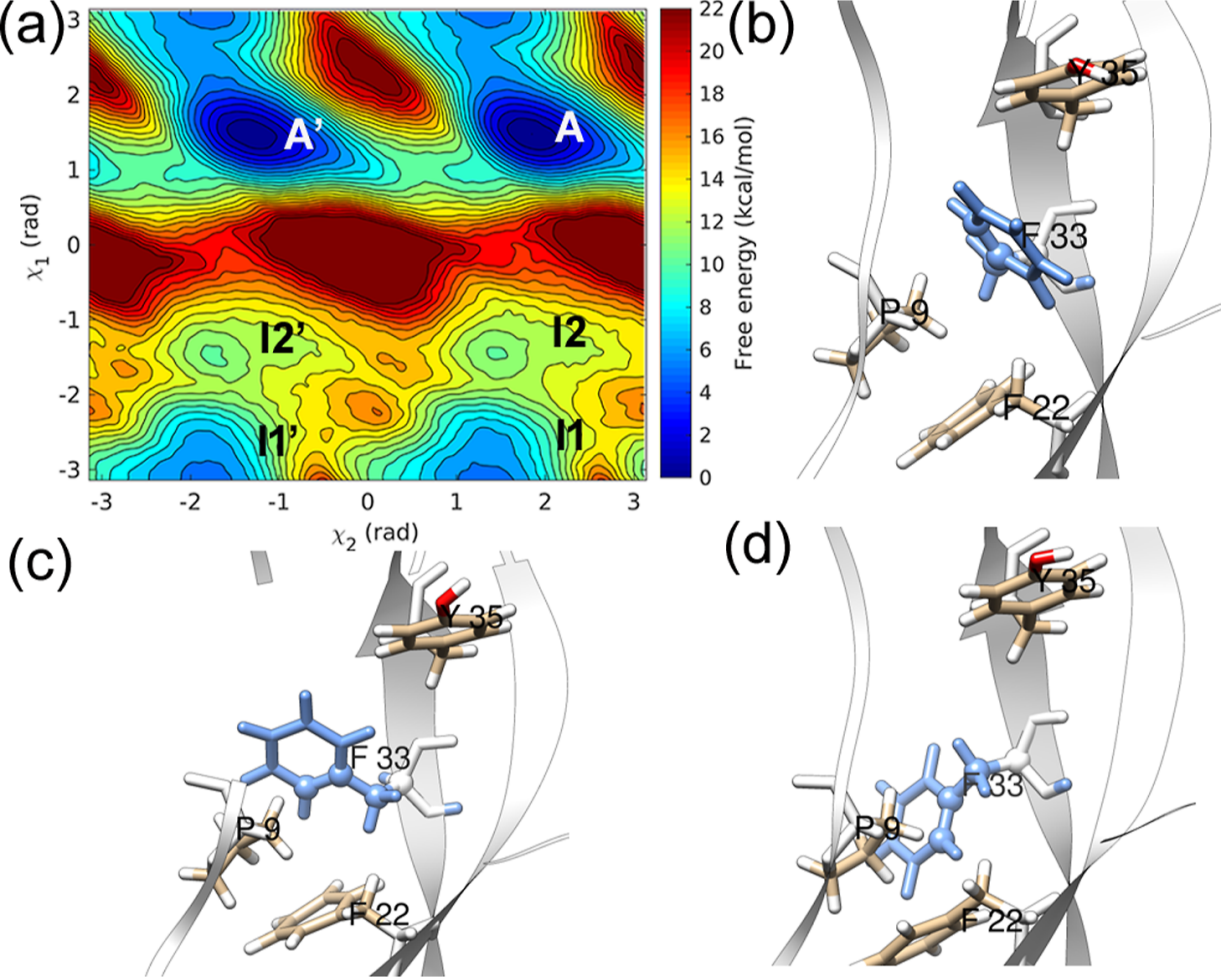
(a) χ_2_–χ_1_ free-energy surface and representative structures in (b) minimum *A*, (c) minimum *I*1, and (d) minimum *I*2 of the free-energy landscape of residue F33 (blue).

The residue F33 is surrounded by hydrophobic residues P9, F22, and Y35. The significant variation in values of χ_1_ torsion during WTMetaD simulations leads to states *I*1 and *I*2, where the aromatic side chain of F33 is out of the protein interior (see Figure 4). The flipping of F33 only required small fluctuations relative to the backbone. Thus, even though states *I*1 and *I*2 were observed in the free-energy landscape, the direct ring flipping pathway did not involve these states. All trajectories visited an off-pathway state *I*1 during the InMetaD simulations. The F33 flipping affects χ_1_ fluctuations of residue F22 (see Figure S19 in the Supporting Information). All InMetaD simulations showed flipping mainly by χ_2_ rotation with positive values of χ_1_ (Figure S11 in the Supporting Information).

A *t*_flip_ of 3.50 × 10^–5^ s was obtained, and the KS test gave a *p*-value of 0.03. To understand the small *p*-value, indicating significant deviation from the Poisson distribution, we analyzed the InMetaD trajectories and found deposition of bias in the barrier region along χ_1_ (χ_1_ ≈ 2.4 rad). The example of bias deposition in randomly chosen trajectories is shown in Figure S20 in the Supporting Information. Residue F33 demonstrated nonproductive off-pathway transitions between *A* and *I*1 states, whereas the main mechanism is direct flipping. To investigate whether these nonproductive pathways affected the results, we performed another set of 12 InMetaD simulations in which a repulsive wall bias restricted the sampling of χ_1_ values greater than 2.4 rad (see the Methodology section). The *t*_flip_ value from 12 simulations with wall bias is close to the previous χ_2_–χ_1_ InMetaD simulations without wall bias and shows a *p*-value of 0.52 (see Table S3 and Figures S21 and S22 in the Supporting Information).

### Slow-Flipping Residues

#### Residues Y21 and F45

**Figure 5:**
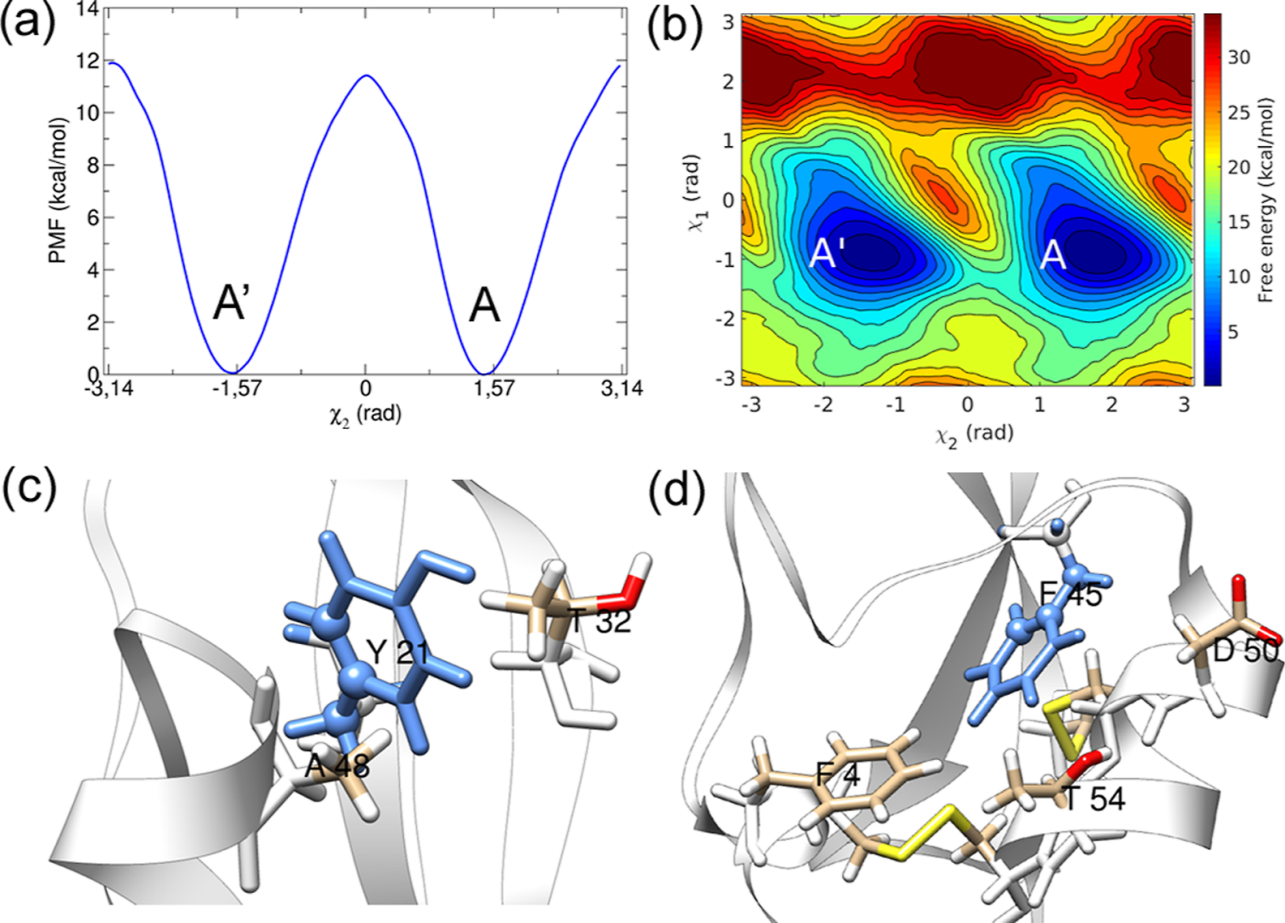
Free-energy of (a) residue Y21 (blue) along χ_2_, (b) residue F45 (blue) along χ_2_–χ_1_, (c) representative structure from minimum A of residue Y21, and (d) from minimum A of residue F45.

As shown in Figure 5, residues Y21 and F45 lack intermediates in the free-energy surface and flip via a simple direct ring flip mechanism. Residue Y21 displayed convergence with only the χ_2_ torsion as a CV, and the resulting *t*_flip_ = 5.20 × 10^–3^ s would classify it as a slow-flipping residue. Residue Y21 is solvent-exposed, and thus simple motion along the χ_2_ torsion seems to be sufficient as there is no steric hindrance with the surrounding protein atoms. Instead, most of the ring flips occur in a narrow range of χ_1_ values. The reweighted distribution of χ_2_–χ_1_ from the converged metadynamics simulation indicates that no significant motion along χ_1_ is needed, and Y21 can flip transiently. This residue lies on the rigid β-strand (I18-N24), which forms an anti-parallel β-sheet via strong interactions with another β-strand (L29-Y35). Interestingly, residue F33 also lies on a β-strand but is a fast-flipping residue. For residue F45, a converged free-energy surface was observed along the χ_2_–χ_1_ CVs, and thus, 40 InMetaD simulations were performed along the χ_2_–χ_1_ variables. The distribution of τ_*i*_ (*i* = 1 to 40) passed the KS test, and a fitted *t*_flip_ of 3.21 s was obtained, indicating F45 as a slow-flipping residue. There is direct flipping in F45; however, χ_1_ is needed for librational motions (see Figure 5d), which facilitate flipping along χ_2_ (InMetaD simulations based on only χ_2_ did not pass the KS test, as discussed below). The cumulative distribution function of τ_*i*_ and associated errors are shown in Figures S23 and S24 in the Supporting Information.

#### Residue F22

**Figure 6:**
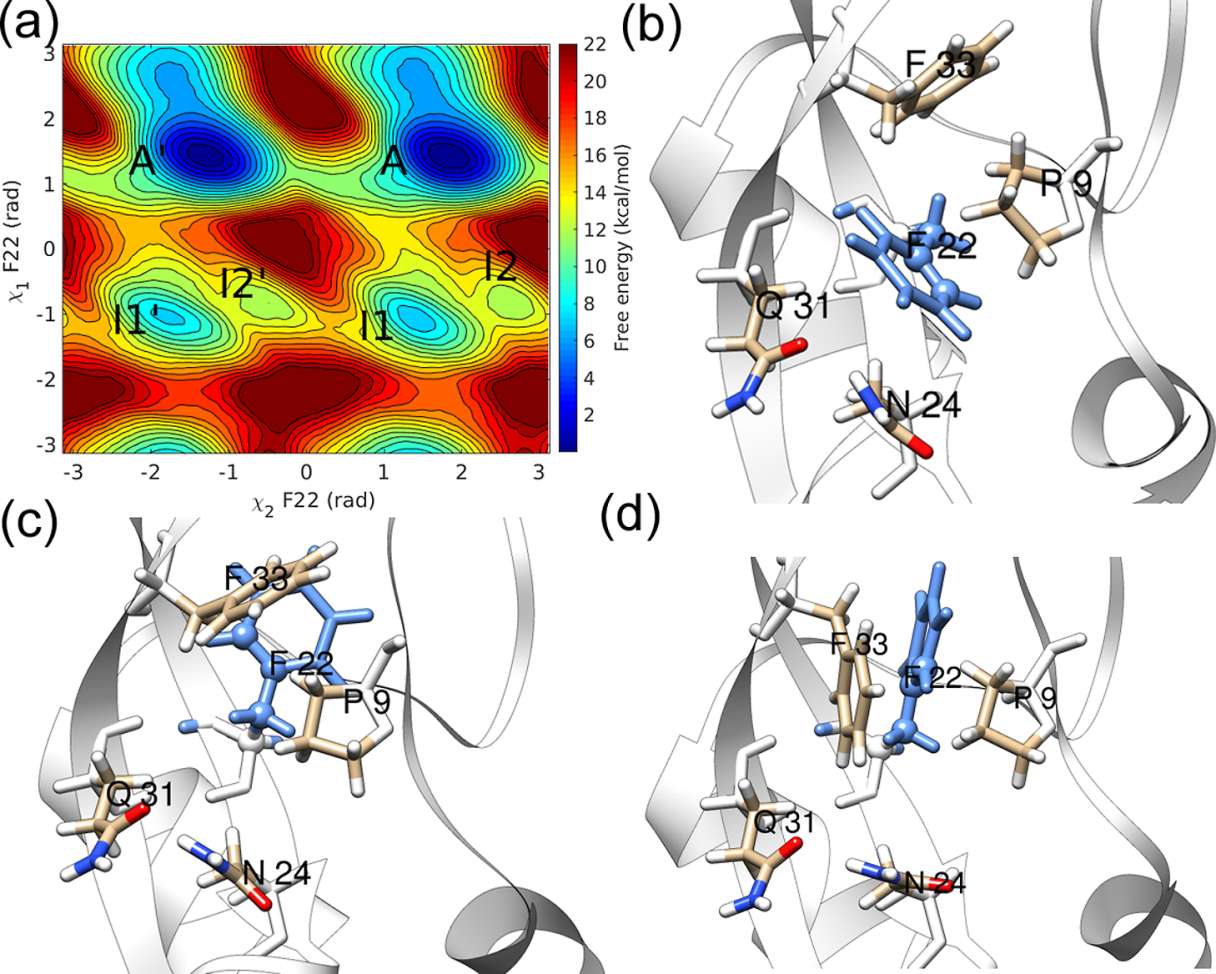
(a) Free energy of residue F22 (blue) along χ_2_–χ_1_ and representative structure from (b) minimum A, (c) minimum I1, and (d) minimum I2.

Figure 6 shows the χ_2_–χ_1_ free-energy surface for F22, along with representative structures. All the InMetaD simulations followed a direct A → A′ transition pathway, as a high barrier exists along χ_1_ to visit intermediate states. A fitted *t*_flip_ of 2.41 × 10^–2^ s was observed for the direct transition. The direct flip mechanism suggests that residue F22 needs minimal fluctuations relative to the backbone for ring flipping, and it might not be necessary to span the whole χ_1_ torsion range, as observed in WTMetaD simulations.

State I1 occurs due to changes in the χ_1_ value and a transient increase of the P9-F33 distance. In state I2, there is no stacking of F22 over F33, but these residues align approximately parallel to each other. During a long 1.6 μs WTMetaD simulation of F22, we observed a single ring flip event of residue F33 (Figure S25 in the Supporting Information). The KS-test results for residue F22 are reported in Figures S26 and S27 in the Supporting Information.

Five InMetaD simulations in each case of (a) *A* → *I*1 pathway, (b) *I*1 → *A* pathway, and (c) *I*1 → *I*1′ pathway were performed. In these simulations, visits to other stable states were avoided using repulsive wall bias^24^ using *k* and *s*_0_ values mentioned in Table S5 in the Supporting Information. The results (Table S6 in the Supporting Information) and details of these simulations are reported in Appendix II in the Supporting Information. The state-to-state transition rate matrix is reported in Table S7 in the Supporting Information. The dominant eigenvalue of 83 s^–1^ was estimated by diagonalizing this transition matrix.

#### Residue Y23

Residue Y23 is one of the slowest-flipping residues. First, we performed χ_2_–χ_1_ biased WTMetaD simulations; however, we observed a lack of convergence (Figures S33 and S34 in the Supporting Information). Visual observation of the WTMetaD simulation indicated a crowding effect of neighboring C_α_ atoms. Thus, a contact map was used as a complementary CV to obtain a converged free-energy landscape.

The dynamics of ring Y23 were studied using the χ_2_ torsion and a contact map of C_α_ atoms surrounding the ring (Figure S28 in the Supporting Information). The convergence of the WTMetaD simulations and the KS test validation of the InMetaD simulations assured the choice of CMAP as a CV complementary to χ_2_.

**Figure 7:**
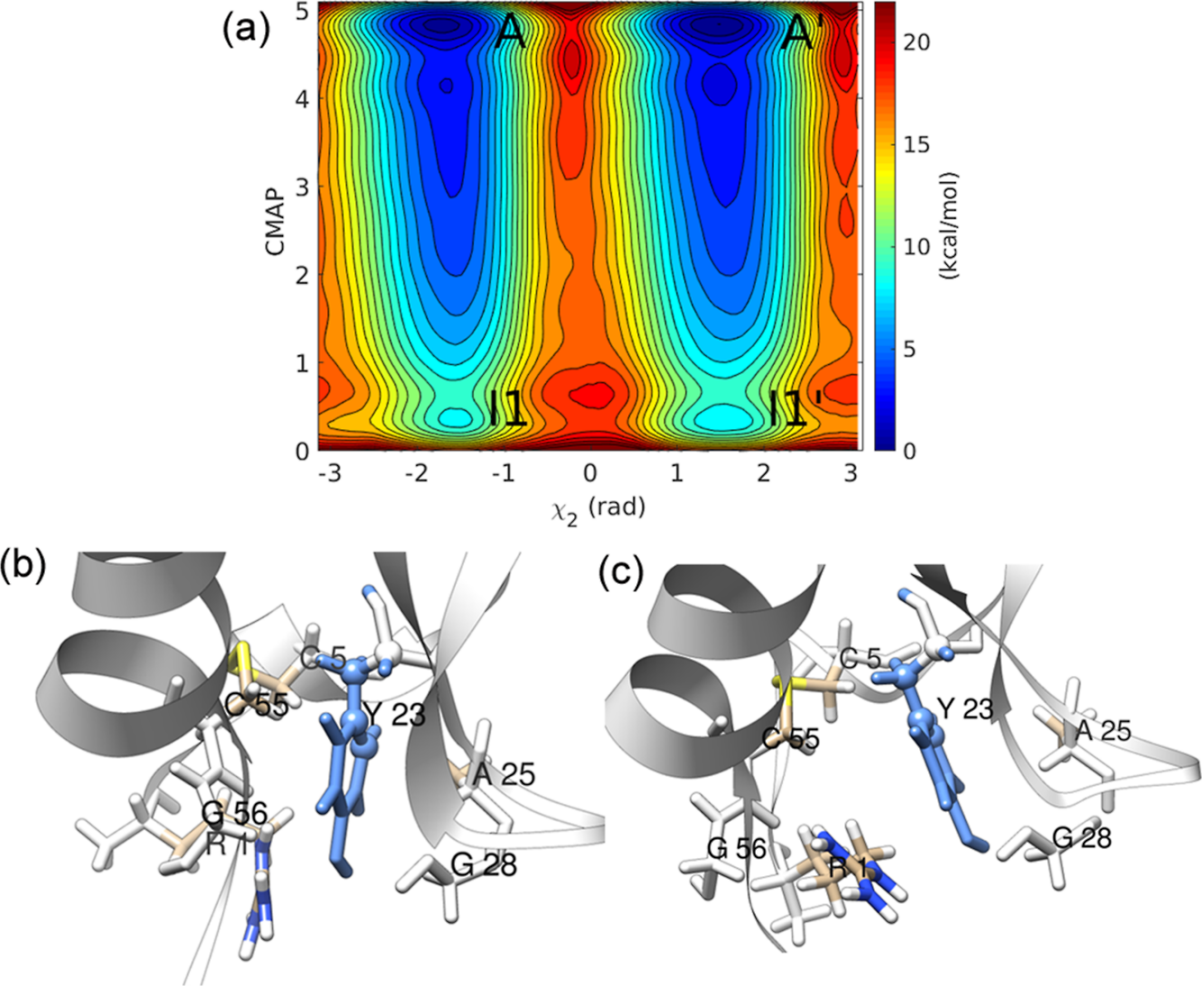
Free energy of (a) residue Y23 along χ_2_-CMAP, and representative structure from (b) minimum A and (c) minimum I1.

The transition is possible at CMAP values between 1 and 4 (Figure 7), indicating that a small change in the equilibrium distances of C_α_ atoms is sufficient to provide the necessary volume for ring flipping. Thus, a transient excited state could exist during ring flipping, in which the surrounding atoms are not significantly displaced. We performed 60 InMetaD simulations to obtain reliable statistics and observed a *t*_flip_ of 0.98 s (see Figures S29 and S30 in the Supporting Information).

Residue Y23 is located near two disulfide bridges defined by residues C5–C55 and C30–C51. The aromatic side chain of Y23 infrequently interacts with C_α_, S_γ_, and N atoms of residue C30 and backbone C and O atoms of residue C5. However, no significant changes in the values of χ_2_, χ_1_, and χ_3_ torsions of the 5–55 and 30–51 disulfide bridges were observed (Figure S31 in the Supporting Information). Transient hydrogen bonds between residue Y23 and residues R1, G56, and D3 were observed (Figure S32 in the Supporting Information). The fluctuations in the flexible residue R1 also transiently contributed to the cavity expansion due to interactions with residue G56. Both the χ_2_–χ_1_ and the χ_2_-CMAP InMetaD simulations provided similar *t*_flip_ values (0.61 and 0.98 s, respectively).

Karplus and Gelin^42^ theoretically studied aromatic ring flipping for Y10, Y21, Y23, and Y35 residues of BPTI by varying the χ_1_ and χ_2_ torsions. The study concluded that a few strong nonbonded interactions with surrounding atoms lead to a specific ring flipping rate constant (*k*_flip_) for each residue. In the DESRES simulation, the ring flipping rate was not correlated with the density of heavy atoms within 4 Å of aromatic ring atoms at δ and ε positions.^19^ This lack of correlation suggests an insignificant displacement of atoms surrounding the aromatic residue, contrary to the well-accepted cavity model.^12^ We speculate that small displacements of a few surrounding atoms are sufficient to allow ring flipping. The results for residue Y23 in this work support this hypothesis.

#### Residue Y35

**Figure 8:**
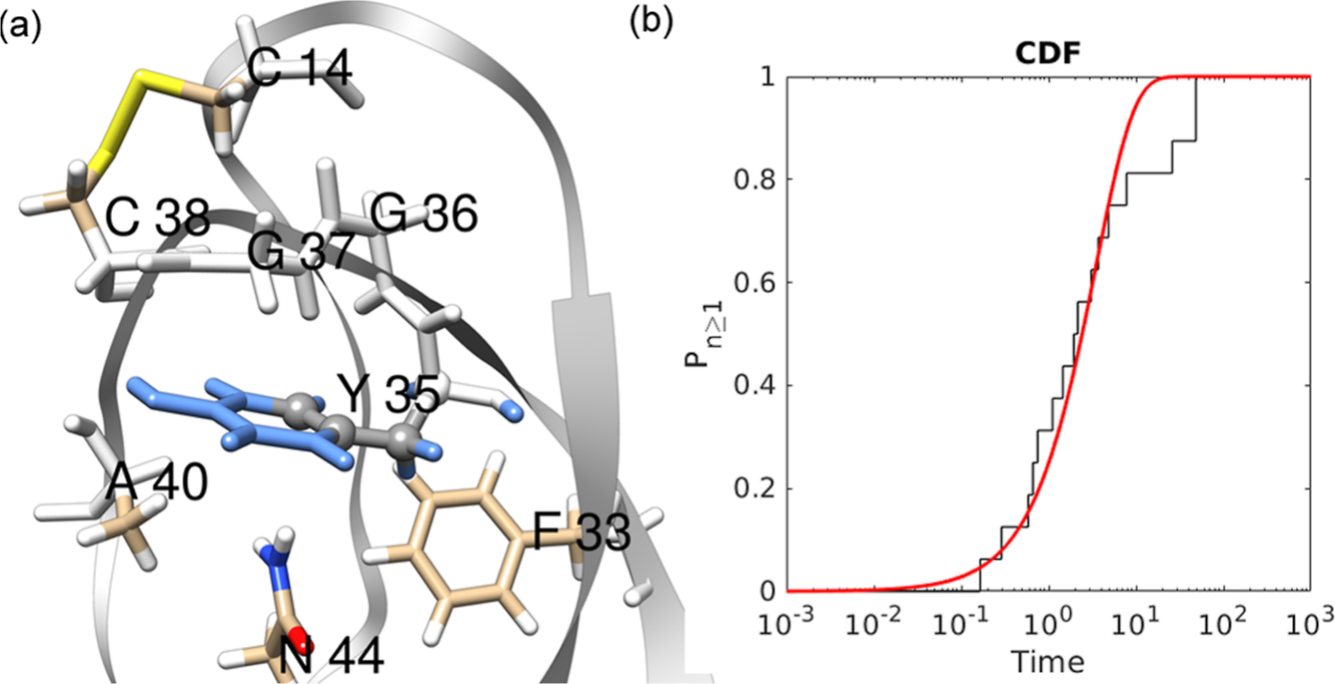
(a) Representative structure of residue Y35 in the native state, (b) Poisson fit to CDF of transition timescales obtained from χ_2_–χ_1_ InMetaD simulations (*p*-value = 0.58 and μ ln 2/*t*_m_ = 3.21).

Residue Y35 is a slow-flipping residue in BPTI and is one of the most studied aromatic side chains by previous^10,25,50−52^ theoretical studies. It is located inside the protein core, close to the C14–C38 disulfide bond (Figure 8), and near the initial part of the loop region formed by residues G36-A40. WTMetaD simulations along χ_2_–χ_1_ (1.6 μs simulation time) and χ_2_ (0.7 μs simulation time) for residue Y35 lacked convergence (Figures S8 and S9 in the Supporting Information).

The lack of converged free-energy profiles for Y35 with χ_2_ as well as χ_2_–χ_1_ biased metadynamics simulations indicates a missing orthogonal degree of freedom and probably a complex ring flipping mechanism. Thus, we speculate that more than two CVs might be needed to understand the flipping of the Y35 aromatic side chain.

The reweighted distribution of side chain torsions χ_1_ of C14 and C38 residues indicates that this simulation sampled states other than M (Figure S35 in the Supporting Information). Thus, the loop region’s conformations due to C14–C38 disulfide bond motions could affect the flipping rate.^19^ We performed 16 InMetaD simulations, assuming χ_2_–χ_1_ could provide rates in the major state M1. A *t*_flip_ of 3.4 s was obtained, indicating Y35 as a slow-flipping residue.

The disulfide bond isomerization could likely be associated with the non-convergence of the χ_2_–χ_1_ metadynamics simulations. Even in a 1 ms-long trajectory, multiple flipping rates of residue Y35 were reported based on C14–C38 disulfide bond isomerization.^19^ Thus, we have also performed WTMetaD simulations with χ_2_ and χ_1_ of Y35, together with the χ_3_ dihedral of the C14–C38 disulfide bond. This simulation demonstrated a 6 kcal/mol free-energy barrier for Y35 ring flipping, as the Y35 aromatic side chain is irreversibly expelled out of the protein core and freely rotates in the solvent-exposed state (Figure S36 in the Supporting Information).

### Recrossing Events during F22 and F33 Ring Flipping

**Figure 9:**
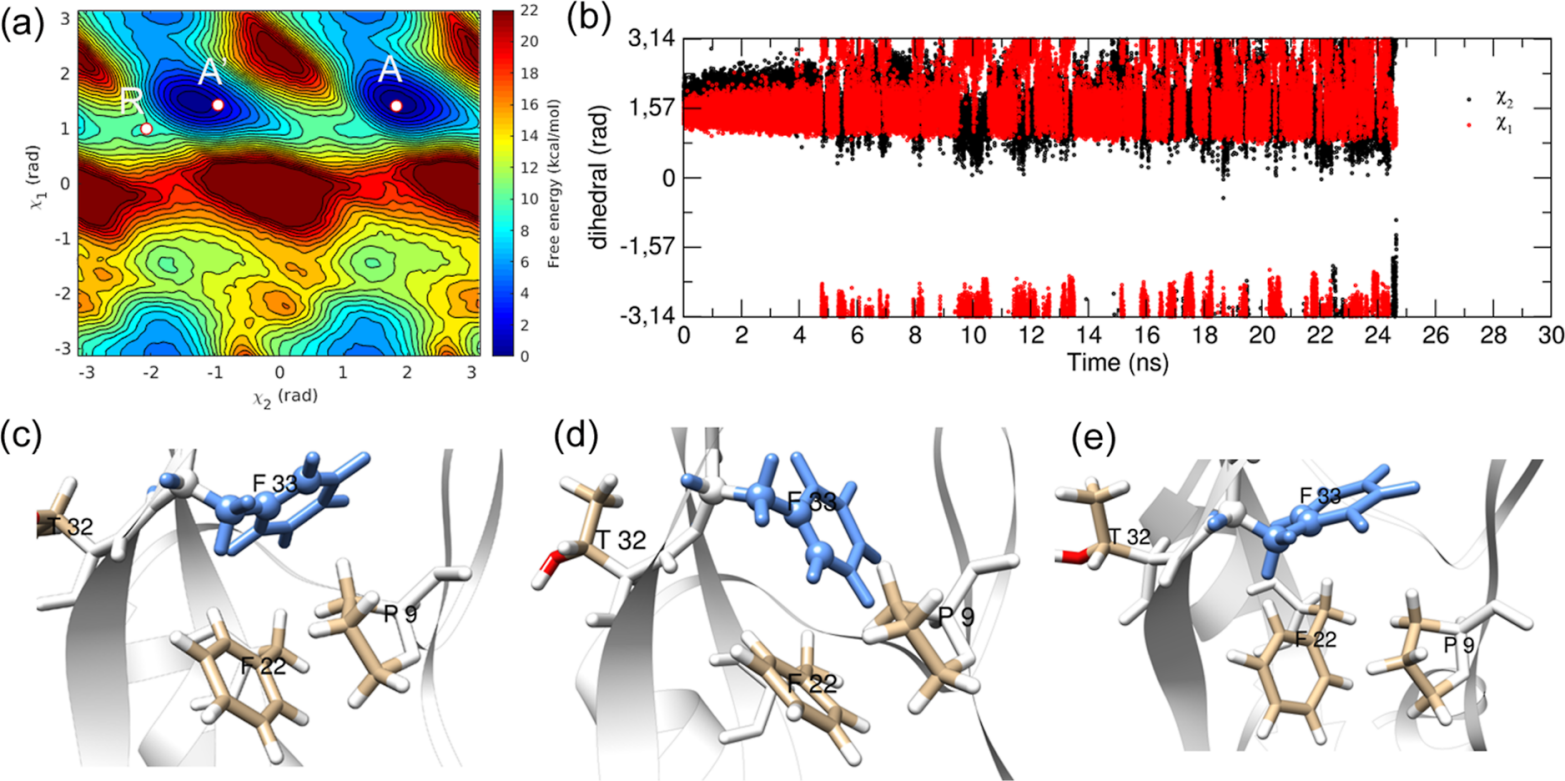
Representative InMetaD trajectory showing the recrossing event in the F33 ring flipping. (a) the starting state ***A***, flipped state ***A***′, and point of recrossing ***R*** on the free-energy surface of χ_2_–χ_1_, (b) time evolution of χ_2_ and χ_1_ torsion angles, (c) orientation of F33 in the native state (state *A*, χ_2_ = 1.83 rad, χ_1_ = 1.41 rad), (d) structure at 22538 ps (state *R*, χ_2_ = −2.06 rad, χ_1_ = 0.99 rad), (e) flipped state at 24643 ps (state *A*′, χ_2_ = -0.95 rad, χ_1_ = 1.42 rad).

The χ_2_–χ_1_ biased InMetaD simulations of residues F22 and F33 demonstrated recrossing events. In Figure 9, we present one such trajectory of residue F33 to understand the structural details of recrossing. In this trajectory, the first attempt of ring flipping occurred around 22,538 ps (Figure 9b), when the χ_2_ value reached −2.06 rad while maintaining positive χ_1_ values (state R). The ring rotates back to positive χ_2_ values after this state and keeps sampling in the initial region with a few *A–I*1 transitions.

TS theory^53,54^ (TST) assumes that the trajectories cross the barrier region (which divides the reactant and product states) and continue to the product state. The TST-based transition rate (*k*_TST_) can be calculated from the free energy of activation (Δ*G*^*‡*^) using the Eyring eq 10.
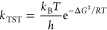
10where *k*_B_ is Boltzmann’s constant, *T* is the temperature, *h* is Planck’s constant, and *R* is the gas constant. The TST-based rate due to recrossing events is corrected using the transmission coefficient (κ)
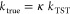
11The origin^54,55^ of recrossing events could be associated with the efficiency of CVs to describe the TS or with strong coupling between system coordinates undergoing transition and the surrounding environment. However, it is not very easy to define the precise TS. Thus, according to variational TST,^54,55^ an optimized reaction coordinate that minimizes recrossing events should be defined to understand dynamics. For residue F22, a barrier of 12 kcal/mol exists along the direct flipping pathway, thus estimating the ring flipping rate, *k*_TST_ = 1.13 × 10^4^ s^–1^, according to eq 10. If CVs χ_2_ and χ_1_ are speculated to capture the dynamics of F22 ring flipping correctly so that the true rate can be equated with the one obtained from InMetaD simulations, eq 11 gives κ = 3.67 × 10^–3^.

Northrup et al.^25^ predicted a transmission coefficient of 0.22 for residue Y35 in BPTI with theoretical calculations of reactive flux using umbrella sampling simulations. Thus, for χ_2_–χ_1_ biased InMetaD simulations of F22 ring flips, we hypothesize that an unusually low transmission coefficient κ(3.67 × 10^–3^) value might be attributed to the choice of CVs. We performed 12 InMetaD simulations in each residue F22 and F33 case using reaction coordinates optimized using a variational approach (VAC-MetaD) to verify this assumption.^41^ This approach allows a linear combination of multiple CVs and better describes transition and stable states. We included the distance between residues P9 and F33, χ_1_ and χ_2_ torsions of residues F22 and F33 to build VAC-optimized CVs (VAC-CVs). The computational details and results of these simulations are reported in the SI (Appendix I in the Supporting Information).

With VAC-CVs, *t*_flip_ values of 0.09 s (*p*-value = 0.49) and 8.64 × 10^–5^ s (*p*-value = 0.70) were obtained for residues F22 and F33, respectively (Figure S48 in the Supporting Information). Thus, even with optimized CVs, residue F22 gave a low κ value. Furthermore, we also observed recrossing events in a few F22 and F33 VAC-CV-based simulations. Thus, further analysis is needed to understand the origin of such a low transmission coefficient and the corresponding frictional effects. A recrossing event was also observed for c36-based InMetaD simulations of F22.

### Limitations of 1D χ_2_ InMetaD Simulations

We performed InMetaD simulations biasing χ_2_ as a standalone CV to understand its limitations and accuracy in estimating *t*_flip_ values. The details of metadynamics parameters and the outcome of χ_2_ biased InMetaD runs are reported in Table 4. KS test results for all residues are reported in Figures S41 and S42 in the Supporting Information (InMetaD simulations were not performed for residue Y35, as WTMetaD simulations biasing χ_2_ of Y35 lacked frequent ring flip events, as shown in Figure S9 in the Supporting Information).

**Table 4:** Results of χ_2_ Biased InMetaD Simulations; the Derived Ring Flip Rate () at 300 K, the Number of Simulations (*N*), the Ratio of the Average (μ) to the Median of the Poisson Distribution, the *p*-Value from the KS Test, and the Simulation Parameters Hill Height (*w*, kJ mol^–1^), Bias Factor (γ), and Frequency of Hill Addition (ν, ps)

residue	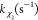	*N*	(μ ln 2)/*t*_m_	*p*-value	γ	*w*	ν
F4	3.44 × 10^4^	12	2.98	0.86	15	1.2	12
Y10	5.88 × 10^2^	40	1.79	0.21	10	0.6	18
Y21	1.92 × 10^2^	40	1.32	0.68	10	0.6	18
F22	3.65	20	2.94	0.45	8	1.2	12
Y23	4.0 × 10^–2^	12	3.71	0.02	12	1.2	12
F33	3.4 × 10^4^	15	3.46	0.90	12	0.6	10
F45	1.2 × 10^–1^	40	5.39	0.00	10	0.6	18

Except for Y21, the χ_2_ biased WTMetaD simulations lacked convergence for all residues. An example is shown in Figure S43 in the Supporting Information for residue Y10. The slow diffusion of χ_2_ and χ_1_ of residue Y10 in χ_2_ biased WTMetaD simulation suggests that biasing of both CVs is necessary. InMetaD simulations along χ_2_ for residue Y10 yielded *t*_flip_ values two orders of magnitude slower than χ_2_–χ_1_ InMetaD.

We observed that most of these trajectories had a direct transition from state *A* to *A*′ with no significant fluctuations along the χ_1_ torsion. This result shows that biased CVs direct the reactive flux along pathways specific to them and could provide different timescales. In particular, *t*_flip_ could be overestimated if diffusion along other relevant degrees of freedom is neglected in InMetaD. It is noteworthy that both χ_2_ and χ_2_–χ_1_ biased InMetaD simulations of residue Y10 passed the KS-test and had a low (μ ln 2)/*t*_*m*_ ratio. Thus, missing descriptors of the process could affect the metadynamics-derived *t*_flip_ values even if the two-sample KS test is passed. Theoretically, multiple CVs are needed to capture all metastable states on the free-energy landscape. Thus, recent machine learning-based methods to define a combination of multiple CVs could be specifically useful to obtain reliable rates with InMetaD.

For residue F22, InMetaD simulations with the χ_2_ torsion passed the KS test but predicted *t*_flip_ an order of magnitude slower than the χ_2_–χ_1_ InMetaD simulations. Interestingly, both types of simulations (χ_2_ and χ_2_–χ_1_ InMetaD) gave much higher *t*_flip_ values than observed in the DESRES trajectory. For residue F45, the distribution of τ_*i*_ values from the χ_2_ InMetaD simulations (Figure S42 in the Supporting Information) indicated deviation (*p*-value of 0.00) from the ideal Poisson distribution (although the *t*_flip_ value obtained from these simulations is comparable with the value obtained from χ_2_–χ_1_ InMetaD simulations). This exemplifies that apart from CVs that discriminate stable states, CVs which promote diffusion between the states are needed for reliable estimation of dynamics from metadynamics.

### CHARMM Simulations

**Figure 10:**
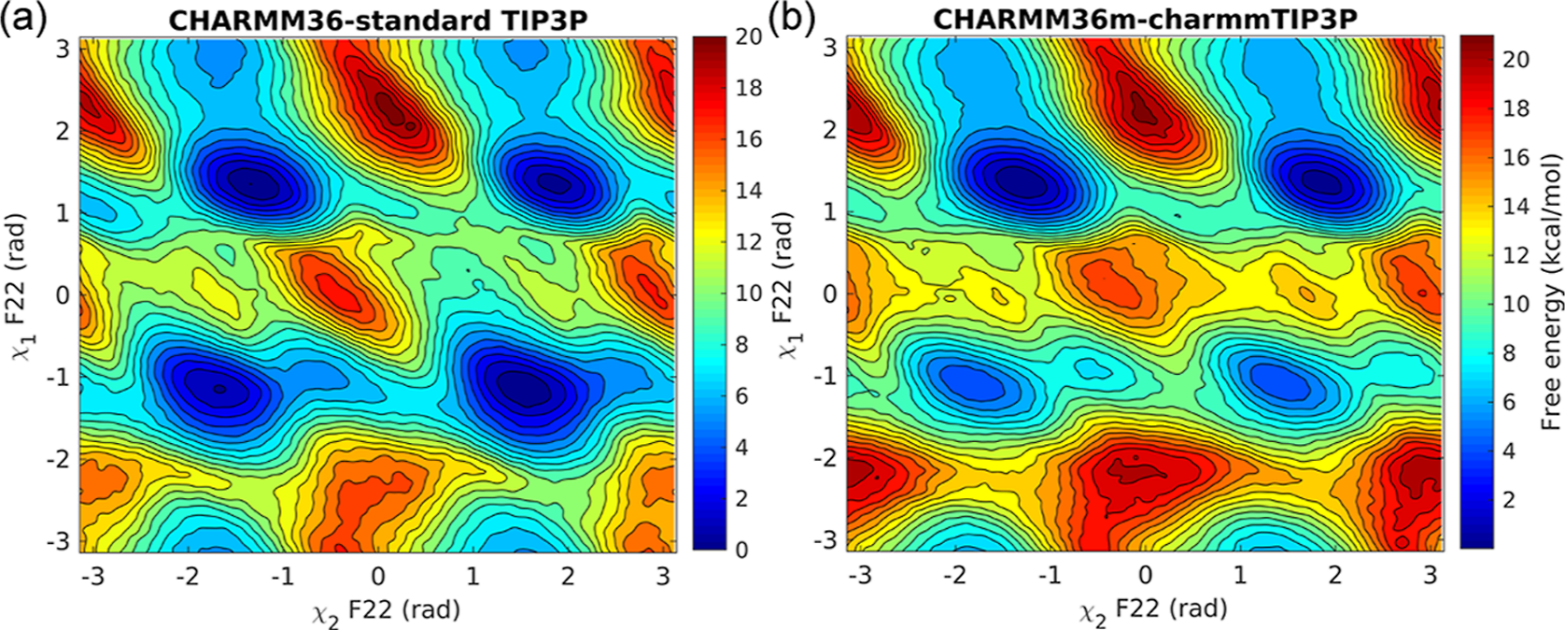
(a) Free-energy landscape of residue F22 along χ_2_–χ_1_ CVs with (a) CHARMM36 and TIP3P water model and (b) CHARMM36m and modified TIP3P water model.

We have performed metadynamics simulations using two CHARMM36 force field variants, c36, and c36m, as described in the method section, for residue F22 with χ_2_ and χ_1_ CVs. The corresponding free-energy surfaces are shown in Figure 10. The free-energy barriers along χ_1_ are 3 kcal/mol lower compared to the AMBER ff14SB force field. The convergence behavior of these simulations is reported in the Supporting Information (Figures S44 and S45).

The ring flip timescales were estimated for residues F22 (16 simulations) and F45 (12 simulations) modeled with the c36 force field. For residues F22 and F45, *t*_flip_ values of 5.45 × 10^–4^ s (*k*_flip_ = 1.83 × 10^3^ s^–1^, *p*-value = 0.69) and 1.78 × 10^–1^ s (*k*_flip_ = 5.61 s^–1^, *p-*value = 0.97) were observed, respectively, predicting slow-flipping residues (Figure S46 in the Supporting Information), but faster by 1-2 orders of magnitude than the AMBER ff14SB force field. The barrier of 9.0 kcal/mol for F22 suggests *k*_TST_ = 1.74 × 10^6^ s^–1^, thus giving (again) a low κ value of 1.04 × 10^–3^ for the c36-based F22 InMetaD simulations. These results demonstrate the sensitivity of ring flip events to the force field parameterization. Ring flip events are local in nature, and thus, reparameterization of aromatic side chain torsion potentials with QM methods, such as coupled cluster calculations, is crucial for ensuring an accurate description of the rotational energy barrier and quantitative agreement with experiments. It would also be interesting to explore other combinations of force fields, such as the recently proposed AMBER FF19SB^56^ force field with the OPC water model. However, both of these approaches are beyond the scope of the present work which focuses on sampling methods.

## Conclusions

In the present work, we have described a metadynamics-based approach to calculating the ring flipping rate and mechanism of eight individual aromatic rings in BPTI. We utilized the InMetaD method to obtain the first passage times (τ_*i*_) to estimate *t*_flip_. This approach successfully distinguished between experimentally categorized “slow” and “fast” residues with an appropriate choice of CVs. Our InMetaD simulations categorized residues F4, F33, and Y10 as fast-flipping residues and Y21, F22, Y23, F45, and Y35 as slow-flipping residues. Residues Y35, Y23, and F45 exhibited timescales up to seconds, significantly slower than a millisecond brute-force MD simulation. The rank order observed for ring flips from slow to fast is F45 < Y23, Y35 < Y21, F22 < Y10 < F4, F33. Indeed, NMR measurements^3^ and 1 ms-long simulation^19^ observed residues F33 and F4 as fast-flipping residues. For reference, we also investigated the correlation between solvent-accessible surface area (SASA) of aromatic side chains and experimental ring flipping rates *k*_exp_; however, no correlation was observed, in agreement with a previous study (Figure S39 in the Supporting Information).^57^

The free-energy surface of solvent-exposed residues (F4, Y10) and buried residues (F22, F33, and Y35) indicated multiple ring flipping pathways. The circular correlation coefficient between χ_1_ and χ_2_ dihedrals of eight aromatic residues obtained from the 1 ms-long trajectory indicates no correlated motions between these residues (Figure S40 in the Supporting Information).^58^ The side chain aromatic ring of residues Y23 and F45 flips around seconds; these time scales are still beyond the scope of present MD simulations. The InMetaD simulations of residues F33 and F22 indicate friction effects during barrier-crossing events. Further investigation is needed to understand the origin of these observations.

In conclusion, this study illustrates that InMetaD can be applied to understand motion occurring on timescales of seconds, as was previously demonstrated by drug-unbinding studies.^48,59,60^ It also exposes the complexity of the ring-flipping process in globular proteins.

## Supporting Information Available

Convergence of WTMetaD simulations, results of χ_2_ InMetaD simulations, distribution of side chain torsions of a C14–C38 disulfide bond, SASA results, circular cross-correlation of χ_2_ and χ_1_ dihedral values obtained from 1 ms-long D. E. Shaw Research (DESRES) trajectory, details of state-to-state transitions of residue F22, and details of VAC-metadynamics simulations (PDF)
