# Supplementary material for: Free-Energy Landscape and Rate Estimation of the Aromatic Ring Flips in Basic Pancreatic Trypsin Inhibitors Using Metadynamics
Source: J Chem Theory Comput. 2023 Sep 12;19(19):6605–18. doi: 10.1021/acs.jctc.3c00460 (PMC10569046; doi:10.1021/acs.jctc.3c00460)
Supplement: Supplementary file 1 — ct3c00460_si_001.pdf [file ct3c00460_si_001.pdf]

Supporting Information

Free Energy Landscape and Rate Estimation of the Aromatic Ring Flips in Basic Pancreatic Trypsin Inhibitor Using Metadynamics

Mandar Kulkarni* and Pär Söderhjelm*

Division of Biophysical Chemistry, Lund University, Chemical Center, 22100 Lund, Sweden

*E-mail: mandar.kulkarni.chem@gmail.com; par.soderhjelm@bpc.lu.se

Table S1. The values of r_0 and d_0 for C_α atom pairs in CMAP (Equation 2)

	C_α atom pair ($\boldsymbol{ij}$)				**Figure with atom names**
Sr. no.	$C_\alpha(i)$	$C_\alpha(j)$	r_0 (nm)	d_0 (nm)	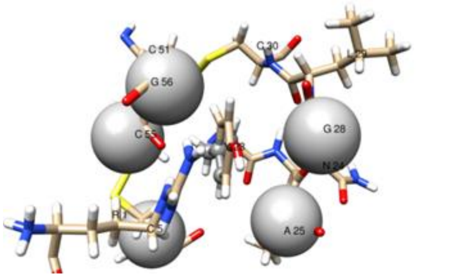
1	A25	G28	0.08	0.47	
2	G28	G56	0.10	0.77	
3	C55	A25	0.08	0.92	
4	C55	C5	0.03	0.58	
5	A25	C5	0.06	0.72	

Table S2. 95% CI[a] of transition timescales (t_{flip}) values

Residue	CVs	t_{flip} (s)	lower bound (s)	upper bound (s)
F4	χ_2, χ_1	2.80×10^{-5}	2.11×10^{-5}	3.57×10^{-5}
Y10	χ_2, χ_1	5.80×10^{-5}	4.38×10^{-5}	7.39×10^{-5}
Y21	χ_2	5.20×10^{-3}	3.93×10^{-3}	6.62×10^{-3}
F22	χ_2, χ_1	2.41×10^{-2}	1.82×10^{-2}	3.07×10^{-2}
F22 (c36)[d]	χ_2, χ_1	5.45×10^{-4}	3.42×10^{-4}	7.87×10^{-4}
Y23	χ_2, CMAP	9.81×10^{-1}	7.41×10^{-1}	1.25
F33	χ_2, χ_1	3.50×10^{-5}	2.64×10^{-5}	4.46×10^{-5}
Y35	χ_2, χ_1	3.49	2.63	4.44
F45	χ_2, χ_1	3.22	2.43	4.10
F45 (c36)[e]	χ_2, χ_1	1.78×10^{-1}	1.03×10^{-1}	2.70×10^{-1}

[a]See Python function (ci95) below to calculate 95% CI

```
import sci.stats
def ci95(N, t_flip):
    lower_bound = sci.stats.gamma.ppf(0.05, a=N,loc=0,scale=t_flip/N)
    upper_bound = sci.stats.gamma.ppf(0.95, a=N,loc=0,scale=t_flip/N)
    return ("lower bound: %0.2e , upper_bound: %0.2e" %(lower_bound, upper_bound))
```

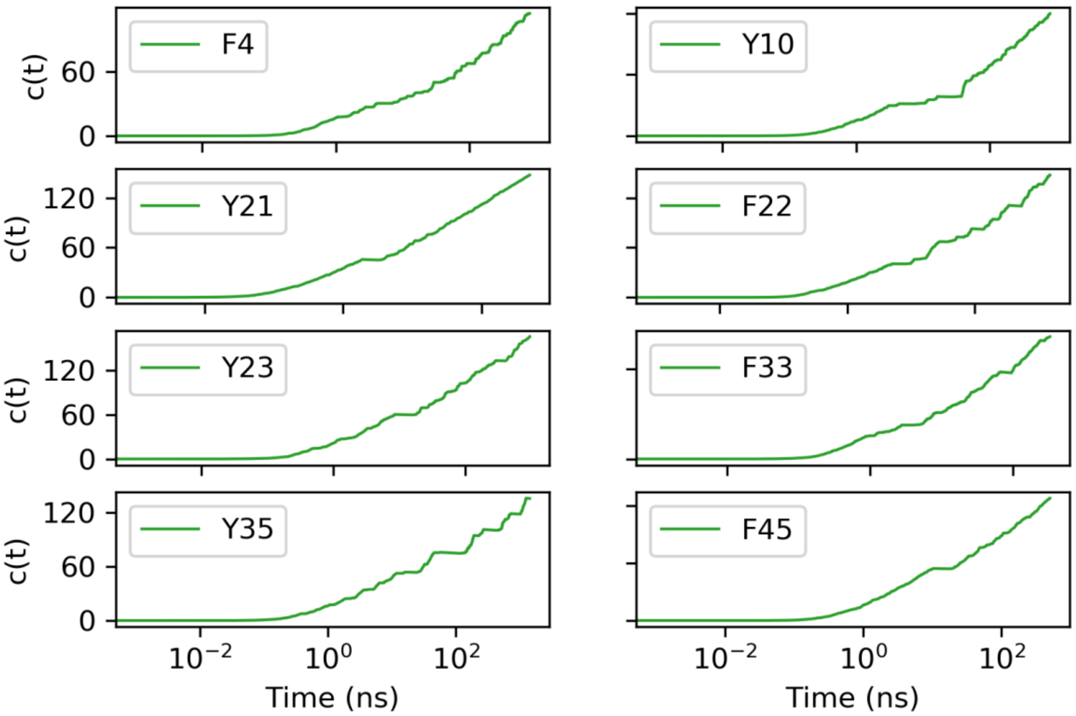

Figure S1. The bias offset c(t) obtained from reweighting converged simulations for each residue. Please note, residue Y35 $\chi_2 - \chi_1$ metadynamics simulation lacks convergence.

Simulation results:

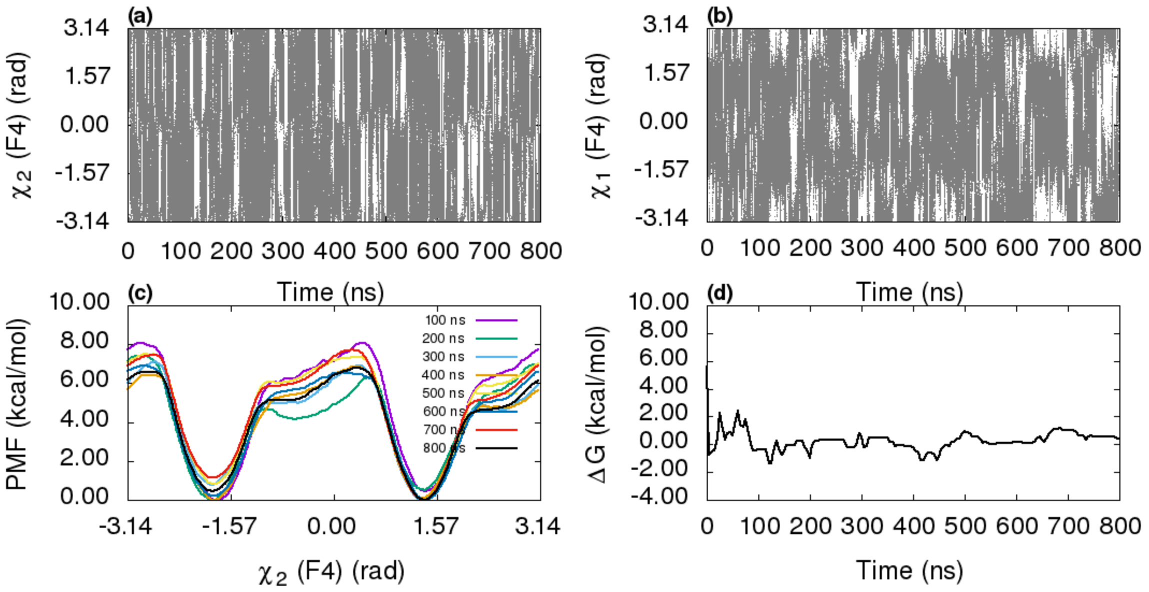

Figure S2. Diffusion of χ_2 and χ_1 during $\chi_2 - \chi_1$ metadynamics simulation of residue F4 is shown in figures (a) and (b), respectively. (c) Projection of free energy on χ_2 in time intervals of 100 ns. (d) The free energy difference between two symmetric states along χ_2.

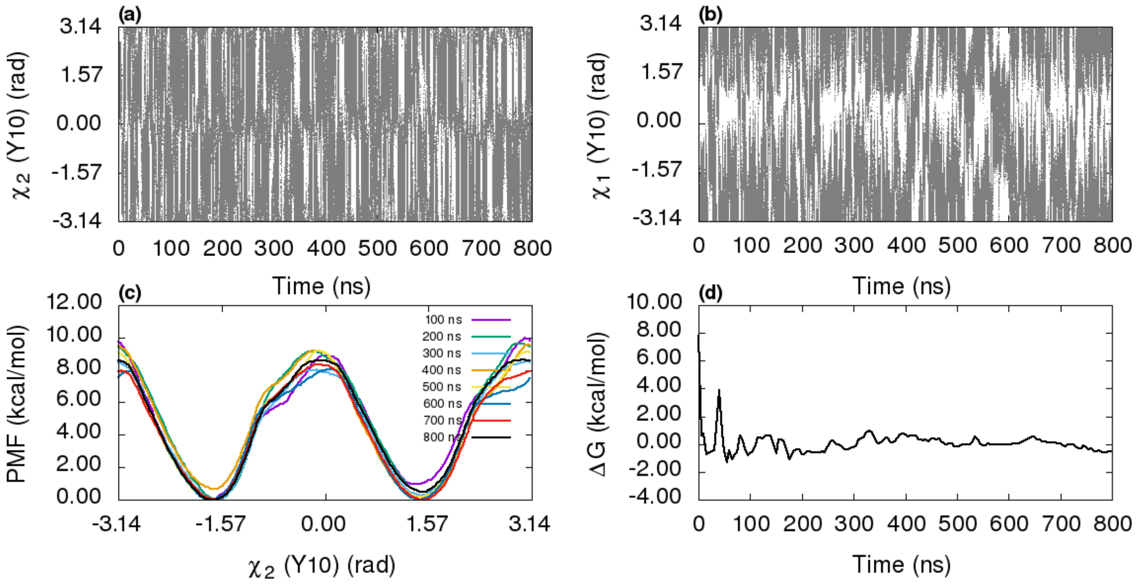

Figure S3. The time series of χ_2 and χ_1 during $\chi_2 - \chi_1$ metadynamics simulation showed in figures (a) and (b), respectively. (c) the projection of free energy on χ_2 in time intervals of 100 ns. (d) The free energy difference between two symmetric states along χ_2.

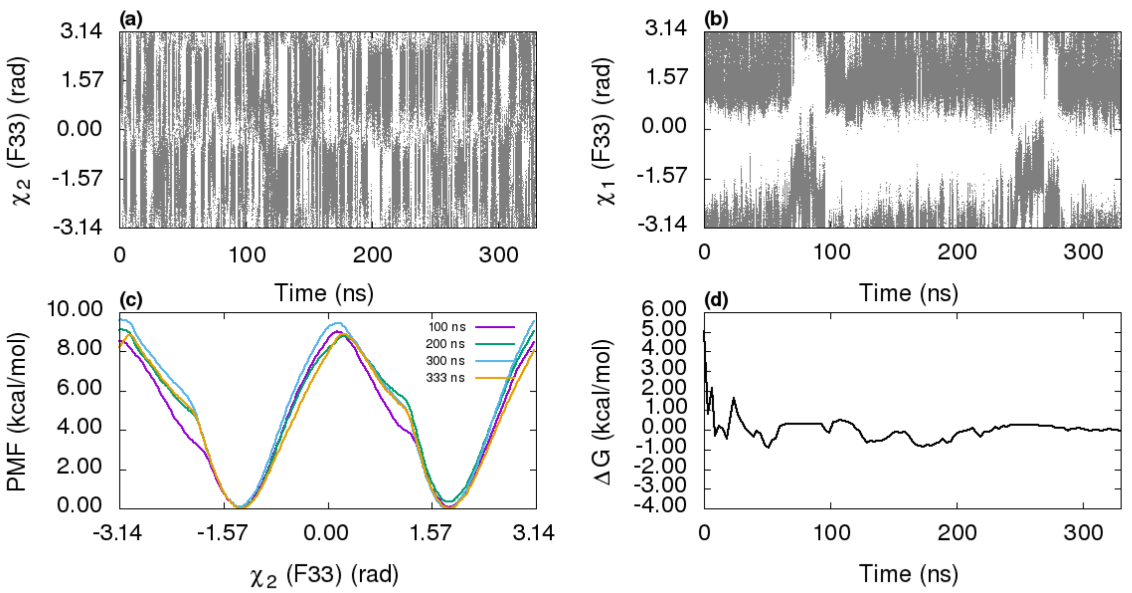

Figure S4. The diffusion of χ_2 and χ_1 variables during $\chi_2 - \chi_1$ metadynamics simulation of residue F33 (figures (a) and (b), respectively). (c) the projection of free energy on χ_2 in time intervals of 100 ns. (d) The free energy difference between two symmetric states along χ_2.

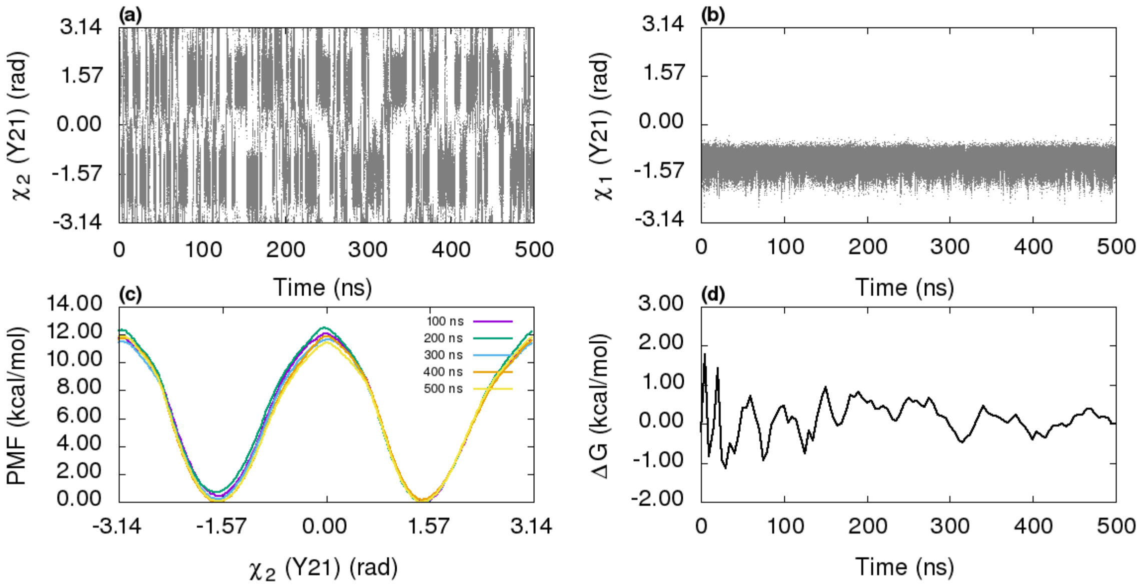

Figure S5. The diffusion of χ_2 and χ_1 collective variables during χ_2 metadynamics simulation of residue Y21 (figures (a) and (b), respectively). (c) the free energy along χ_2 in the time intervals of 100 ns. (d) The free energy difference between two symmetric states along χ_2.

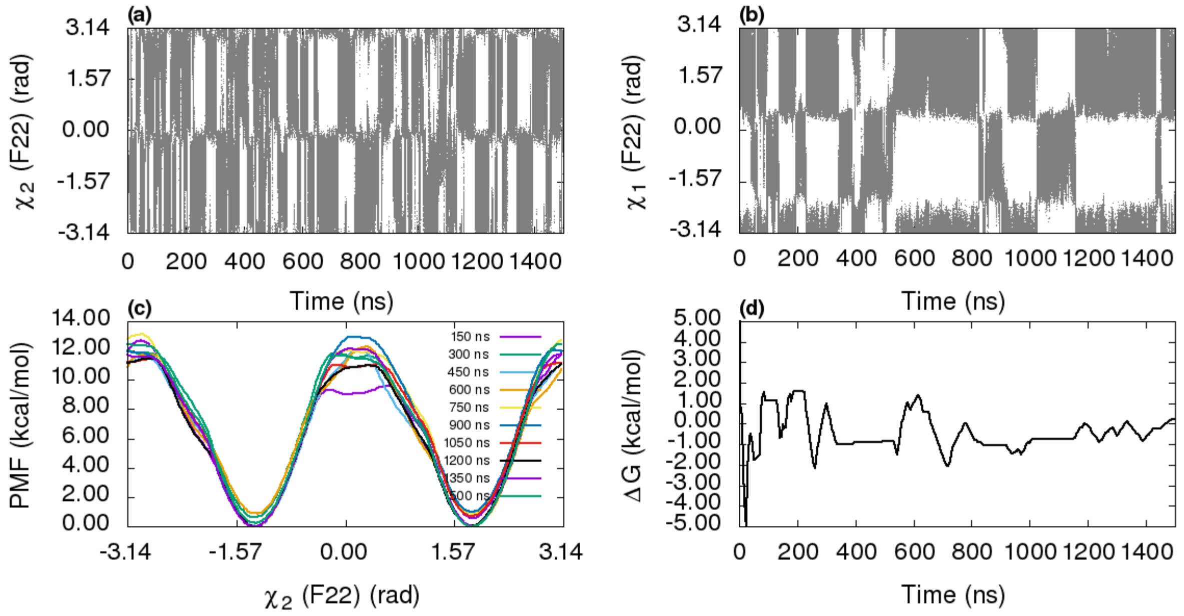

Figure S6. The time series of χ_2 and χ_1 during $\chi_2 - \chi_1$ metadynamics simulation of residue F22 showed in figures (a) and (b), respectively. (c) the projection of free energy on χ_2 in time intervals of 150 ns. (d) The free energy difference between two symmetric states along χ_2.

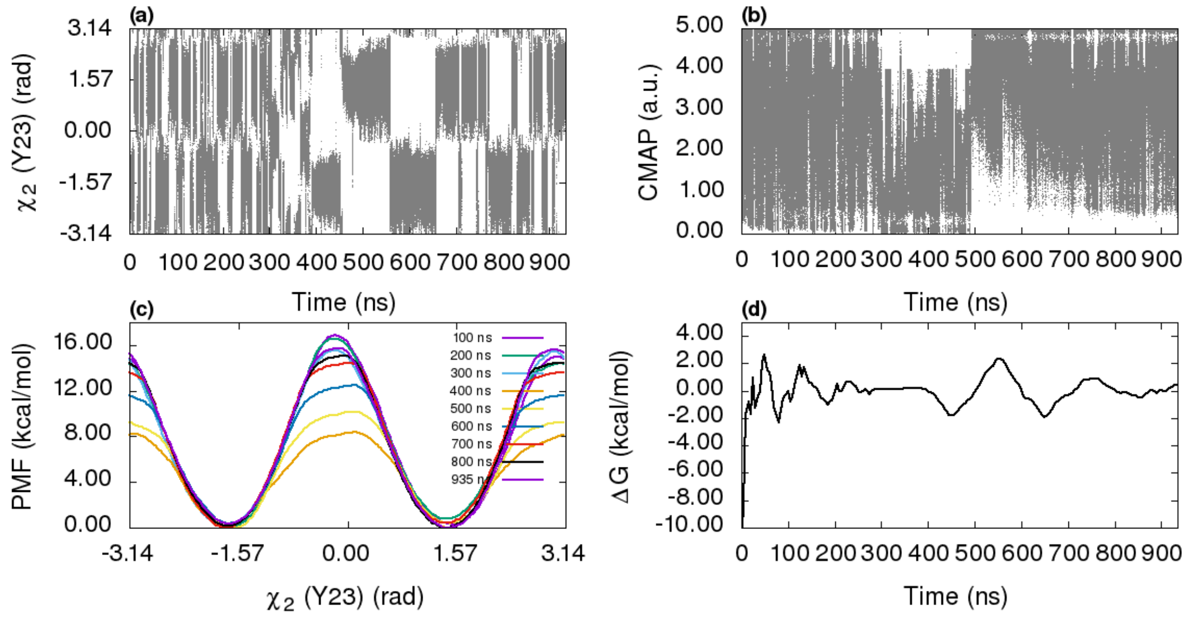

Figure S7. The time series of χ_2 and contact map (CMAP) collective variables during χ_2-CMAP metadynamics simulation of residue Y23 in figures (a) and (b), respectively. (c) the projection of free energy along χ_2 in time intervals of 100 ns. (d) the free energy difference between two symmetric states along χ_2.

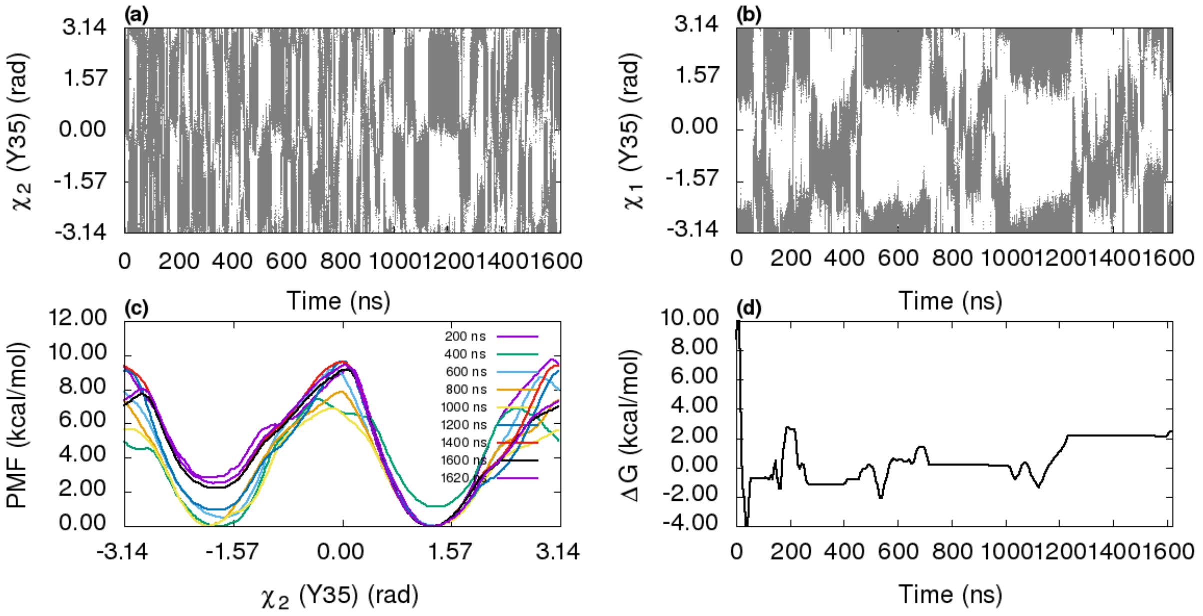

Figure S8. The time series of χ_2 and χ_1 during $\chi_2 - \chi_1$metadynamics simulation of residue Y35 in figures (a) and (b), respectively. (c) the projection of free energy on χ_2 in time intervals of 200 ns. (d) The free energy difference between two symmetric states along χ_2.

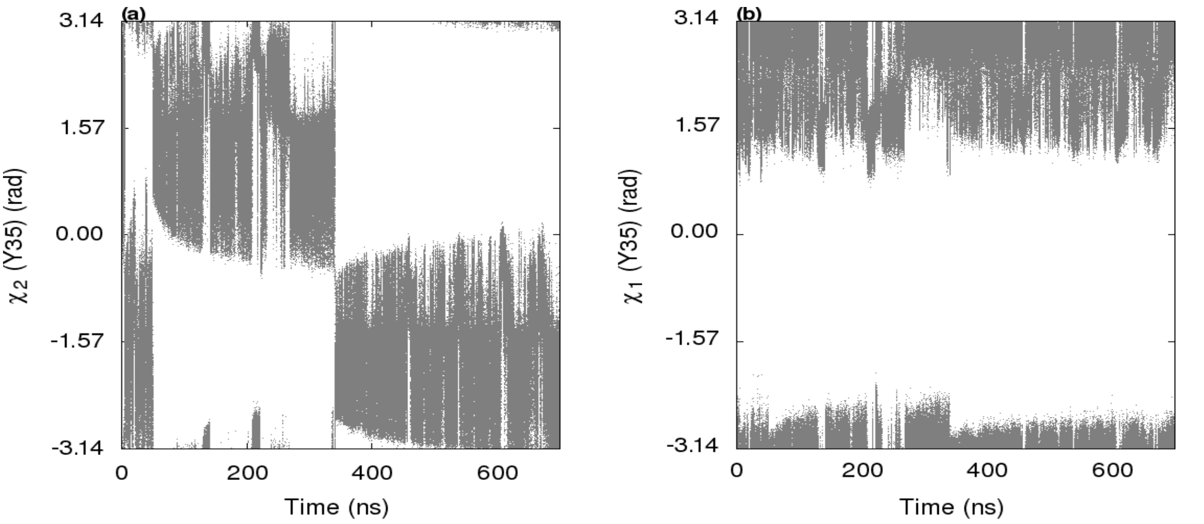

Figure S9. The time series of χ_2 and χ_1 during χ_2 metadynamics simulation of residue Y35 in figures (a) and (b), respectively.

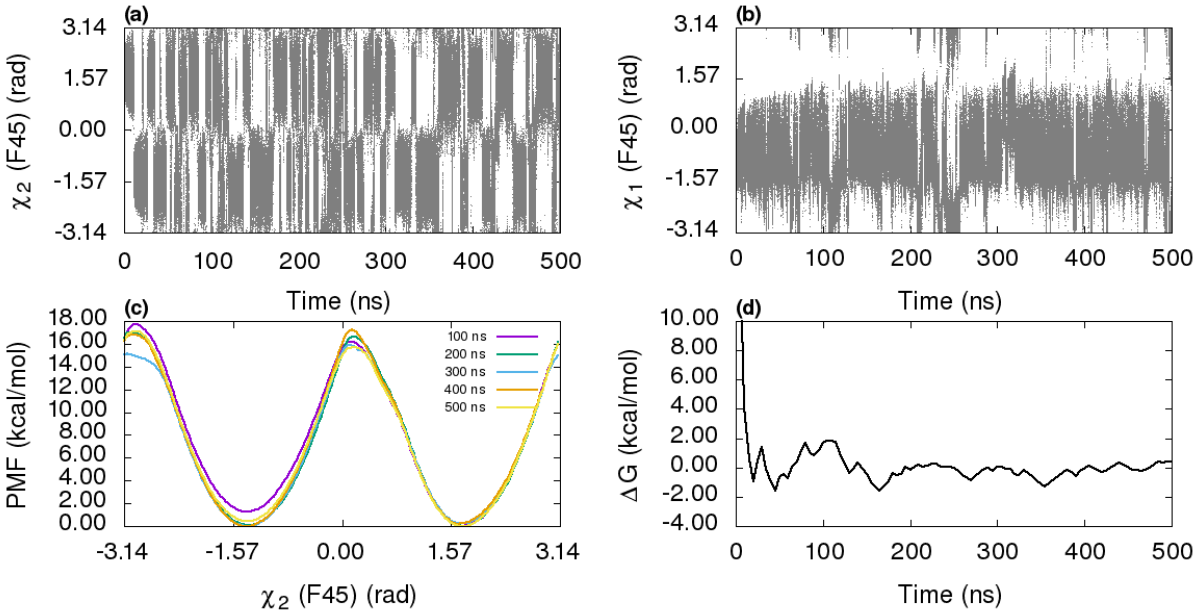

Figure S10. The diffusion of χ_2 and χ_1 during $\chi_2 - \chi_1$ metadynamics simulation of residue F45 in figures (a) and (b), respectively. (c) the projection of free energy on χ_2 in time intervals of 100 ns. (d) The free energy difference between two symmetric states along χ_2.

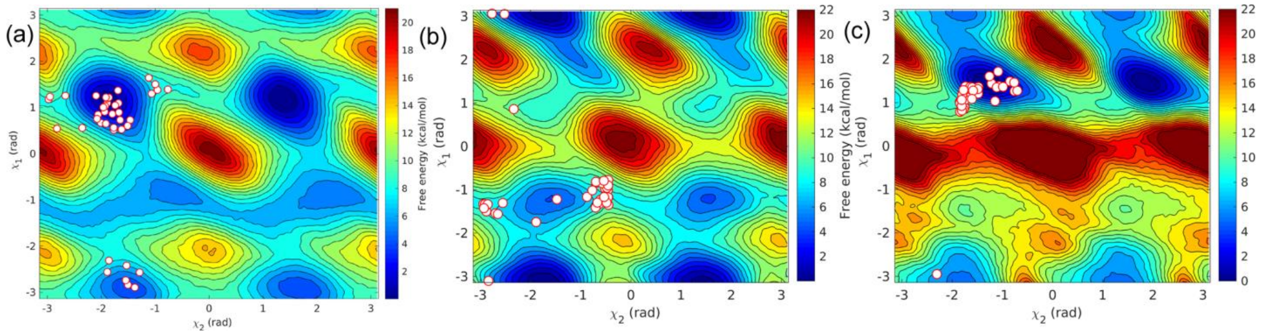

Figure S11. The distribution of trajectory endpoints after flipping for (a) residue F4, (b) residue Y10, and (c) residue F33 ($\chi_2 - \chi_1$metadynamics simulations).

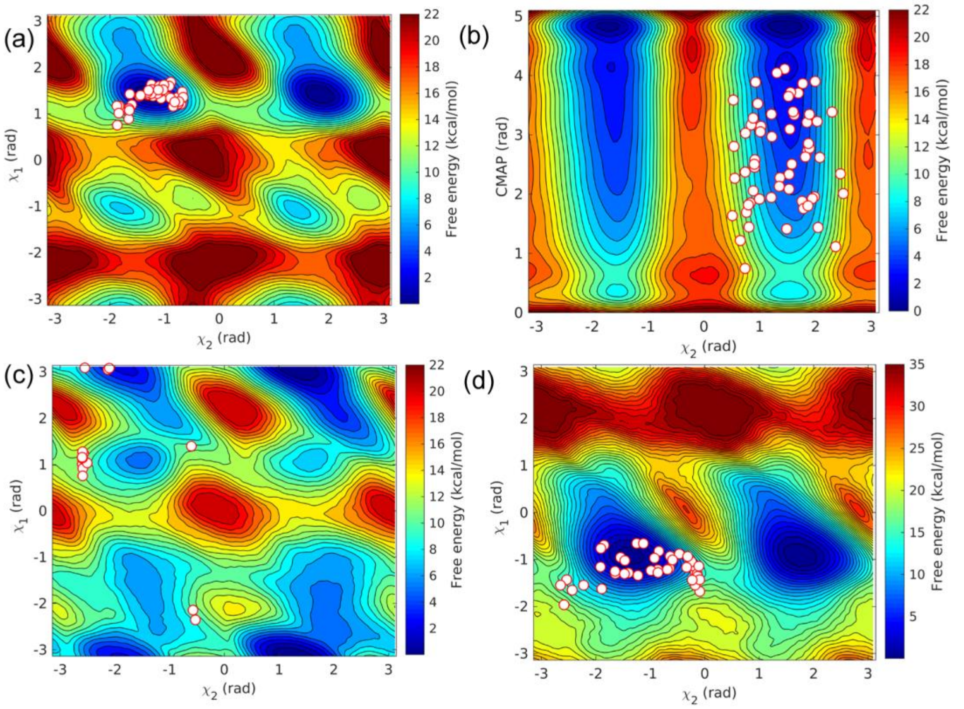

Figure S12. The distribution of trajectory endpoints after flipping for (a) residue F22, (b) residue Y23, (c) residue Y35, and (d) residue F45.

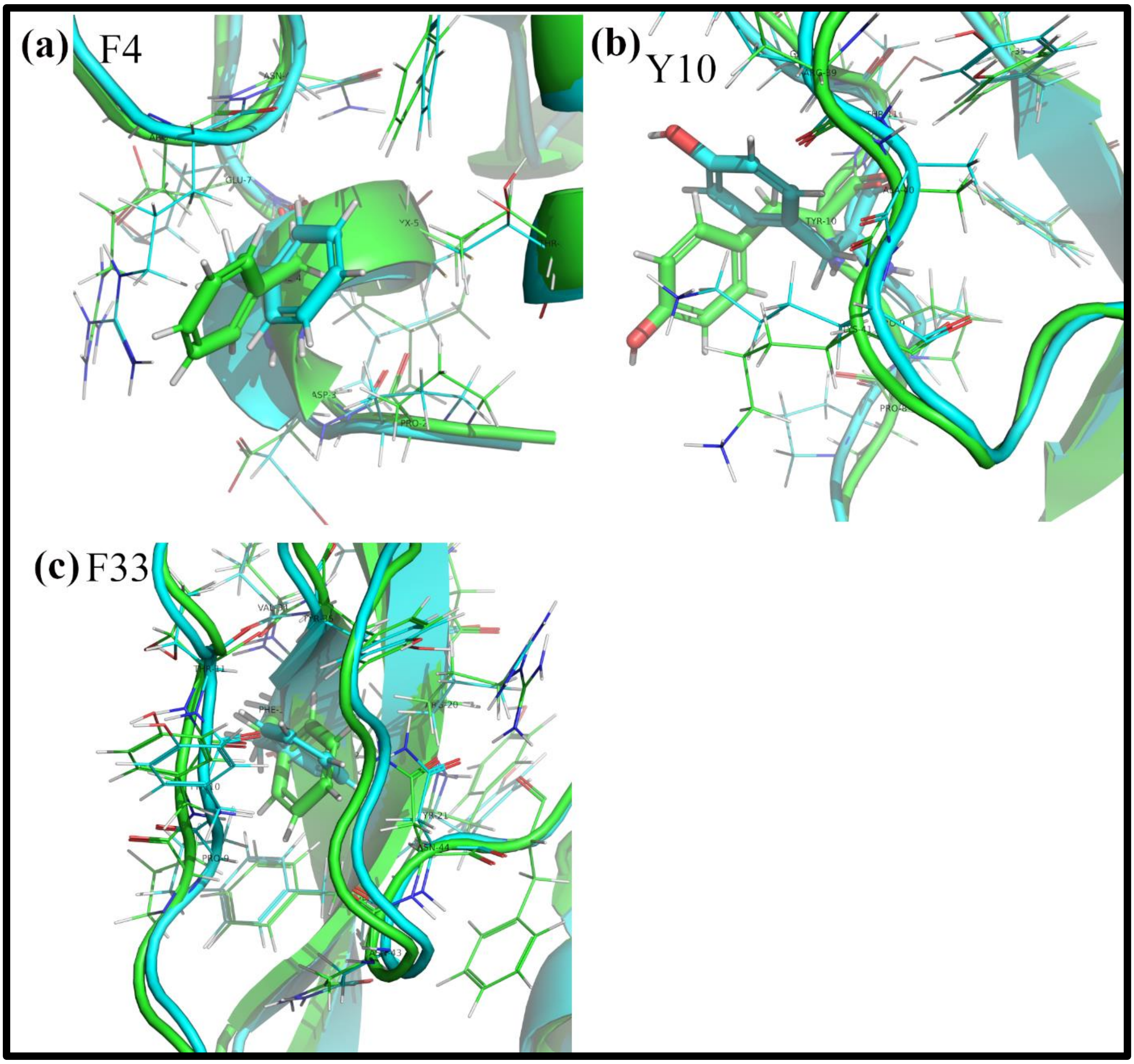

Figure S13. Transition state (TS) structure of (a) residue F4, (b) residue Y10, (c) residue F33 (green: TS structure; cyan: PDB 5PTI).

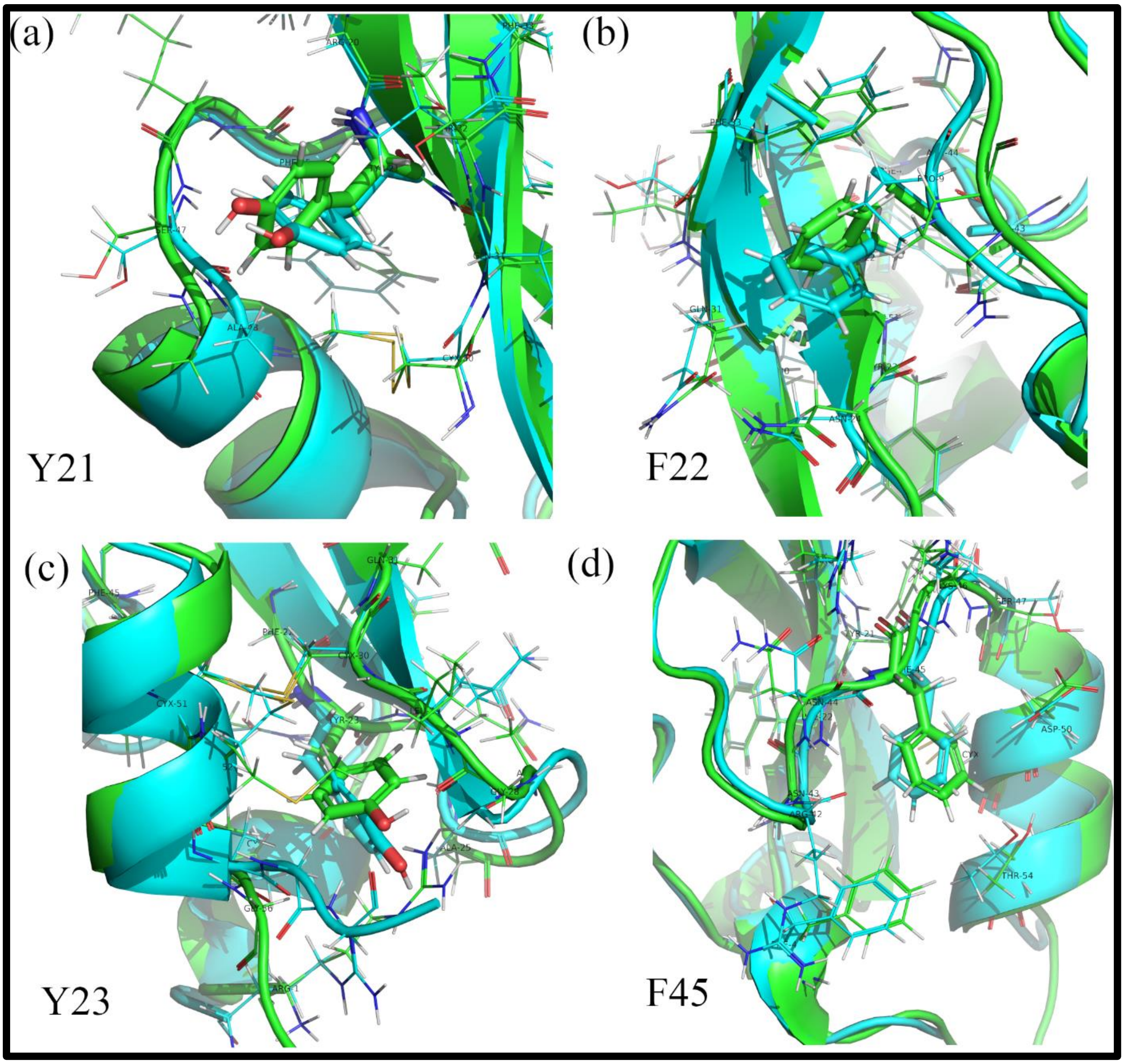

Figure S14. Transition state (TS) structure of (a) residue Y21, (b) residue F22, (c) residue Y23, and (d) residue F45 (green: TS structure; cyan: PDB 5PTI).

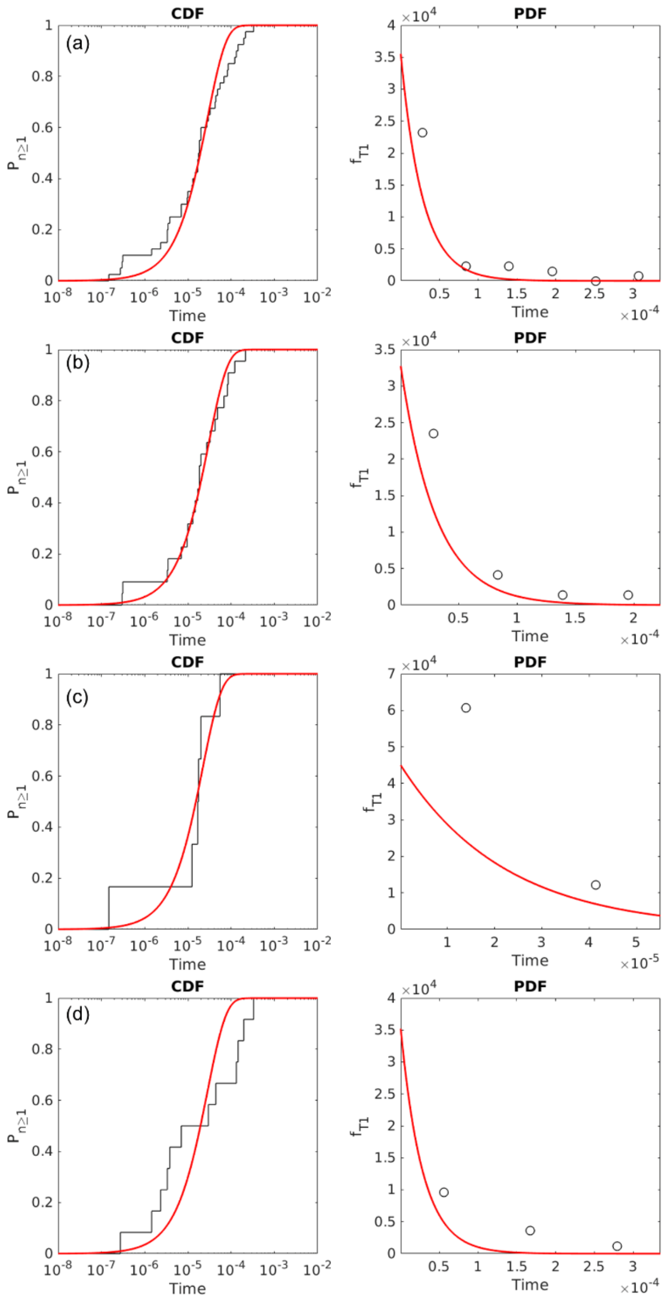

Figure S15. Theoretical Poisson fit to cumulative distribution of transition timescales obtained from $\chi_2 - \chi_1$ infrequent metadynamics simulations of residue F4 by considering (a) all 40 simulations (p-value = 0.32 and μ ln 2/tm = 1.79), (b) 22 $\chi_2 - \chi_1$ assisted A-I1-I1’ transition (p-value = 0.82 and μ ln 2/tm = 1.48) , (c) 6 A-I2’ transitions (p-value = 0.71 and μ ln 2/tm = 0.80) , (d) 12 A-A’ direct transitions (p-value = 0.13 and μ ln 2/tm = 2.78).

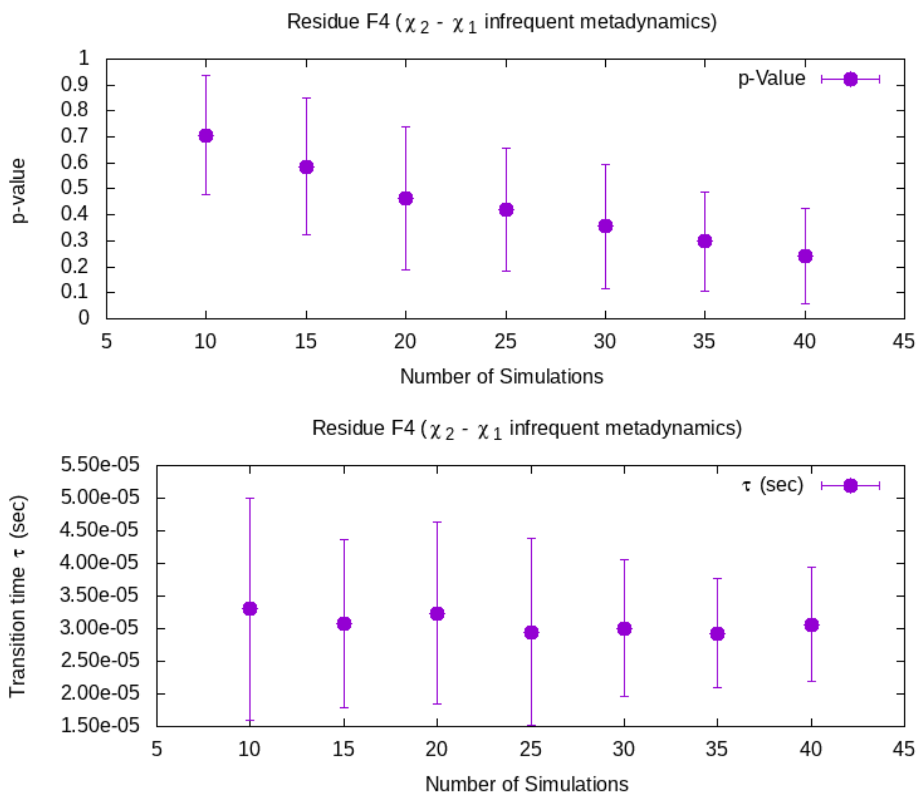

Figure S16. Errors in p-value and transition timescales from $\chi_2 - \chi_1$ infrequent metadynamics simulations of residue F4 obtained with bootstrapping.

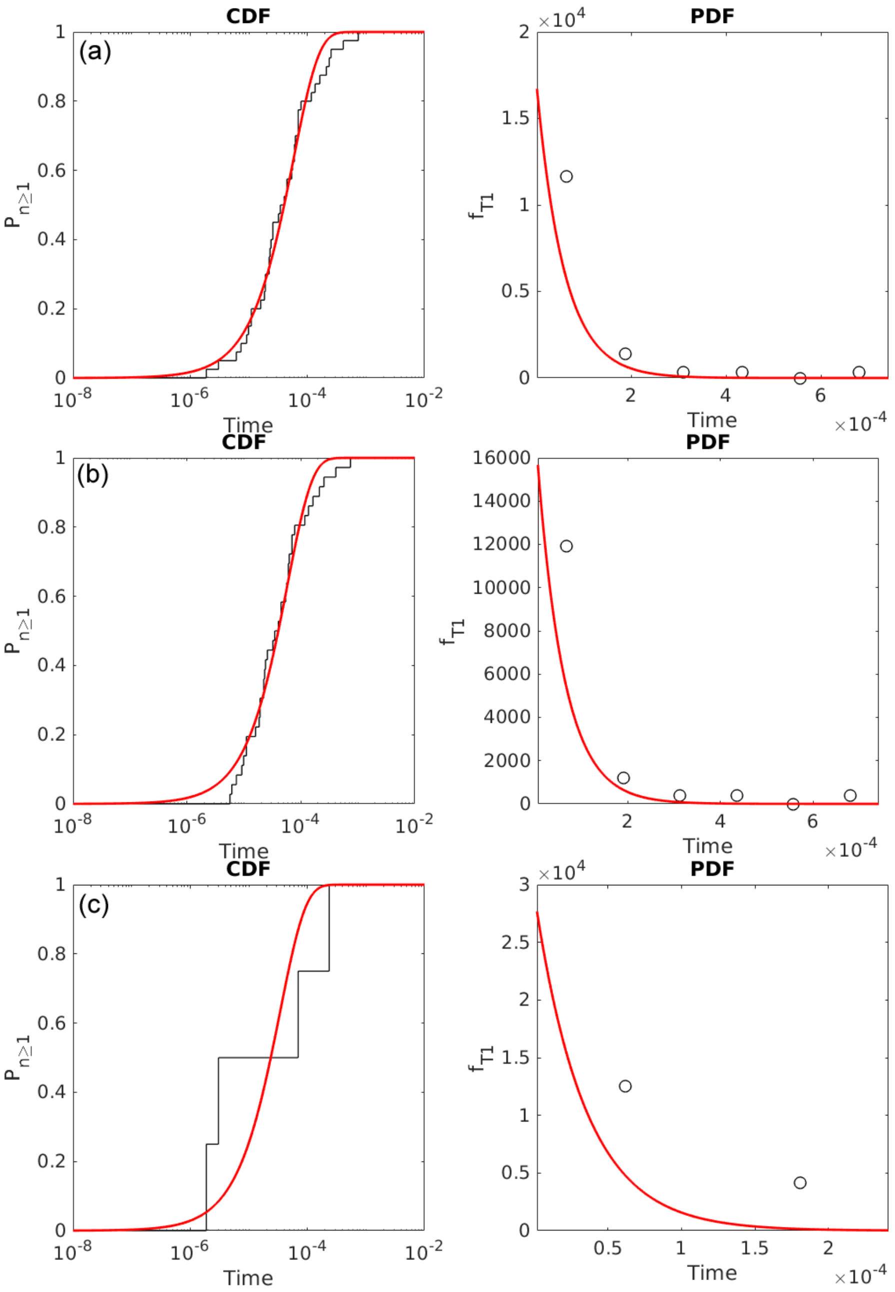

Figure S17. Theoretical Poisson fit to cumulative distribution of transition timescales obtained from χ2- χ1 infrequent metadynamics simulations of residue Y10 by considering (a) all 40 simulations (p-value = 0.80 and μ ln 2/tm = 1.54), (b) 36 $\chi_2 - \chi_1$ driven A-I1-I1' transition (p-value = 0.89 and μ ln 2/tm = 1.54), (c) 4 A-A' direct transitions (p-value = 0.39 and μ ln 2/tm = 1.48).

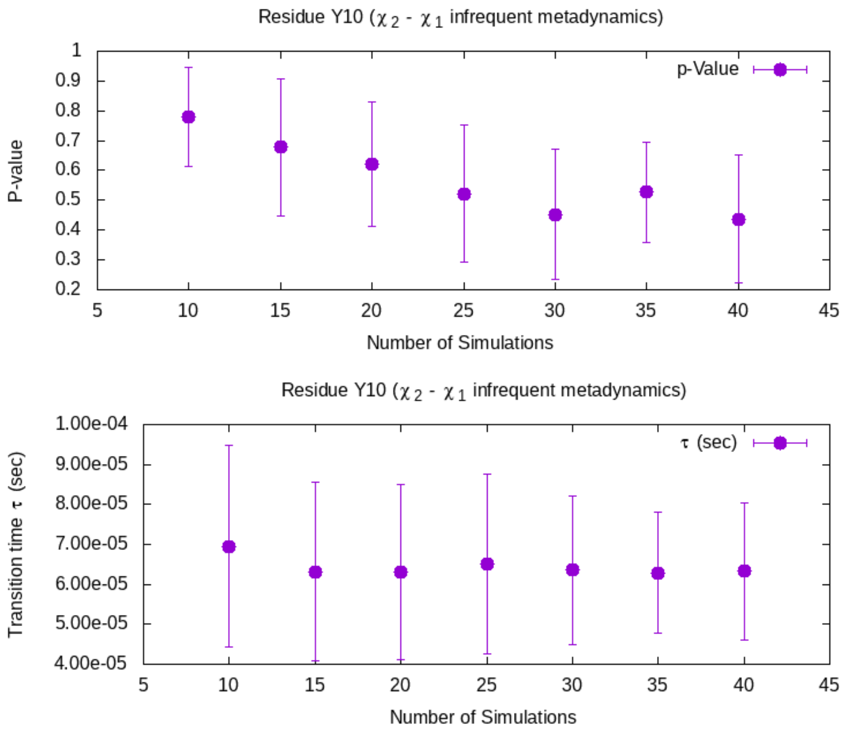

Figure S18. Errors in p-value and transition timescales from $\chi_2 - \chi_1$ infrequent metadynamics simulations of residue Y10 obtained with bootstrapping.

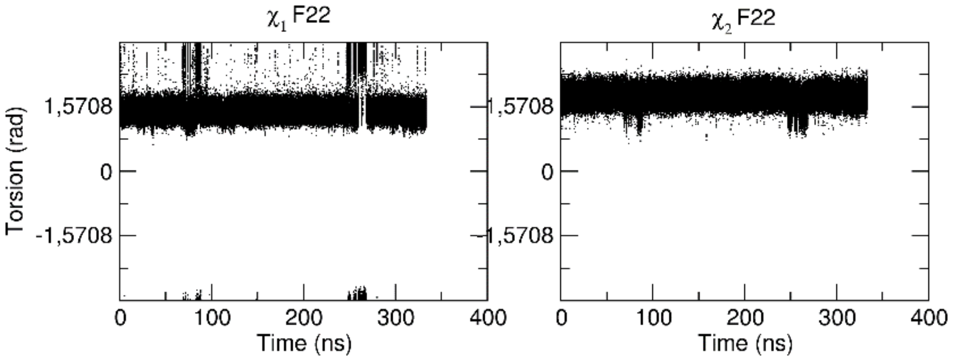

Figure S19. χ_1 and χ_2 torsion values for residue F22 during metadynamics simulation of residue F33.

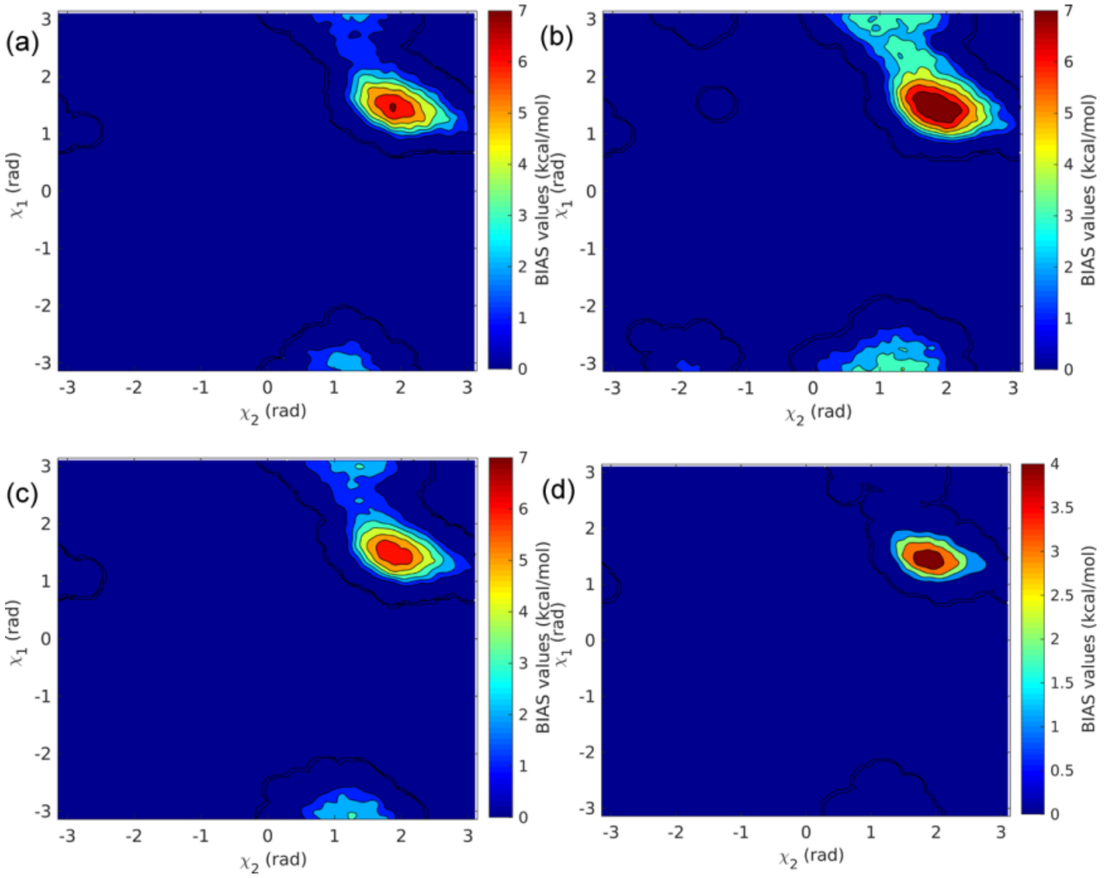

Figure S20. The illustration of bias deposition in the barrier region along χ_1 during infrequent metadynamics simulation along $\chi_2 - \chi_1$ of F33 in some of the trajectories in panels (a)-(c). Panel (d) shows an example where bias is not deposited in the TS region.

Table S3. Results of infrequent metadynamics simulations of residue F33 with wall bias and without wall bias.

Residue F33	Number of simulations	t_{flip} (s)	k_{flip} (s^{-1})	p-value	μ ln 2/tm
No wall bias	40	3.50×10^{-5}	2.86×10^{4}	0.03	2.47
With wall bias	12	3.14×10^{-5}	3.18×10^{4}	0.52	3.78

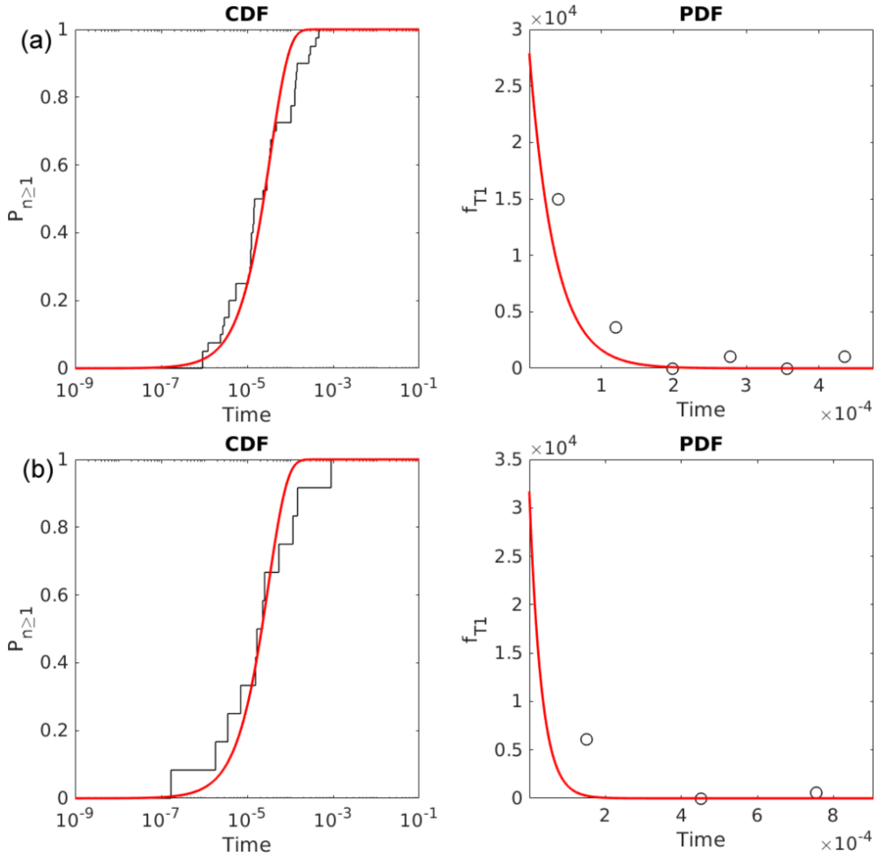

Figure S21. Theoretical Poisson fit to the cumulative distribution of transition timescales obtained from $\chi_2 - \chi_1$ infrequent metadynamics simulations of residue F33 (a) without repulsive walls, and (b) with repulsive wall potential (UPPER_WALL) at $\chi 1 = 2.4$ rad to avoid bias deposition on transition state (12 simulations).

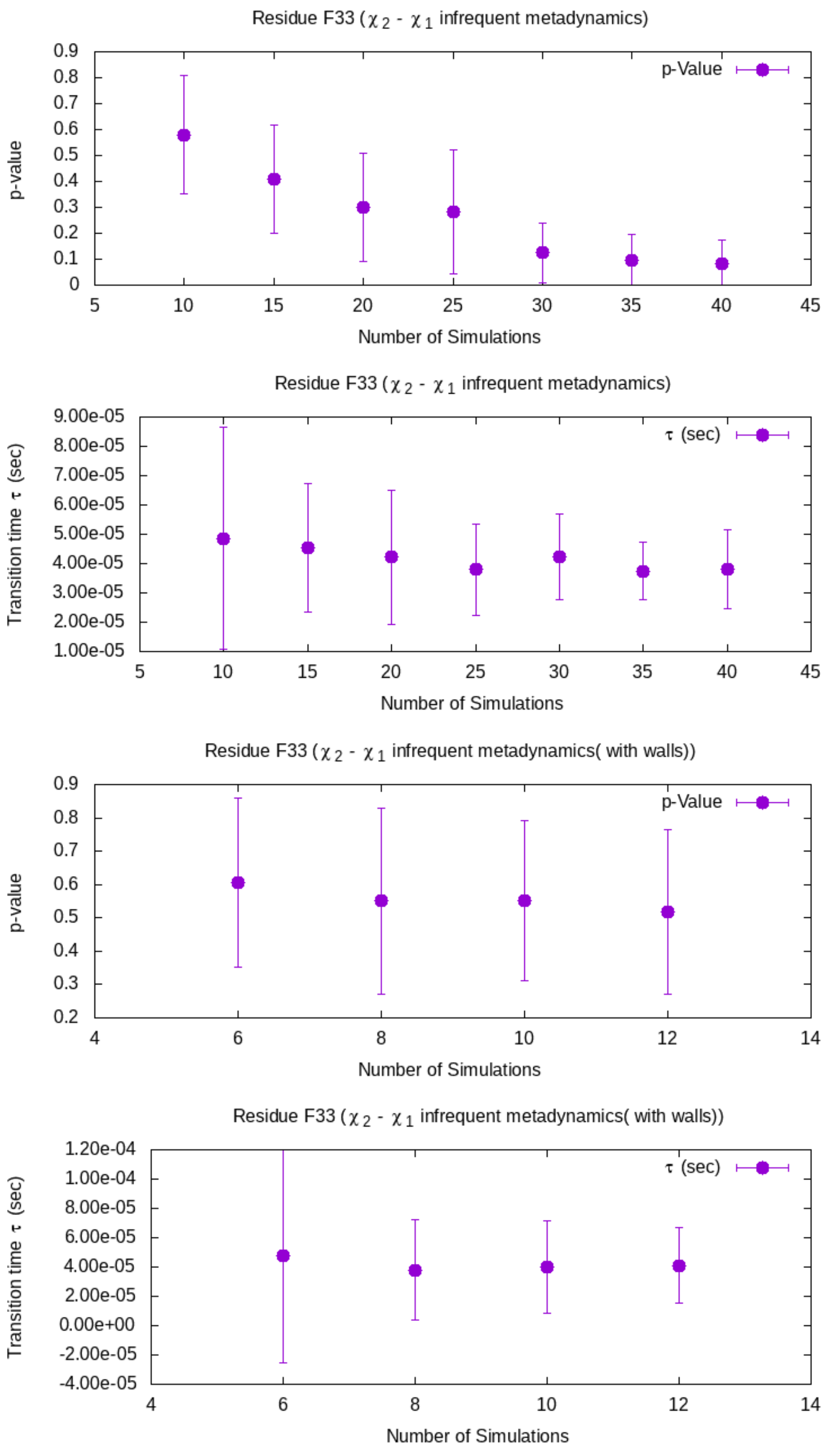

Figure S22. Errors in p-value and transition timescales from $\chi_2 - \chi_1$ infrequent metadynamics simulations of residue F33 without walls (upper two panels) and with wall bias (lower two panels) obtained with bootstrapping.

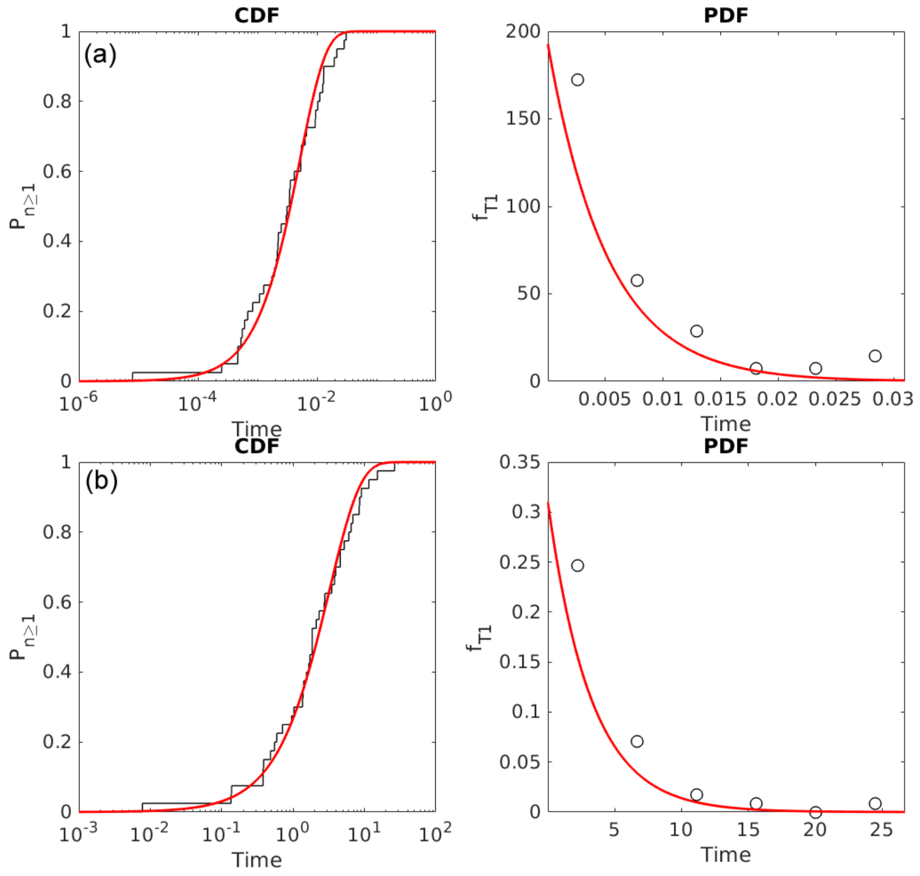

Figure S23. Theoretical Poisson fit to the cumulative distribution of transition timescales obtained from $\chi_2 - \chi_1$ infrequent metadynamics simulations of (a) residue Y21 and (b) F45.

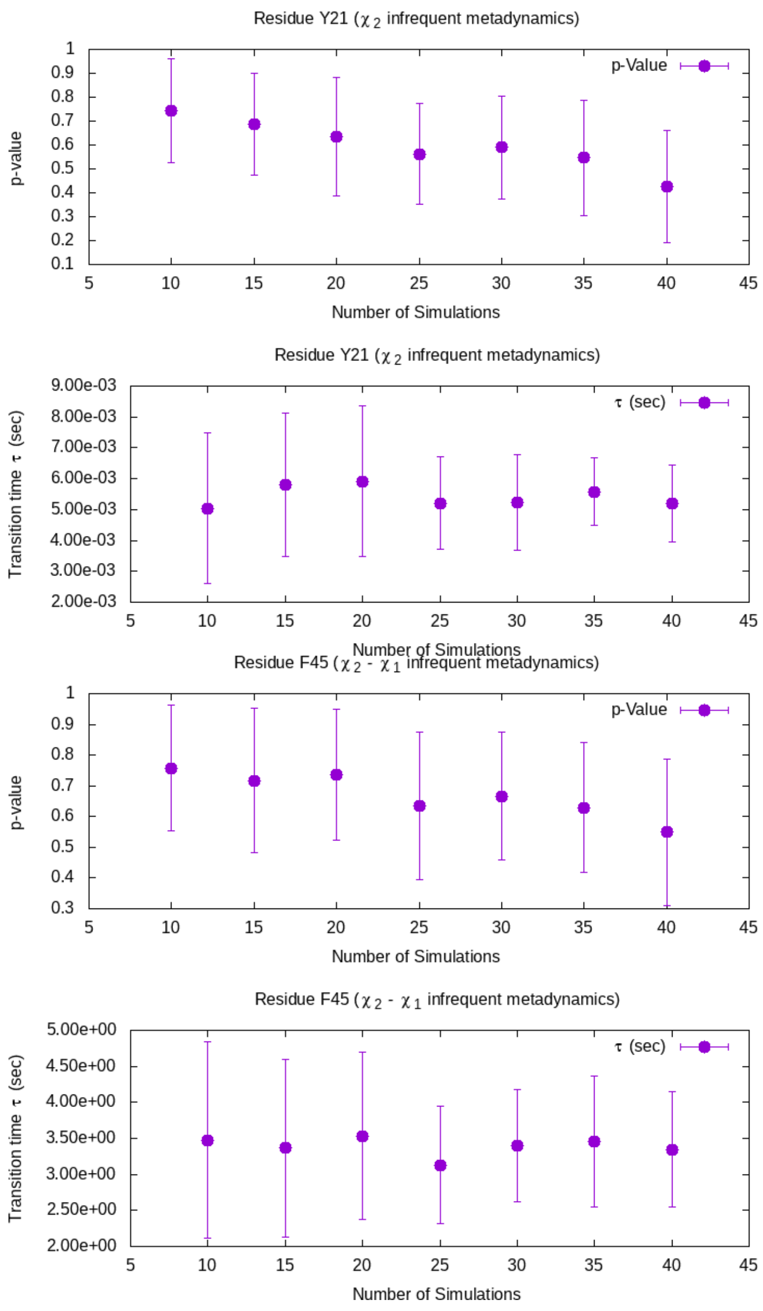

Figure S24. Errors in p-value and transition timescales from χ_2 infrequent metadynamics simulations of residue Y21 (upper two panels) and $\chi_2 - \chi_1$ biased residue F45 (lower two panels) obtained with bootstrapping.

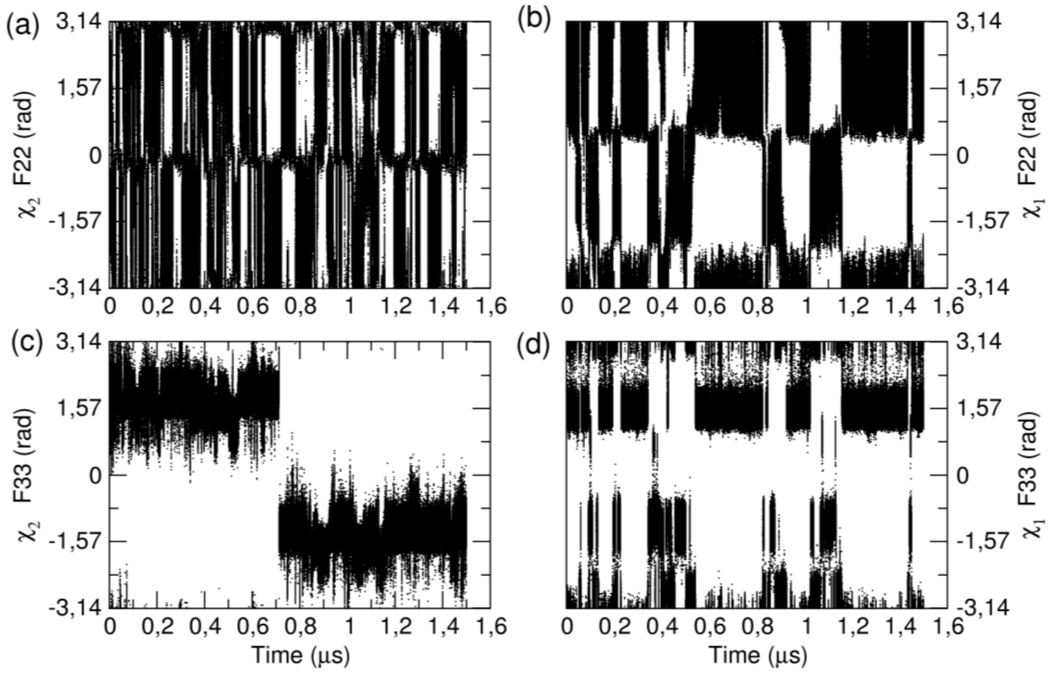

Figure S25. χ_1 and χ_2 torsion values for residue F22 and F33 in the metadynamics simulation of residue F22.

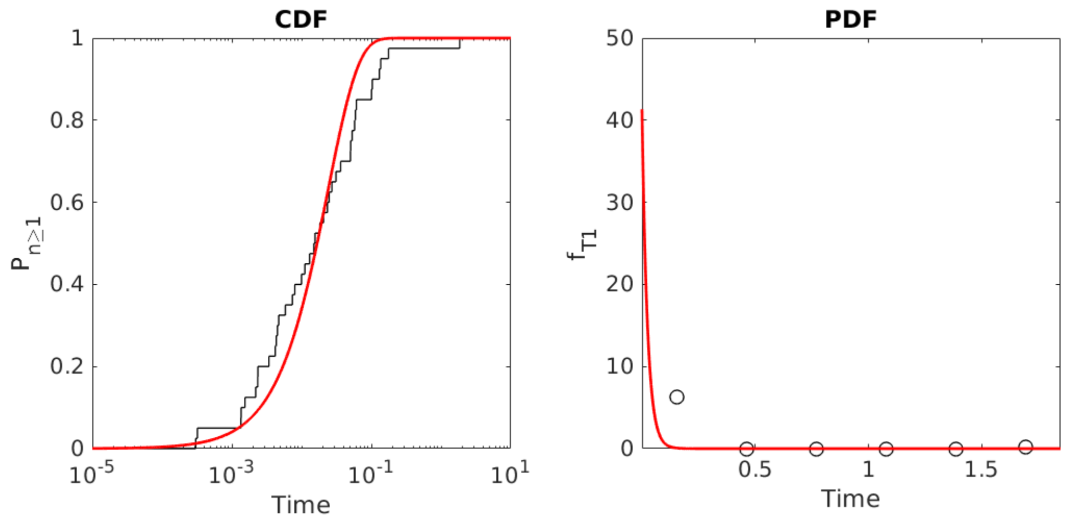

Figure S26. Theoretical Poisson fit to the cumulative distribution of transition timescales obtained from $\chi_2 - \chi_1$ infrequent metadynamics simulations of residue F22.

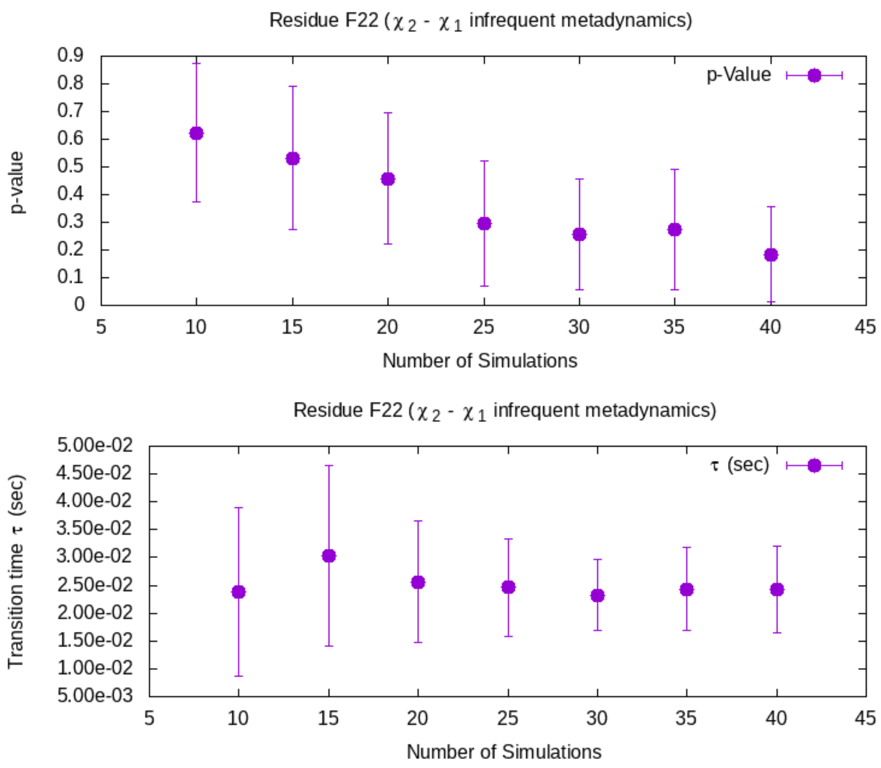

Figure S27. Errors in p-value and transition timescales from $\chi_2 - \chi_1$ infrequent metadynamics simulations of residue F22.

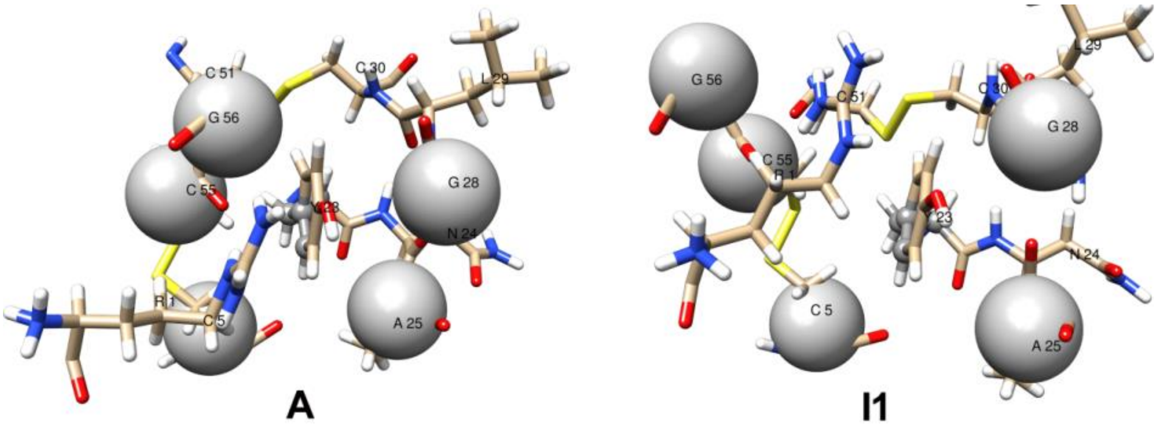

Figure S28. Representative structures observed in each minimum of χ_2-CMAP free energy surface of residue Y23.

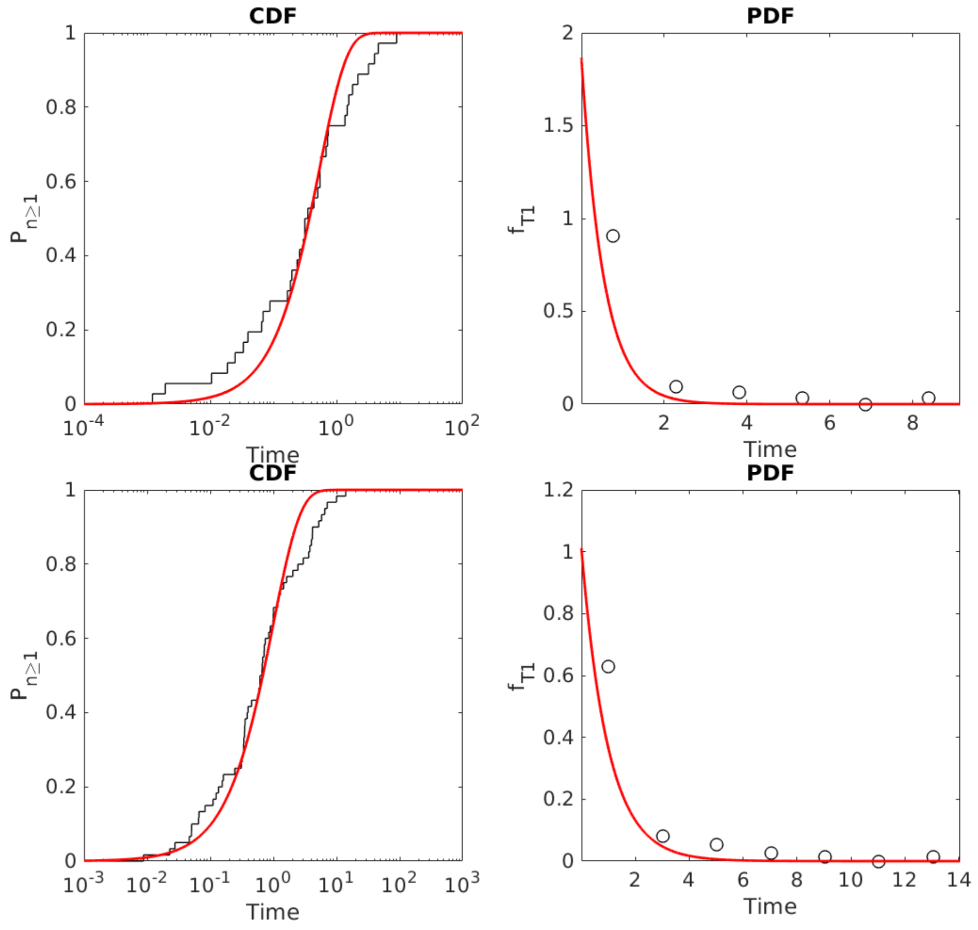

Figure S29. Theoretical Poisson fit to the cumulative distribution of τ_i (s) obtained from $\chi_2 - \chi_1$ (top panel, 26 simulations, p-value = 0.22 and μ ln 2/tm = 2.17) and χ_2-CMAP (bottom panel, 60 simulations, p-value = 0.08 and μ ln 2/tm = 1.80) InMetaD simulations of residue Y23.

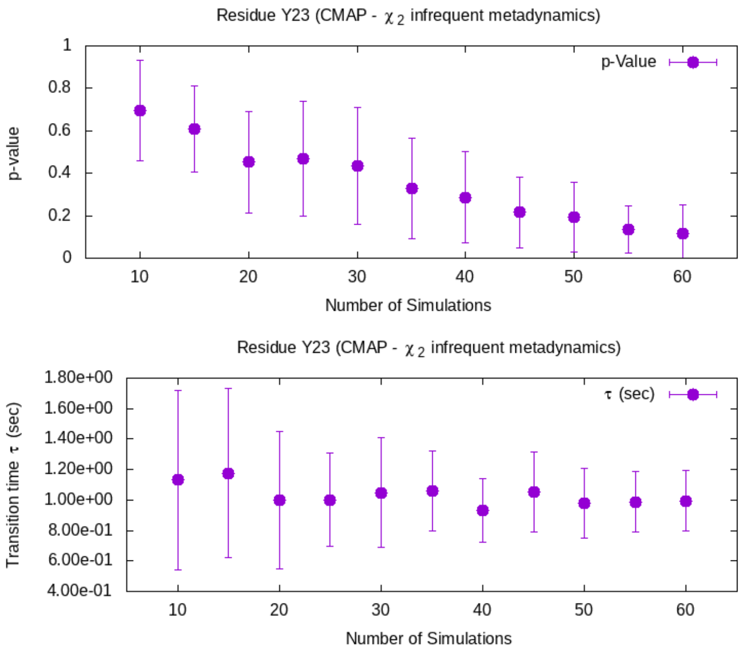

Figure S30. Errors in p-value and transition timescales from χ_2- CMAP biased infrequent metadynamics simulations of residue Y23.

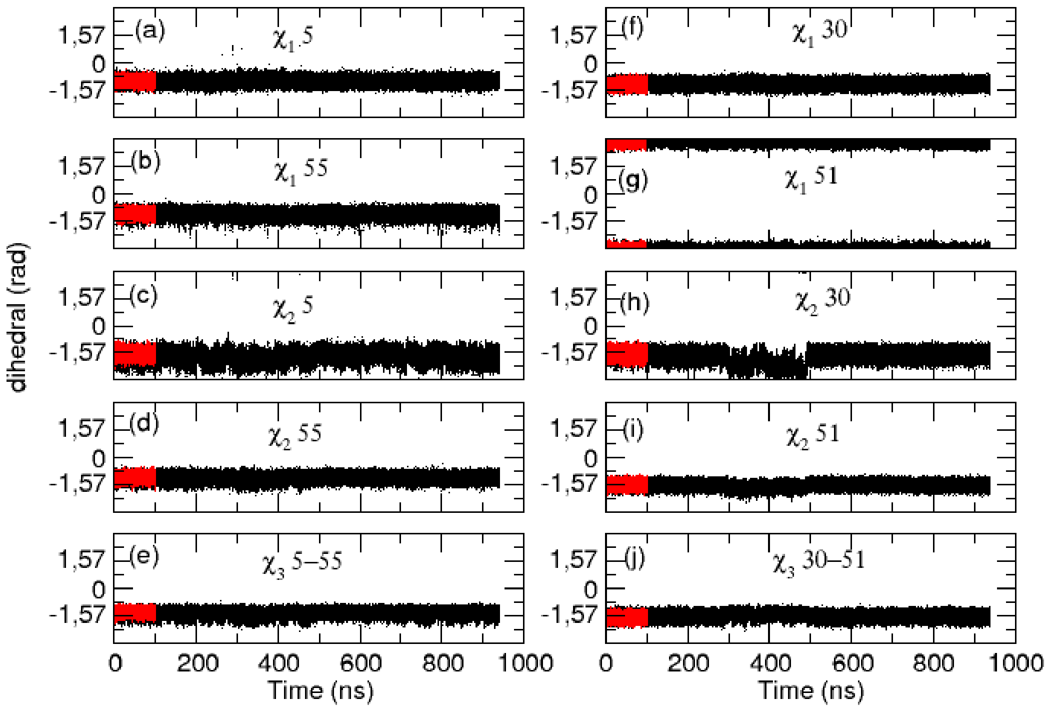

Figure S31. Values of χ_2, χ_1 , and χ_3 torsions of 5-55 and 30-51 disulfide bridges from χ_2-CMAP metadynamics simulation of residue Y23 (black) and 100 ns unbiased simulation (red).

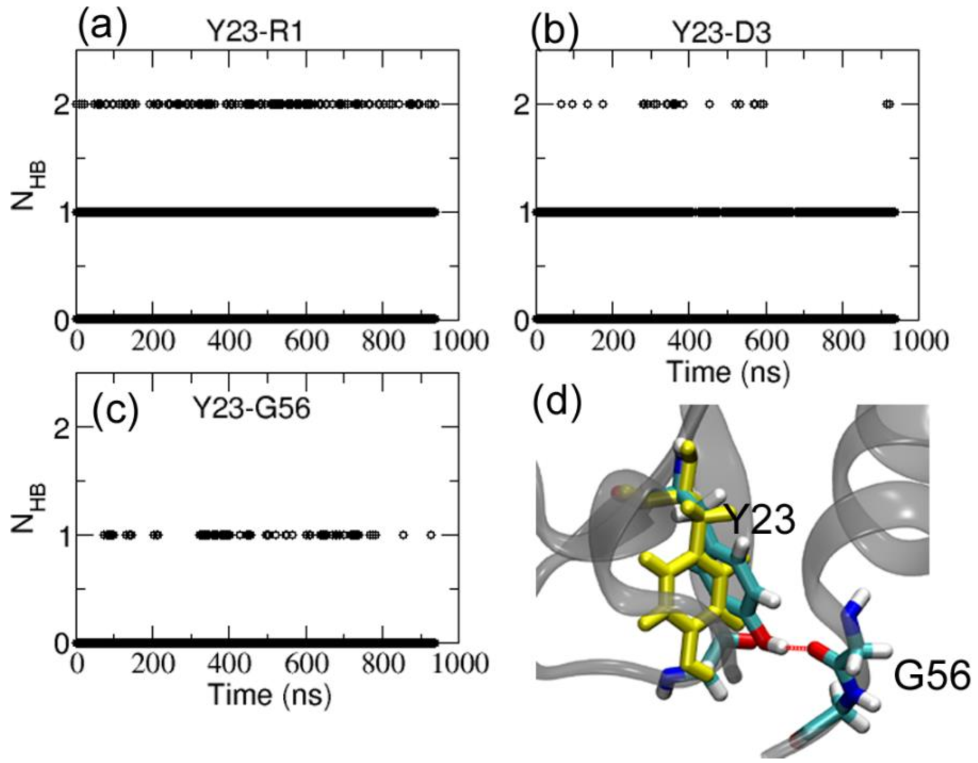

Figure S32. The number of hydrogen bonds between residues (a) Y23-R1, (b) Y23-D3, (c) Y23-G56, and (d) representative structure showing the hydrogen bonding of Y23 with G56 during the ring flip. The native orientation of the Y23 aromatic side-chain before flipping is shown in yellow color.

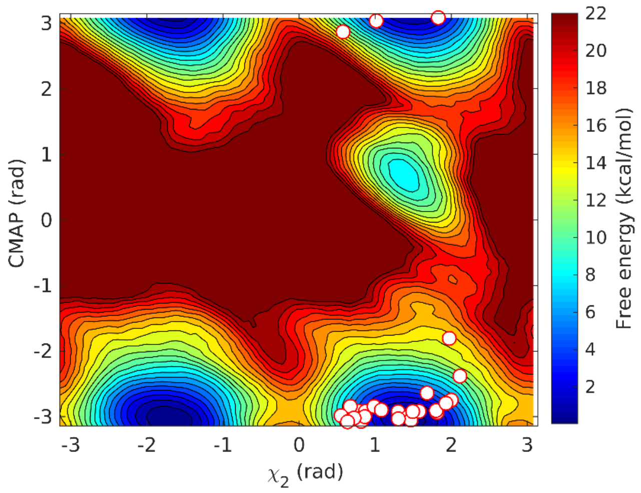

Figure S33. Free energy landscape of $\chi_2 - \chi_1$ biased metadynamics simulation of residue Y23. White circles indicate the 26 trajectory endpoints immediate after the ring flip.

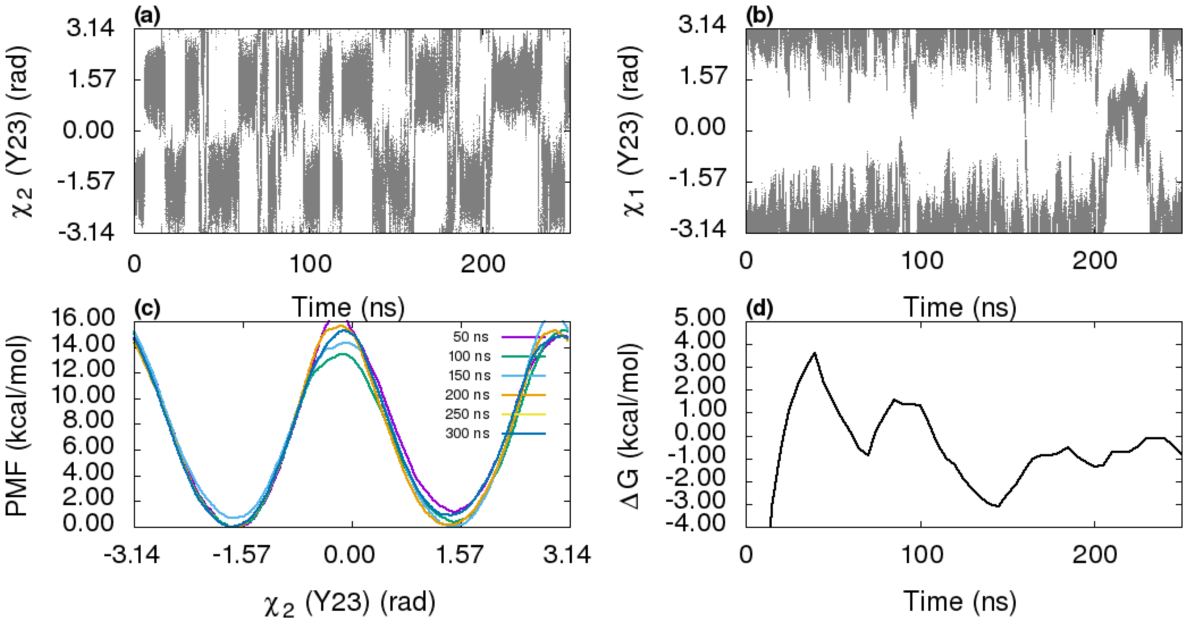

Figure S34. The time series of χ_2 and χ_1 during $\chi_2 - \chi_1$ metadynamics simulation of residue Y23 in figures (a) and (b), respectively. (c) the projection of free energy on χ_2 in time intervals of 50 ns. (d) The free energy difference between two symmetric states along χ_2.

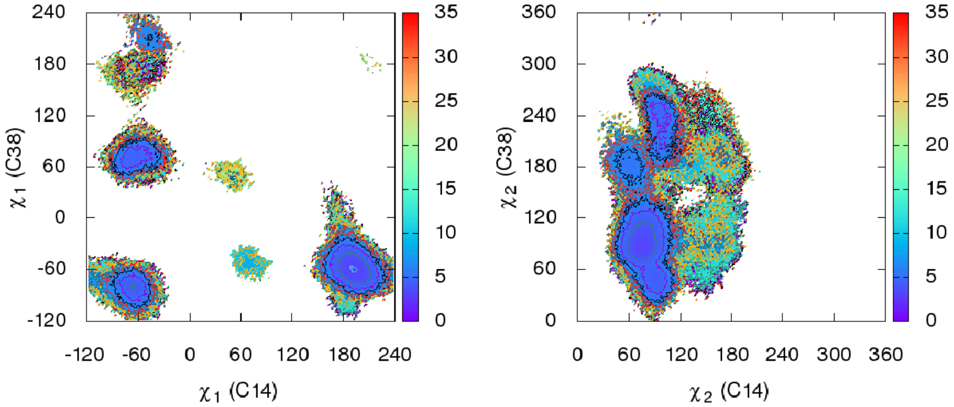

Figure S35. The distribution of side-chain torsions of residues C14 and C38 (disulfide bridge C14-C38) in case of non-converged $\chi_2 - \chi_1$ metadynamics simulation of residue Y35. The free energy in kcal/mol.

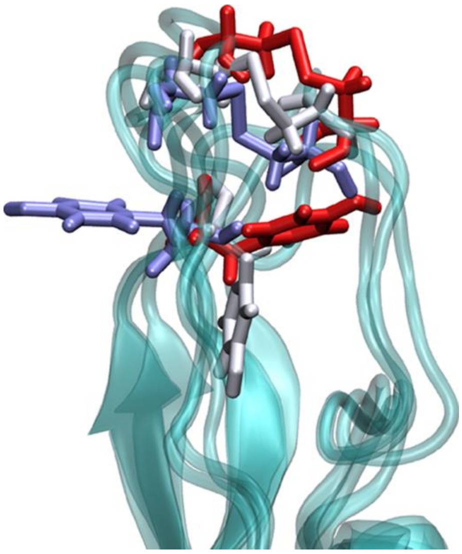

Figure S36. The conformations of residue Y35 and C14-C38 disulfide bridge in the native state (red) and unusual conformations (light and dark blue) during well-tempered metadynamics simulations with χ_2, χ_1 of Y35, and χ_1 dihedral of a C14-C38 disulfide bridge.

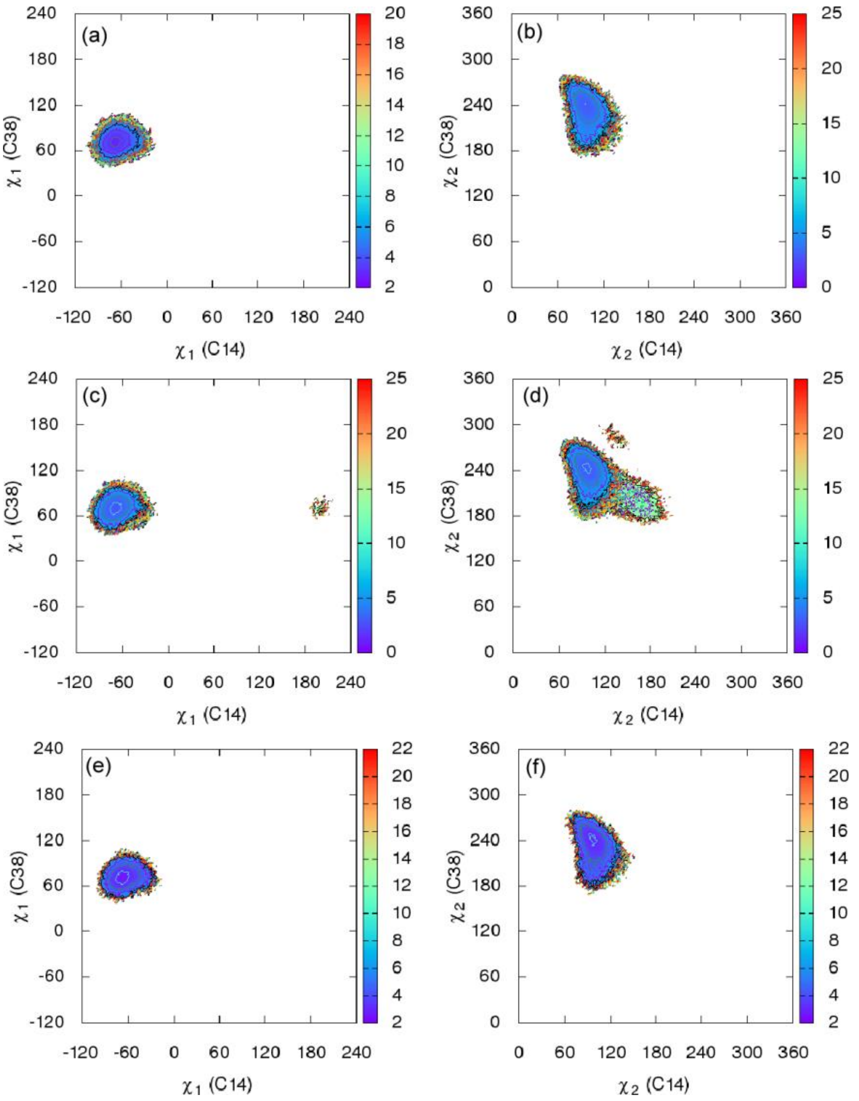

Figure S37. The distribution of side-chain torsions of residues C14 and C38 (disulfide bridge C14-C38) in case of each converged metadynamics simulations of fast flipping residues: (a) and (b) residue F4; (c) and (d) Residue Y10; (e) and (f) residue F33. The free energy in kcal/mol.

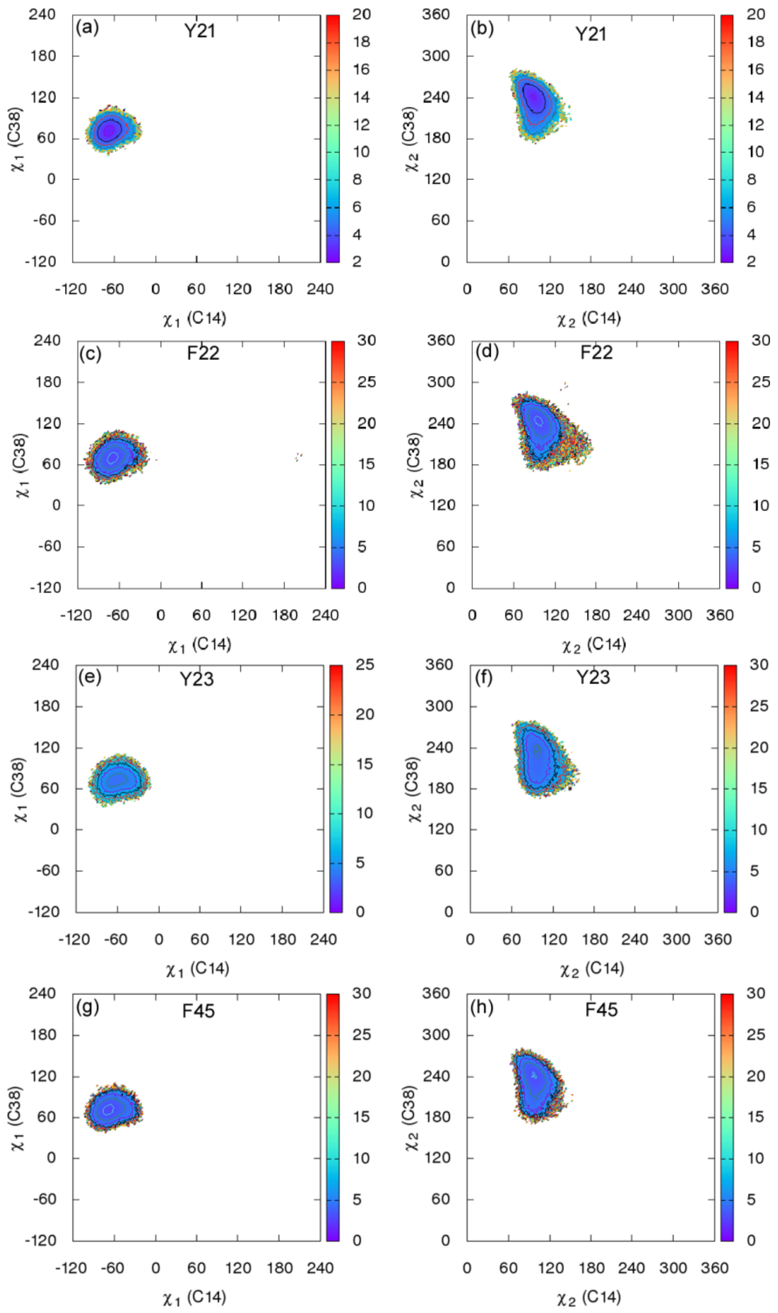

Figure S38. The distribution of side-chain torsions of residues C14 and C38 (disulfide bridge C14-C38) in case of each converged metadynamics simulations for residue Y21 (figures (a) and (b); residue F22 (figures (c) and (d)); residue Y23 (figures (e) and (f)); and residue F45 (figures (g) and (h)). The free energy in kcal/mol.

Solvent Accessible Surface Area (SASA)

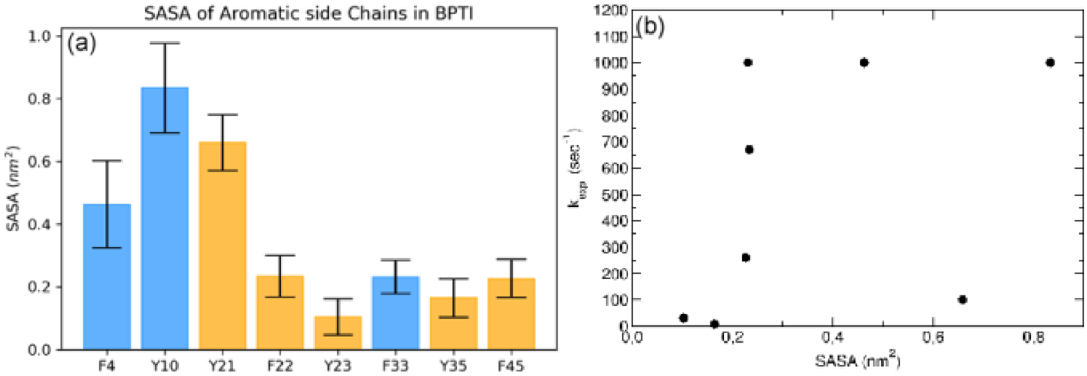

Figure S39. (a) SASA for aromatic side chains from standard 100 ns MD simulation. The slow (orange) and fast (blue) flipping residues are shown in a different color. (b) Correlation between flipping rate (k_{flip}) and SASA (R=0.39). The SASA of all eight residues using the *gmx sasa* command in *GROMACS* with a standard solvent probe radius of 1.4 Å.

Circular Correlation of Side-chain torsions

The circular correlation coefficient[1] for dihedrals is calculated using *astropy* python package[2].

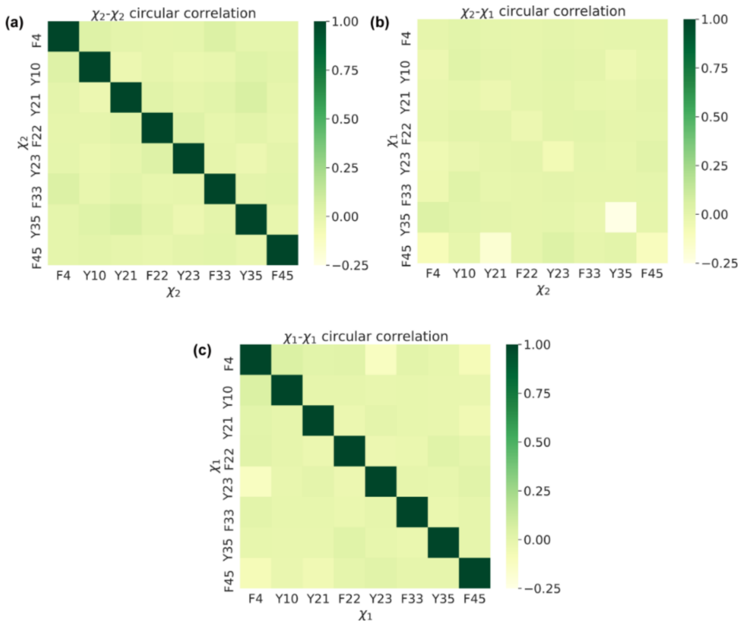

Figure S40. The circular correlation coefficient values of torsions χ_2 and χ_1 of aromatic residues of BPTI obtained from 1-ms-long trajectory.

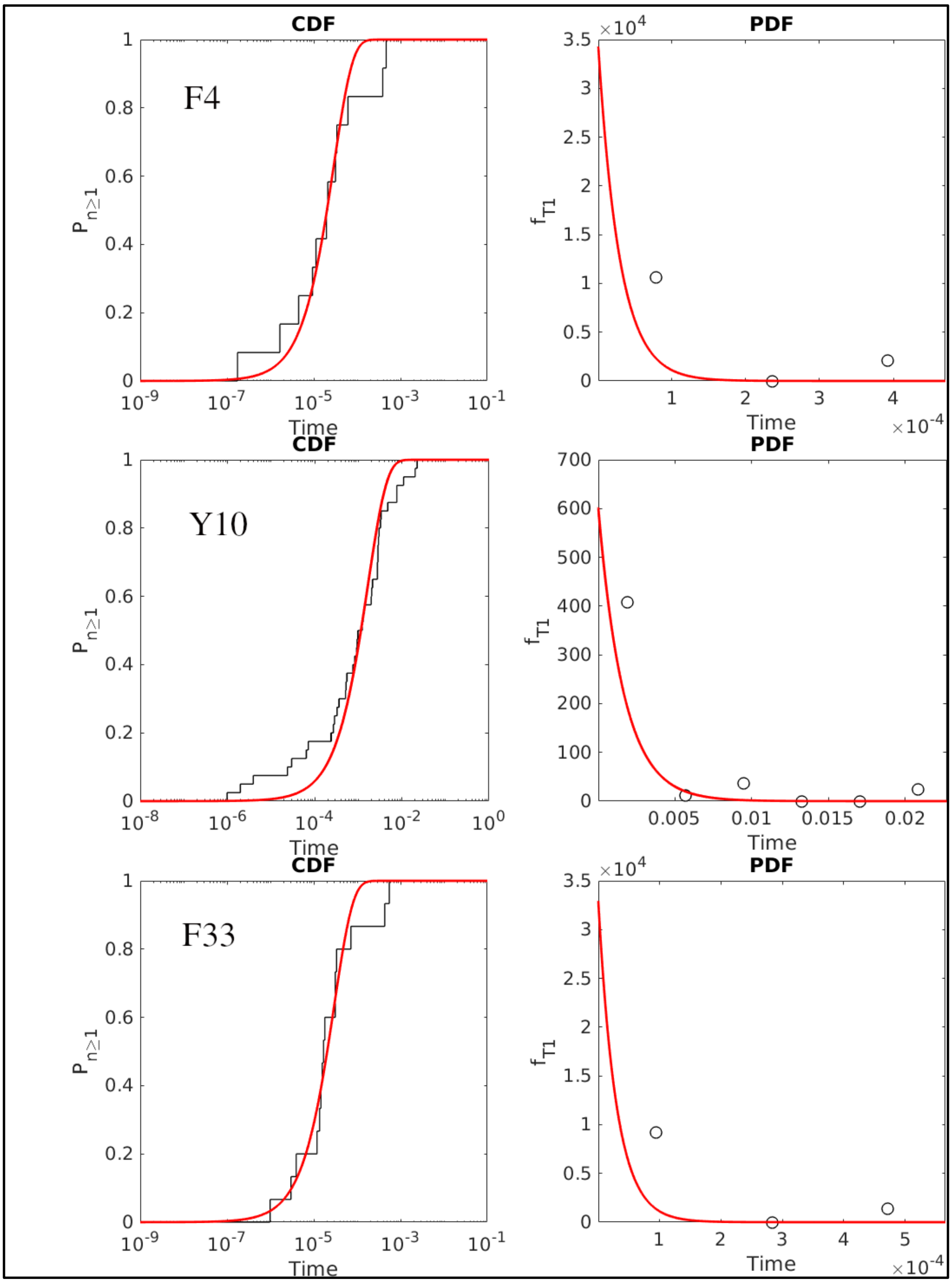

Figure S41. Theoretical Poisson fit to the cumulative distribution of transition timescales obtained **from χ_2 infrequent metadynamics** simulations of residue F4, Y10 and F33.

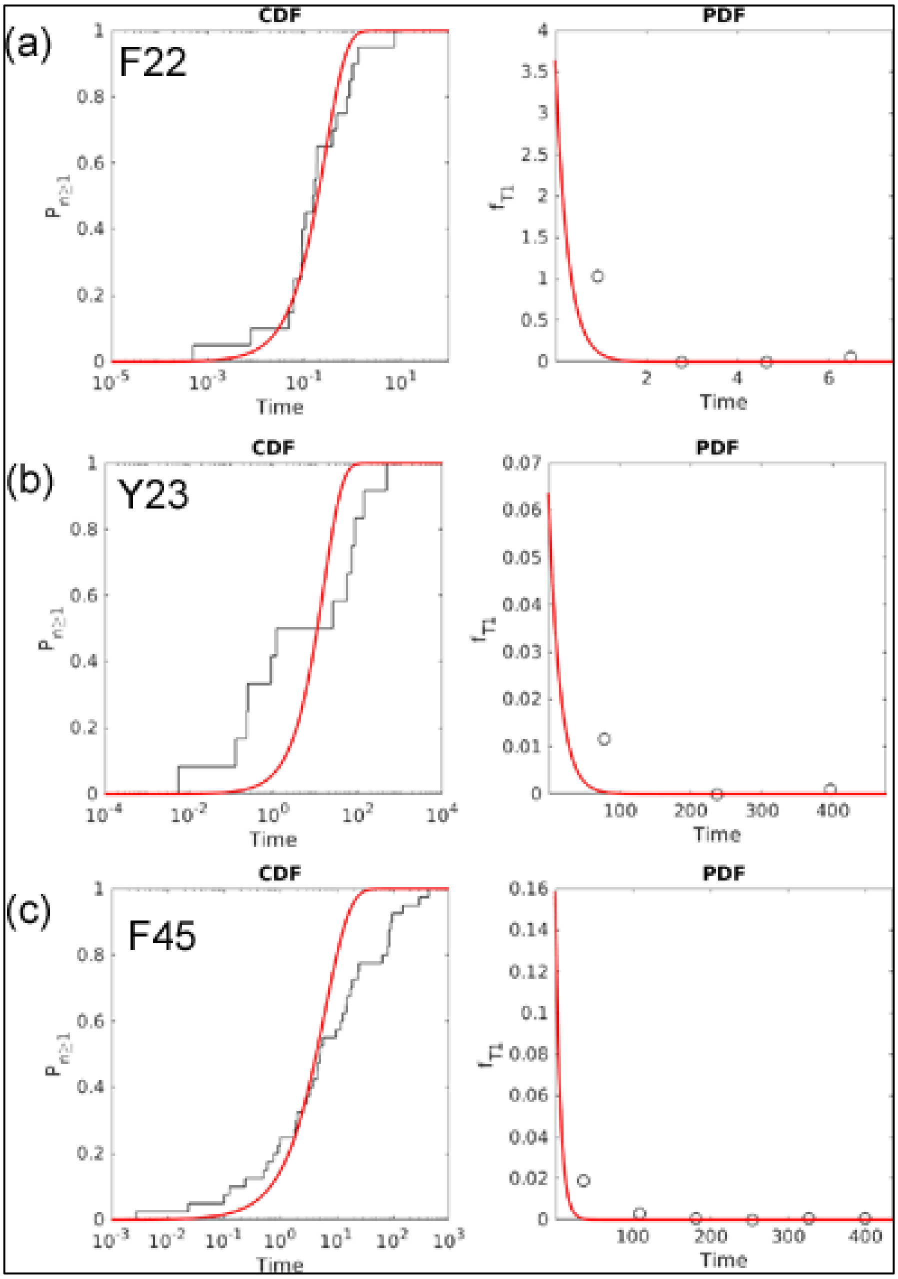

Figure S42. Theoretical Poisson fit to the cumulative distribution of transition timescales obtained **from χ_2 infrequent metadynamics** simulations of residue F22, Y23, and F45.

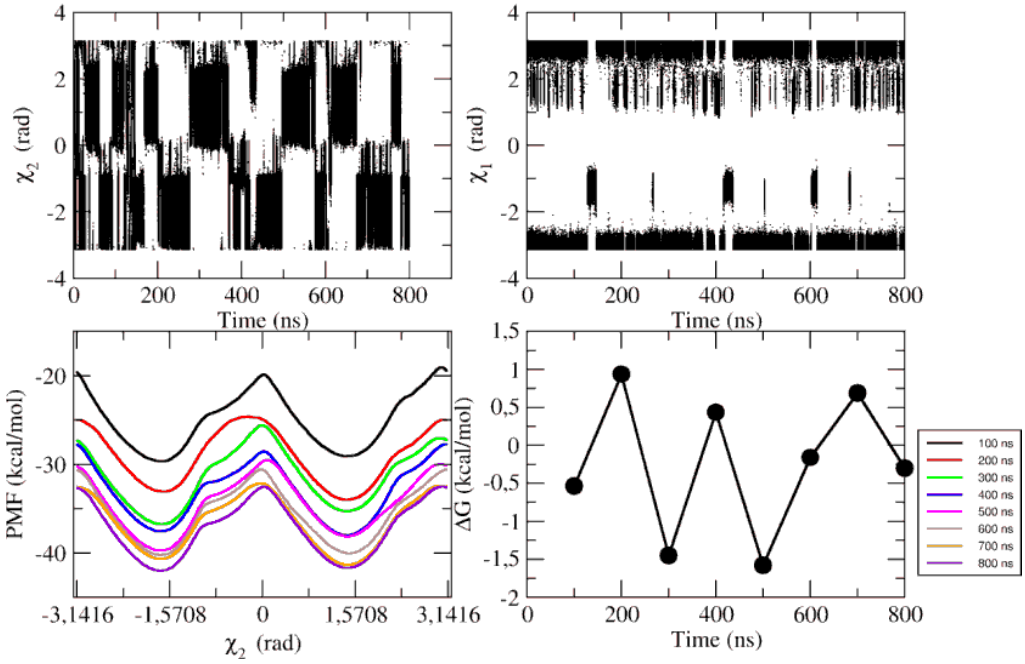

Figure S43. Results of χ_2 metadynamics of residue Y10: (a) χ_2 vs. simulation time, (b) χ_1 vs. simulation time, and (c) test of convergence along χ_2 and (d) free energy difference between two identical states. The free energy difference between these two states should be ideally zero as they are identical states.

CHARMM simulations result:

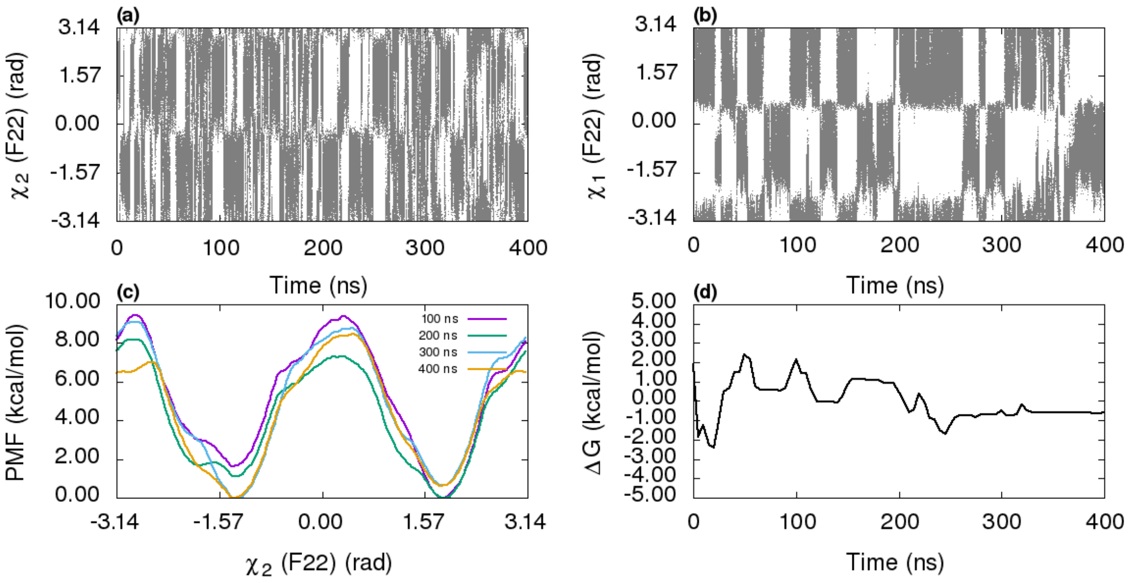

Figure S44. Diffusion of χ_2 and χ_1 during $\chi_2 - \chi_1$metadynamics simulation of residue F22 (with **CHARMM36/standard TIP3P water model**) is shown in figures (a) and (b), respectively. (c) Projection of free energy on χ_2 in time intervals of 100 ns. (d) The free energy difference between two symmetric states along χ_2.

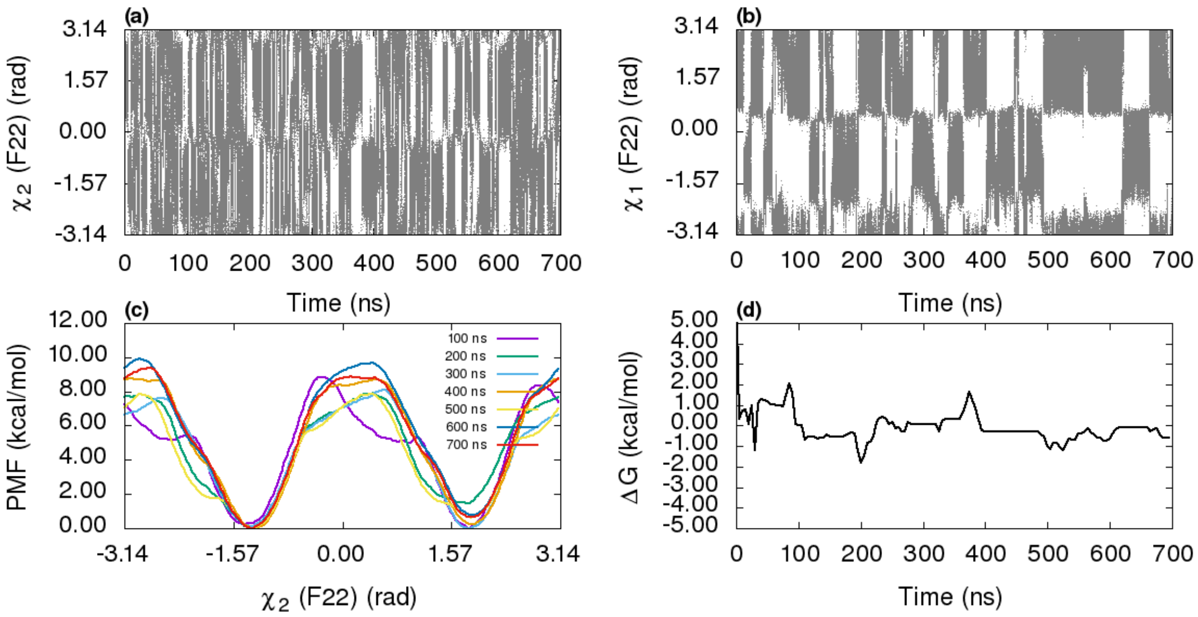

Figure S45. Diffusion of χ_2 and χ_1 during $\chi_2 - \chi_1$metadynamics simulation of residue F22 (with **CHARMM36m/charmm TIP3P water model**) is shown in figures (a) and (b), respectively. (c) Projection of free energy on χ_2 in time intervals of 100 ns. (d) The free energy difference between two symmetric states along χ_2.

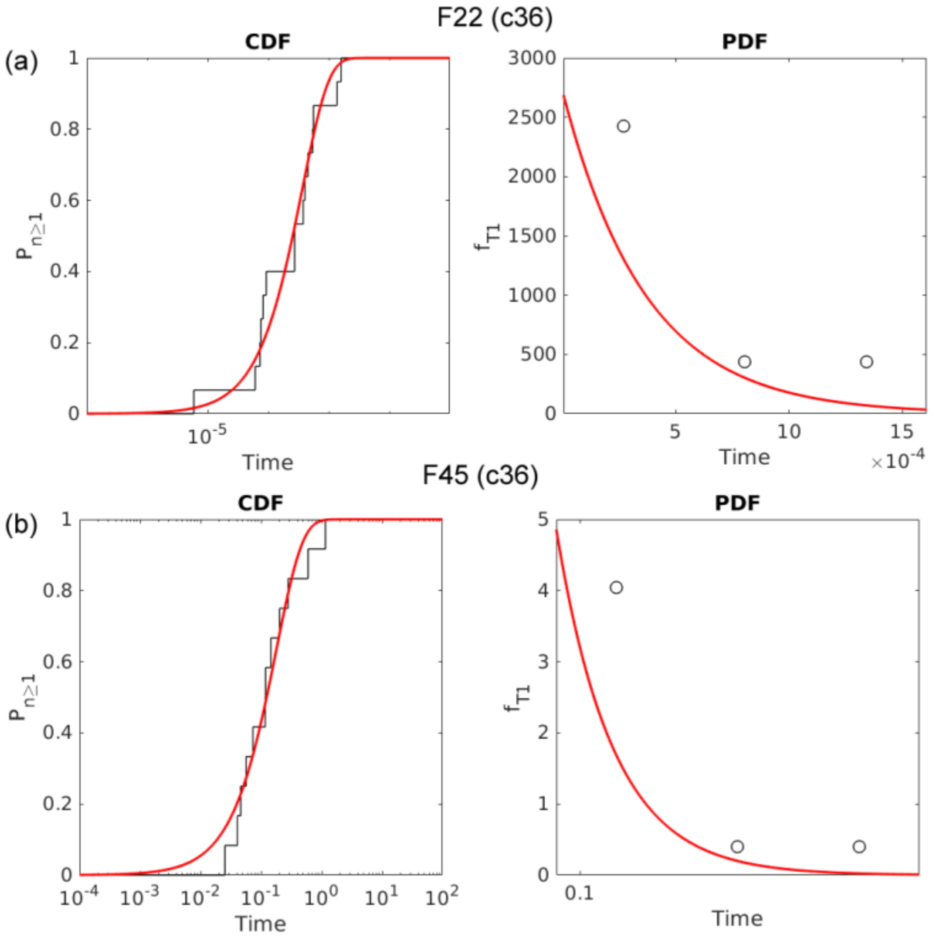

Figure S46. The Poisson fit to cumulative distribution function of transition timescales obtained from infrequent metadynamics simulations of CHARMM36/standard TIP3P force field for (a) residue F22 (p-value = 0.69 and μ ln 2/t_m = 1.24), and (b) residue F45 (p-value = 0.97 and μ ln 2/t_m = 1.40).

Appendix I. Details of VAC derived reaction coordinates.

We observed transient interactions between residues F22, F33, and P9 of BPTI in metadynamics trajectories of $\chi_2 - \chi_1$biased simulations of both F22 and F33 (ff14SB force field). Thus, assuming these interactions as a possible reason for recrossing events in $\chi_2 - \chi_1$infrequently biased simulations, VAC-optimized CV based simulations were performed. We have followed the protocol mentioned in reference [5] (plumID:19.046 at https://www.plumed-nest.org/), and the file for independent component analysis is obtained from the GitHub[6] repository https://github.com/helloyesterday/PLUMED2_TICA.The basis set of following descriptors are used to define a linear combination.

Table S4. List of descriptors.

Residue	Descriptors
F22	$\alpha\beta(\chi_2^{F22})$, $\alpha\beta(\chi_1^{F22})$, $\alpha\beta(\chi_1^{F33})$, d_1
F33	$\alpha\beta(\chi_2^{F33})$, $\alpha\beta(\chi_1^{F33})$, $\alpha\beta(\chi_2^{F22})$, d_2

$\alpha\beta(\theta)$ is a function transformation of periodic dihedral θ (in radians) defined as

$$\alpha\beta(\theta) = \frac{1}{2}[1 + \cos(\theta - \theta^{ref})]$$

θ^{ref}= 1.2 rad was used following work in Ref. 7. χ_2 and χ_1 side-chain torsions of residues F22 and F33 are denoted with superscript residue names ($\boldsymbol{\chi_2^{F22}, \chi_1^{F22}, \chi_2^{F33}, \chi_1^{F33}}$). d_1 and d_2 are distances between atoms of residues P9 and F33.

d_1 = distance between CG atom of P9 and CB atom of F33.

d_2 = distance between C atom of P9 and CB atom of F33.

We used the first 200 ns (F22) and 70 ns (F33) of χ_2 - χ_1 metadynamics simulations based on quasi-equilibrium region, realized by c(t) value, as shown in **figure S10**. The decay of eigenvalues ($\lambda_i, i = 1,2,3,4$ due to 4 CVs) is shown figure below and based on the spectral gap ($\lambda_i - \lambda_{i+1}$) between consecutive eigenvalues.

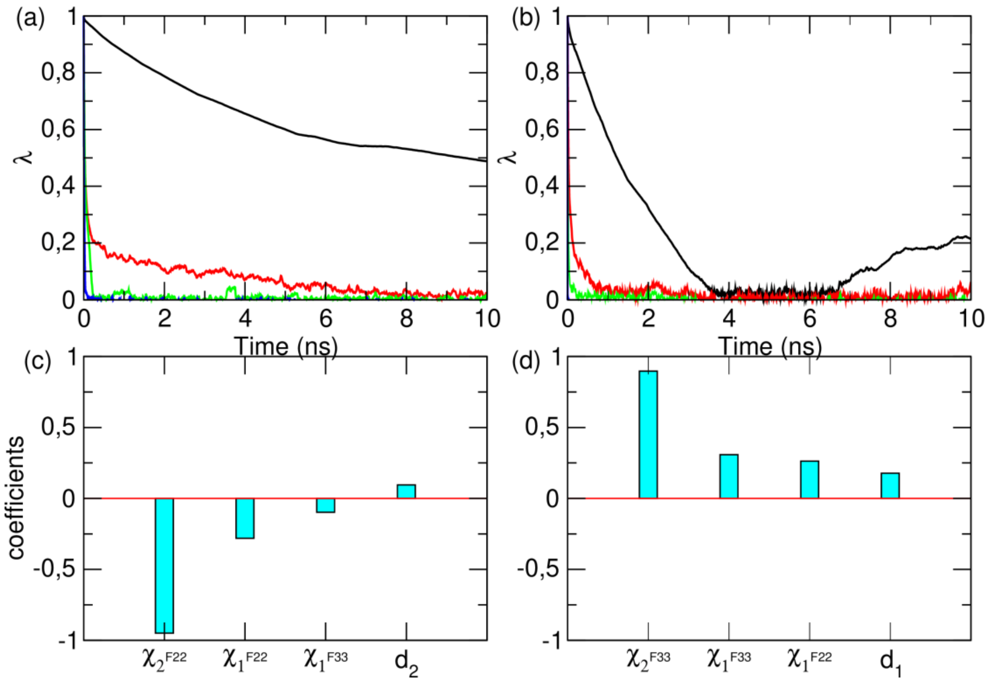

Figure S47. The decay of eigenvalues with respect to lag time values for F22 and F33 (figures (a) and (b), respectively). The coefficients of descriptors defining eigenvectors S_1^{F22} and S_1^{F33} (figures (c) and (d), respectively). The coefficients were chosen based on the maximum spectral gap value between the first (black line) and second (red) eigenvectors. The third and fourth eigenvectors are shown with a green and blue line.

The optimal CV (S_1^{F22} and S_1^{F33}) used to perform infrequent metadynamics analysis are defined as $S_1^{F22} = -0.95\alpha\beta(\chi_2^{F22}) - 0.28\alpha\beta(\chi_1^{F22}) - 0.097\alpha\beta(\chi_1^{F33}) - 0.095d_2$ and $S_1^{F33} = 0.89\alpha\beta(\chi_2^{F33}) + 0.31\alpha\beta(\chi_1^{F33}) + 0.26\alpha\beta + 0.18d_1$. The cumulative distribution functions of transition timescales obtained by infrequently biasing optimal CVs are shown in **Figure S48** below.

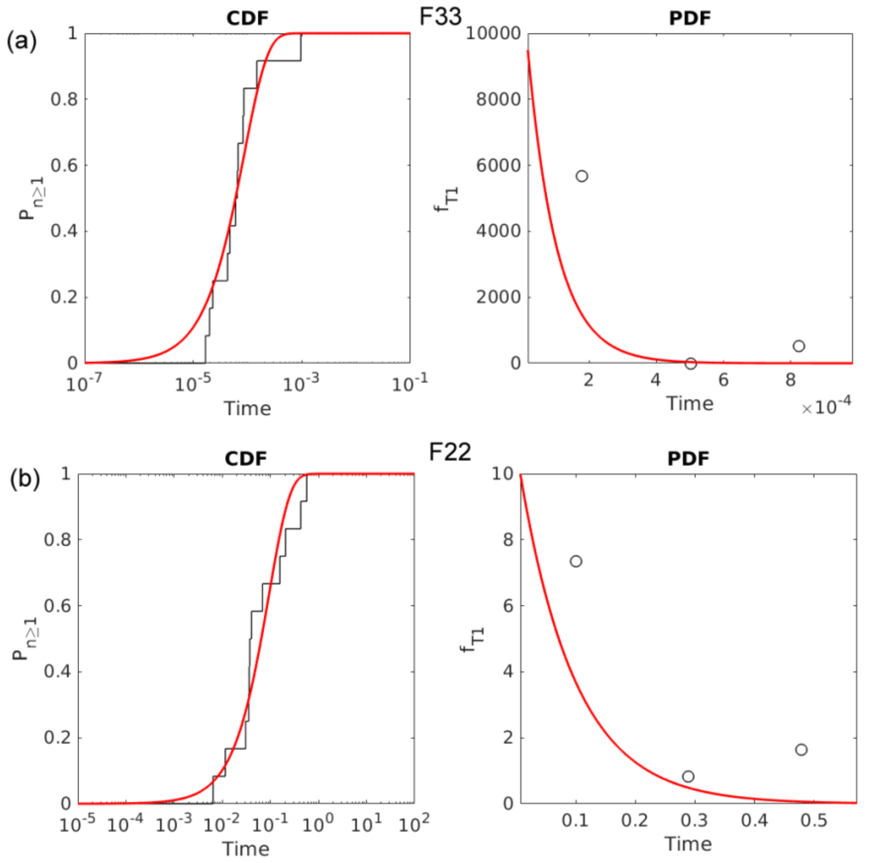

Figure S48. The cumulative distribution function of transition timescales obtained from VAC infrequent metadynamics simulations for (a) residue F33 (p-value = 0.70 and μ ln $2/t_m$ = 1.50), and (b) residue F22 (p-value = 0.49 and μ ln $2/t_m$ = 2.42).

Appendix II. Residue F22 state-to-state rates.

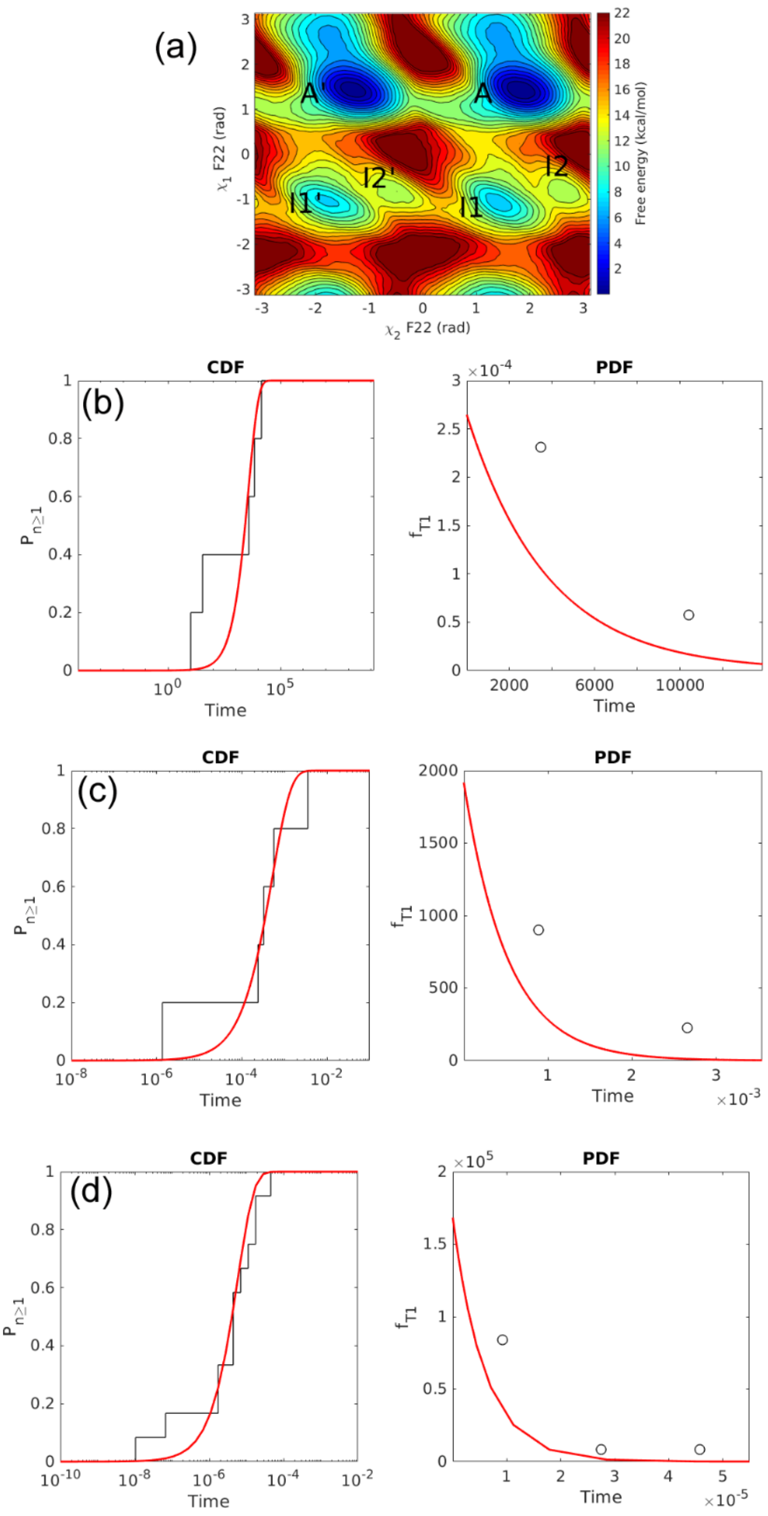

Figure S49. The Poisson fit to cumulative distribution function of transition timescales obtained from infrequent metadynamics simulations of (i) A to I1 transitions, (ii) I1 to A transitions, and (iii) I1 to I1' transitions.

Here, we report the results of infrequent metadynamics simulations starting from stable states A, I1 and directed symmetry-equivalent A' and I1' states of residue F22. The trajectories are directed to specific intermediates using harmonic wall bias (see Equation 3 in main manuscript). The details of wall bias parameters are provided below:

Table S5. k and s_0 for state-to-state directed InMetaD simulations of residue F22.

States	N_{traj}	CV	LOWER_WALL (s_0) (rad)	UPPER_WALL (s_0)(rad)	k (kJ.mol^{-1}.rad^{-2})
A → I1	5	χ_2	0.21	3.14	4184
I1 → A	5	χ_2	0.21	3.14	1000
I1 → I1'	12	χ_1	-2.25	-1.50	1000

The transitions to nearest neighbors are considered and five simulations were performed in each case. The symmetry of stable state positions on free energy surface allowed to build a complete transition rate matrix with fewer simulations. The infrequent metadynamics simulations are performed for (i) A to I1 transitions, (ii) I1 to A transitions, and (iii) I1 to I1' transitions. The less stable state I2 (and I2') are ignored in the present calculation. The results are reported in Table S4 below.

Table S6. Ring flip t_{flip}(s), rate k (s^{-1}), p-values, and μ ln 2/tm ratios for state-to-state infrequent metadynamics simulations.

States	t_{flip} (s)	k (s^{-1})	p-value	μ ln 2/t_m
A → I1	3.77×10^3	2.65×10^{-4}	0.33	0.89
I1 → A	5.20×10^{-4}	1.92×10^3	0.98	1.98
I1 → I1'	5.93×10^{-6}	1.69×10^5	0.39	1.29

The transition rate matrix based on these simulations is reported in Table S5. The diagonal values[3-4] of this matrix are set to negative of the sum of row elements. All eigenvalues are negative real values as suggested in reference 3. The dominant eigenvalue 83.0 s^{-1} was observed.

Table S7. State-to-state transition rate matrix (values in sec^{-1}) between nearest-neighbor states A, I1, I1', and A' of residue F22 (AMBER ff14SB) simulations.

Starting state (row) – end state (column)	A	I1	A'	I1'
A	0	2.65×10^{-4}	4.15×10^{1}	0
I1	1.92×10^{3}	0	0	1.69×10^{5}
A'	4.15×10^{1}	0	0	2.65×10^{-4}
I1'	0	1.69×10^{5}	1.92×10^{3}	0

References:

1. Fenwick, R. B.; Orellana, L.; Esteban-Martín, S.; Orozco, M.; Salvatella, X., Correlated motions are a fundamental property of β-sheets. *Nat. Commun.* **2014,** *5* (1), 4070.
2. Collaboration, T. A.; Robitaille, T. P.; Tollerud, E. J.; Greenfield, P.; Droettboom, M.; Bray, E.; Aldcroft, T.; Davis, M.; Ginsburg, A.; Price-Whelan, A. M.; Kerzendorf, W. E.; Conley, A.; Crighton, N.; Barbary, K.; Muna, D.; Ferguson, H.; Grollier, F.; Parikh, M. M.; Nair, P. H.; Günther, H. M.; Deil, C.; Woillez, J.; Conseil, S.; Kramer, R.; Turner, J. E. H.; Singer, L.; Fox, R.; Weaver, B. A.; Zabalza, V.; Edwards, Z. I.; Azalee Bostroem, K.; Burke, D. J.; Casey, A. R.; Crawford, S. M.; Dencheva, N.; Ely, J.; Jenness, T.; Labrie, K.; Lim, P. L.; Pierfederici, F.; Pontzen, A.; Ptak, A.; Refsdal, B.; Servillat, M.; Streicher, O., Astropy: A community Python package for astronomy. *A&A* **2013,** *558*, A33.
3. Schafer, N. P.; Hoffman, R. M. B.; Burger, A.; Craig, P. O.; Komives, E. A.; Wolynes, P. G., Discrete Kinetic Models from Funneled Energy Landscape Simulations. *PLOS ONE* **2012,** *7* (12), e50635.
4. Tiwary, P.; Limongelli, V.; Salvalaglio, M.; Parrinello, M., Kinetics of protein–ligand unbinding: Predicting pathways, rates, and rate-limiting steps. *Proc. Natl. Acad. Sci. U.S.A.* **2015,** *112* (5), E386-E391.
5. Brotzakis, Z. F.; Limongelli, V.; Parrinello, M., Accelerating the Calculation of Protein–Ligand Binding Free Energy and Residence Times Using Dynamically Optimized Collective Variables. *J. Chem. Theory Comput.* **2019,** *15* (1), 743-750.
6. Yang, Y. I.; Parrinello, M., Refining Collective Coordinates and Improving Free Energy Representation in Variational Enhanced Sampling. *J. Chem. Theory Comput.* **2018,** *14* (6), 2889-2894.
7. Tiwary, P.; Berne, B. J., Spectral gap optimization of order parameters for sampling complex molecular systems. *Proc. Natl. Acad. Sci. U.S.A.* **2016,** *113* (11), 2839-2844.
